# An Automatic Gait Feature Extraction Method for Identifying Gait Asymmetry Using Wearable Sensors

**DOI:** 10.3390/s18020676

**Published:** 2018-02-24

**Authors:** Arif Reza Anwary, Hongnian Yu, Michael Vassallo

**Affiliations:** 1 Faculty of Science and Technology, Bournemouth University, Fern Barrow, Poole BH12 5BB, UK; manwary@bournemouth.ac.uk; 2 Royal Bournemouth Hospital, UK, CoPMRE Bournemouth University, Fern Barrow, Poole BH12 5BB, UK; Michael.vassallo@rbch.nhs.uk

**Keywords:** inertial measurement unit, accelerometer, gyroscope, asymmetry, feature extraction, wearable sensors, gait analysis

## Abstract

This paper aims to assess the use of Inertial Measurement Unit (IMU) sensors to identify gait asymmetry by extracting automatic gait features. We design and develop an android app to collect real time synchronous IMU data from legs. The results from our method are validated using a Qualisys Motion Capture System. The data are collected from 10 young and 10 older subjects. Each performed a trial in a straight corridor comprising 15 strides of normal walking, a turn around and another 15 strides. We analyse the data for total distance, total time, total velocity, stride, step, cadence, step ratio, stance, and swing. The accuracy of detecting the stride number using the proposed method is 100% for young and 92.67% for older subjects. The accuracy of estimating travelled distance using the proposed method for young subjects is 97.73% and 98.82% for right and left legs; and for the older, is 88.71% and 89.88% for right and left legs. The average travelled distance is 37.77 (95% CI ± 3.57) meters for young subjects and is 22.50 (95% CI ± 2.34) meters for older subjects. The average travelled time for young subjects is 51.85 (95% CI ± 3.08) seconds and for older subjects is 84.02 (95% CI ± 9.98) seconds. The results show that wearable sensors can be used for identifying gait asymmetry without the requirement and expense of an elaborate laboratory setup. This can serve as a tool in diagnosing gait abnormalities in individuals and opens the possibilities for home based self-gait asymmetry assessment.

## 1. Introduction

Gait asymmetry (GA) is an indicator of different diseases and disease progression. It results in reduced gait efficiency and activity levels. Objective assessment of GA is important in the treatment and rehabilitation of patients with various conditions such as falls or after orthopaedic surgery. Gait is the result of a series of rhythmic alternating movement of arms, legs, and trunk which create forward movement of the body [1]. It relies on complex mechanisms depending upon the closely integrated actions of musculoskeletal, nervous system (central and peripheral), visual, vestibular, auditory systems; joint mobility and the smooth propulsive movement of the center of gravity. Every individual’s gait pattern should be symmetrical with right and left sides performing identical movements. This is not the case since every individual has a unique gait pattern and the limb movement of one side is not exactly repeated on the other side, which results in GA. GA analysis provides bilateral locomotive information of gait parameters (e.g., length and period of stride, step, stance and swing), kinematic and kinetic measurements (e.g., angular joint trajectories, angular joint velocities, joint forces, and reaction forces), muscular measurements (e.g., muscle contraction, muscle force) and energy expenditure (e.g., oxygen consumption, heart rate) [2]. This is employed in different domains such health, sports and rehabilitation. GA can be a determinant of recovery in patients with different diseases such as Parkinson’s disease [3] and stroke [4]. It can be used to monitor and improve an athlete’s performance [5] as well as a patient’s progress in orthopedics and rehabilitation [6]. In biometrics and biomedical engineering areas, gait analysis is used as an assistive tool to characterize human locomotion [7]. GA is important in elderly patient fall risk assessment [8], and is a predictor of functional and cognitive decline [9].

The tools and methodologies used to assess GA are often arbitrary [10] and often studied in non-natural controlled conditions. Currently available methods include reflective skin markers [11], ear-worn sensors [12] and camera based motion analysis systems [13]. Other methods for measuring GA include ground reaction forces [14], dynamic electromyography [15], and instrumented walkways [16]. These methods are laboratory based, complicated, costly, and often carried out by technical or clinical staff, which may be difficult for patients to use at their homes. 

Clinical scales used to analyse gait parameters are subjective or semi-subjective and a poor replacement to laboratory based methods for identifying changes in GA. Different assessment tools such as the Gait Abnormality Rating Scale [17], Figure of 8 Walk Test [18], Four Square Step Test [19], The Functional Gait Assessment [20], Groningen Meander Walking Test [21] and Berg Balance Scale [22], are used to observe a patient’s gait and balance. Their use has a high chance of intra and inters observer variation and human error. This may affect the accuracy of diagnosis, follow-up and treatment [2]. Therefore, a more objective way of assessing GA is required. 

A variety of wearable sensors including accelerometer, gyroscope, magnetometer, foot pressure sensor, inclinometer, and goniometer [23,24] are generally used to measure various characteristics of human gait. Inertial Measurement Unit (IMU) sensors are used in different situations such as monitoring of post-operative gait abnormalities [25], stride variability [24], measurement of GA [26], fall-related gait characteristics measured on a treadmill in daily life [27], nature of the Parkinsonian gait [28] and human waking foot trajectory [29]. Accelerometer based gait parameters such as times of stance, swing, single support and double [30]; stride and stance phase [31]; gait velocity, cadence and step length [32]; step number, moving distances, every step instant speed and average speed [33]; step counting [34,35]; times of heel strike, toe strike, heel-off, and toe-off [36]; stride length and duration [37]; walking distance, time and speed [38] were investigated. 

Although, IMU based gait analysis methods are available, specific GA was actually found in few studies [30]. Some studied a single gait parameter [24], single sensor [12] or applied simple statistical methods for comparisons [38]. Force-sensitive resistors placed in insoles to detect ground contact were used to estimate the stance time for GA [39]. Microsoft Kinect-based GA [13], IMU and pressure sensitive shoe insole was used to detect gait onset and toe-off detection [40] and IMU-based knee flexion/extension angle measurements [41] and GA using gyroscopes [42] were also used. Systems developed for the detection of GA require a fully automatic system for data collection, feature extraction and quantitative measurements where both limbs are evaluated. 

In order to broaden the use of accurate quantitative GA monitoring in clinical screening and research, an affordable GA tool is required which can be used in clinic or home. This study aims to design and implement an automatic lower limb gait features extraction method based on accelerometer and gyroscope data to increase the reliability and validity of monitoring GA. We set out to develop an affordable multi-sensor based synchronous data collection system for a comprehensive physical gait analysis extracting 24 commonly reported GA features. We develop a novel android app for collecting synchronous accelerometer and gyroscope data from both legs. Features include total distance, total time, total velocity, stride, step, cadence, step ratio, stance, and swing. We also estimate the mean, standard deviation, variance, minimum and maximum values. The paper has the following contributions: (1) designing and developing a novel android app to collect real time synchronous IMU data from legs; (2) proposing a gait asymmetry feature extraction method (a novel stride detection technique, a stance and swing detection technique, and a method for estimating travelled distance); and (3) creating a data set using our developed app and designed MetaWear casing, Velcro elastic belt, and buckles for validating the designed app and the proposed method. The paper is organized in the following sections: Section 2 presents the design and the methods of the proposed system. Section 3 delivers the experimental results and discussion. The conclusion is given in Section 4.

## 2. Design and Methods

### 2.1. Subjects Selection

We recruited a convenience sample of 20 subjects with 10 healthy young subjects (nine male, mean age 25.3 years, standard deviation 4.64, range 19–35 years), and 10 older subjects (nine male, mean age 69.4 years, standard deviation 7.28, range 62–86 years). Older subjects 1, 3, 5, 6, 9 and 10 did not have any health problems. Subject 2 has a right foot drop and drags the foot and toes. Subject 4 has pain in the right leg lower muscle and walks without any support. Subject 7 has pain in the lower part of his left leg and uses a crutch during walking. Subject 8 has pain in both ankles and walks with support of a walker. According to World Health Organization (WHO), the life expectancy at birth is 71 years in Bangladesh [43]. Therefore, 65+ is considered old age in Bangladesh although would be viewed as young old in the Western countries. The subjects are purposefully chosen for this study to provide a variety of gaits for evaluation.

### 2.2. Experimental Protocol and Calibration

The experiment is performed in two different locations for young and older. The older subjects are residents in a care home. All subjects perform a walk in a straight corridor comprising of 15 strides of normal forward walking, a turn-around and another 15 strides. Accelerometer and gyroscope data from sensors attached on two foot locations are recorded in a database synchronously using our Android app. The distance carried out by walking on the corridor is measured by a tape. The several older subjects perform less than 15 strides. Calibration was performed individually where the distance travelled is measured manually and the result compared to the output from the sensor. The Qualisys motion capture system (Qualisys AB, Kvarnbergsgatan 2, Gothenburg, Sweden) is used for calibration in the validation stage as well.

### 2.3. Sensor Placing Location

**Figure 1:**
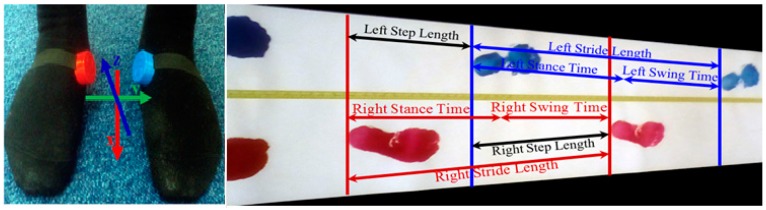
IMU sensors placement in right and left metatarsal foot locations of the barefoot.

From our investigation [44], it is found that placing a sensor in different foot locations gives quite different signal patterns. It is also observed that the orientation of the sensor has a significant effect on output data. In order to increase the sensor accuracy and reliability, and reduce the variability, all sensors are fitted tightly to the barefoot. We choose the barefoot rather than sensors attached to a shoe because wear and tear in the shoe can affect the position of the sensor and accuracy of the data output. The plantar aspect is not covered as this is the part of the foot in contact with the floor and is not practical for the subject to walk on the sensor. In this study, the sensors are placed at metatarsal foot locations of both legs (Figure 1) for collecting data since the best performance [44] can be achieved.

### 2.4. Sensor Selection

**Figure 2:**
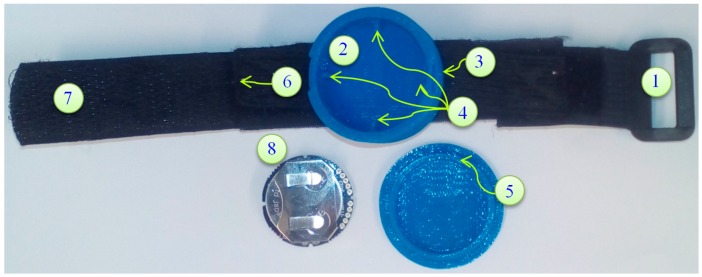
Proposed MetaWear casing, Velcro elastic belt, buckles and IMU sensor: (1) Buckle and Elastic Belt: the buckle is sewn onto an elastic belt for fastening to Velcro; (2) Bottom case which keeps the sensor safe from pressure, temperature and water; (3) Lock Open Edge which helps to open the cover from bottom case; (4) Sensor Lock Mechanism: The four locks keep the sensor sideways movement and orientation; (5) Cover Lock Mechanism which tightly locks with the case; (6) Velcro-Elastic Joint: The elastic belt is sewed with Velcro; (7) Velcro which adjusts and tighten when the sensor is attached; and (8) IMU sensor and battery.

There are wearable wireless IMU sensors commercially used for health rehabilitation and activity monitoring [45]. An affordable wearable Bluetooth, long autonomy, minimum consumption, multiple synchronized data transmission supported IMU sensor is important for our investigation. With all these considerations the recently introduced sensor MetaWear CPro (MBIENTLAB Inc., San Francisco, CA, USA) [46] (price: $30.00) was chosen. The power consumption of our sensor is low during sleep mode. The sensor (Figure 2) is in an active state when connected by Bluetooth to our android device and only goes to sleep mode once it is disconnected. It is sensitive to acceleration and rotatory movements that occur during normal human locomotion.

The IMU sensors are sampled at a frequency range of 20 Hz to 200 Hz [47]. In practice, a low sampling rate for the accelerometer possibly produces excellent recognition and accuracy in posture and activity classification [48]. For this study, the accelerometer range is ±8 m/s^2^ and gyroscope range is ±500 degrees/s with the sampling rate of 50 Hz. A casing for the sensor is designed using SolidWorks [49] and printed using a 3D printer. A Velcro elastic belt and buckles are used to adjust and attach the sensor (Figure 2).

### 2.5. Android App Design, Development and Data Collection

**Figure 3:**
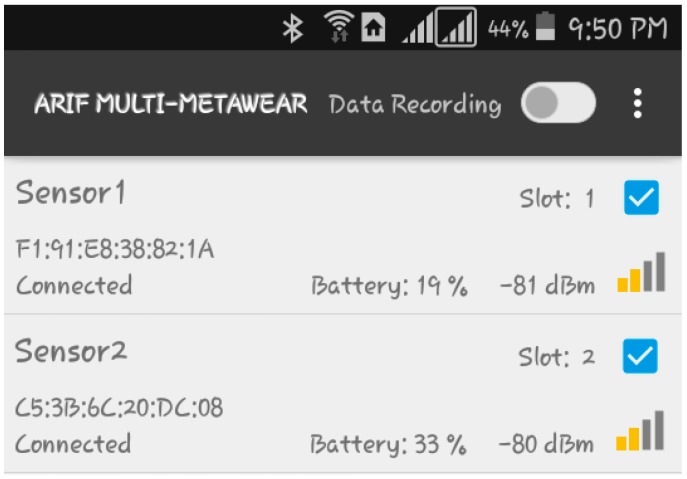
Proposed android app to collect data from MetaWear CPro.

The sensor provides an Android API library for interacting with the MetaWear board on an Android mobile phone. A minimum of Android 4.3 (SDK 18) is required to use this library, however some features will not properly function due to the underlying Bluetooth LE (BLE) implementation. For getting the best results, it is recommended to use an Android 4.4 (SDK 19) or higher and BLE 4.0 or higher. Based on these criteria, an Android app is developed using Android Studio 2.2 to collect real time accelerometer and gyroscope data, and store data on an external SD card in a csv file. The data soring format in a csv file is date (dd/mm/yyyy), time (HH:MM:SS.ss), system clock (milliseconds), accelerometer (X, Y, Z) and gyroscope (X, Y, Z), shown in Figure 3.

The app is designed through the following steps: (1) requirement analysis through the literature review and interview with the expert and users; (2) reviewing the market available software development platform; (3) initial design of the mobile app using Android Studio 2.2; (4) testing and debugging with the user feedback for improvement; (5) documentation for development. We develop the proposed app shown in Figure 3. The details of the design are beyond the scope of this paper and will be reported in the future.

Pressing the SCAN button the availability of all the devices for data collection is checked. The order of data collection is selected by Slot number. Each sensor then automatically connects with the corresponding mac address by showing CONNECTED. Pressing the DATA RECORDING switch makes a dialog box to get the file name for storing data. Pressing the OK button starts data collection. Pressing the STOP button stores the collected data.

**Figure 4:**
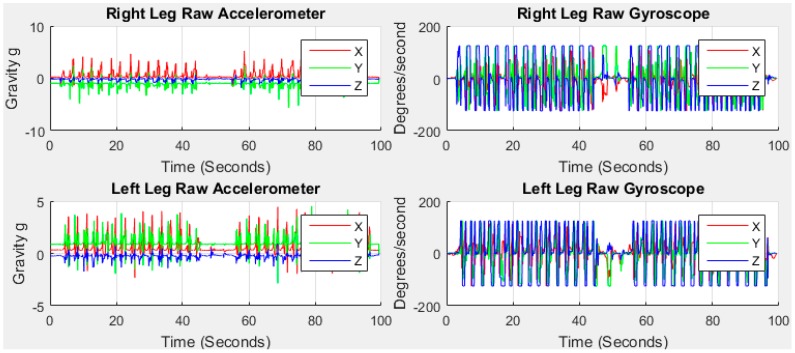
Raw accelerometer and gyroscope data from right and left feet of older subject 1.

A stride is a whole gait cycle with stance and swing events. This horizontal movement produces high acceleration during walking and this movement is the subject of investigation in this study for GA monitoring. The data with the horizontal movement information from the feet are analysed using our method to find GA information. Figure 4 shows the raw data.

The red, green and blue lines in Figure 4 stand for accelerometer readings on *x*, *y* and *z* axis, respectively, with *g* units (9.81 m/s^2^) in the sensor frame. We can observe from the raw data that the accelerometer reading on *x* is the highest and *z* is the lowest before the commencement of walking for right leg indicative of the initial gravitational force. Similarly, the accelerometer reading on *y* is the highest and *z* is the lowest before the commencement of walking for left leg. The initial data is not aligned to zero means that the sensors are not placed perfectly upright position with the earth frame in the foot locations due to the initial gravity part of *y* and *z*. For this study, the sensors do not need to be perfectly upright which in any case is not user-friendly and impossible. The discrepancy between the sensor frame, the foot frame and the earth frame are compensated for in this study.

### 2.6. Gait Asymmetry Feature Extraction Method

#### 2.6.1. Raw Data Processing

It is noted that accelerometers are sensitive to altitude and impact forces, while gyroscopes are sensitive to temperature changes and suffer from a low-changing bias. Consequently accelerometers have poor dynamic features and gyroscopes have poor static features [50]. To provide a robust absolute orientation vector in the form of quaternion or Euler angles, the sensor combines the measurements from 3-axis accelerometer and 3-axis gyroscope sensors. The algorithm in [46] fuses the raw data in an intelligent way to improve each sensors output. This includes offset calibration of each sensor, monitoring of the calibration status and Kalman filter fusion to provide distortion-free and refined orientation vectors [46]. The IMU sensor provides accelerometer *A*(*a_x_*, *a_y_*, *a_z_*) and gyroscope *G*(*g_x_*, *g_y_*, *g_z_*) with respect to time *t*. As the accelerometer is sensitive to acceleration due to movement and the local gravitational force, the input data consists of the user acceleration and gravitational acceleration. 

#### 2.6.2. Coordinate Systems

**Figure 5:**
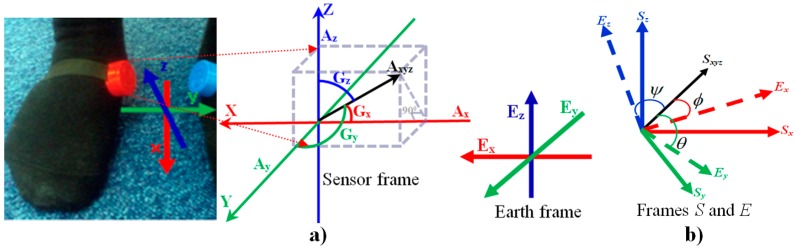
(**a**) Sensor frame and earth frame of accelerometer and gyroscope axes; (**b**) The orientation of frame *E* is achieved by a rotation, from alignment with frame *S*, of angle of *φ*, *θ*, and *ψ* around the axis *S_xyz_*.

In this study, there are three coordinate systems, the foot frame describing the foot rotation, the sensor frame describing the motion of the sensor and the global or Earth frame. Since the sensor is attached to the foot tightly using an elastic Velcro belt, we assume that the sensor does not slip or move during walking time. Therefore we consider that the foot frame and sensor frame are the same. Our approach is to transfer the sensor frame to the Earth frame and then to remove the gravitational component. The high gravitational force of the Earth frame is downward towards Earth. The *A_x_* axis is aligned along the foot axis of the IMU sensor, *A_z_* points downwards so that it is aligned with gravity so that the three axes from a right handed coordinate system shown in Figure 5a.

#### 2.6.3. Quaternion

Quaternion is a concept related to the foundations of algebra and number theory. While the accelerometer and gyroscope sensors enable the tracking of translational and rotational movements, the accurate measurement of the sensor orientation is important to interpret sensor information. Quaternions are a mathematical construct that consist of four individual numeric complex number components that can be used to represent the orientation of a ridged body or coordinate frame in a three dimensional space. 

Many quaternions are available to estimate the orientation from accelerometer, gyroscope and magnetometer data. We use the Madgwick technique [51] which fuses accelerometer, gyroscope and magnetometer for estimating quaternion. An arbitrary orientation of frame *S* relative to frame *E* can be achieved through a rotation of angle θ around an axis of *S_xyz_* defined in frame *E* shown in Figure 5b where the mutually orthogonal unit vectors *S_x_, S_y_, S_z_* and *E_x_, E_y_, E_z_* define the principle axis of coordinate frames *S* and *E*, respectively. 

*S_x_*, *S_y_* and *S_z_* define the components of the unit vector *S_xyz_* in the *x*, *y* and *z* axes of the frame *S*. To denote the relative frames of orientations and vectors, q^ES in Equation (1) represents the orientation of frame *E* relative to *S* and *S_xyz_* is a vector described in *S* [51].

(1) q^ES=[q1q2q3q4]=[cosθ2−Sxsinθ2−Sysinθ2−Szsinθ2]

**Figure 6:**
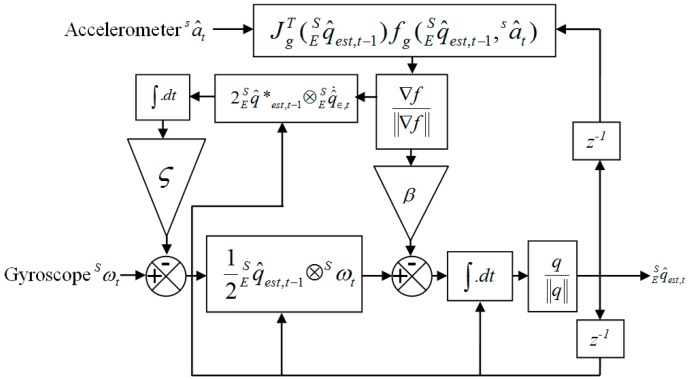
The process diagram of the complete orientation filter for an IMU.

The compensated gyroscope measurement ωcS then is used in place of the gyroscope measurements ωS, where the magnitude of the angular error in each axis ω∈S is equal to a quaternion derivative of unit length and then the integral gain ς directly defines the rate of convergence of the estimated gyroscope bias ωbS expressed as the magnitude of a quaternion derivative [51]. The complete orientation of q^est,tES is achieved and Figure 6 shows a block diagram representation of the complete orientation filter implemented for an IMU. The details derivation is described in the Supporting Information.

**Figure 7:**
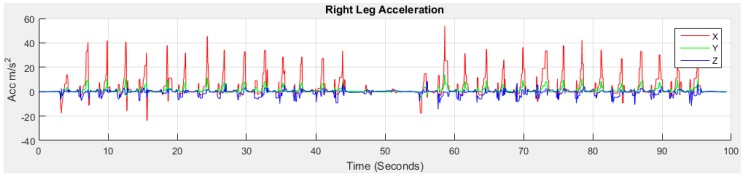

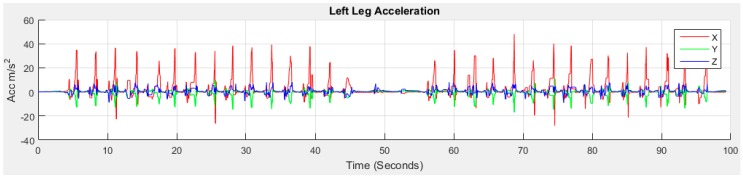
Acceleration due to user movement *AM_xyz_* after removing gravity component.

We apply the technique shown in Figure 6 to our collected data for body acceleration to the Earth frame with a sampling frequency of 50 Hz, *β* gain of 0.1 and *ς* gain of 0.5. The gravity components are removed and the conversion of the accelerometer from gravitational force *g* to user acceleration of movement (*AM_xyz_*) m/s^2^ is achieved by multiplying 9.81. The three axis data are transformed due to the fact that looking at specific axes is sensitive to the sensor orientation [52]. Figure 7 shows the acceleration due to user movement *AM_xyz_* = [*am_x_*, *am_y_*, *am_z_*] for both feet of older subject 1.

**Figure 8:**
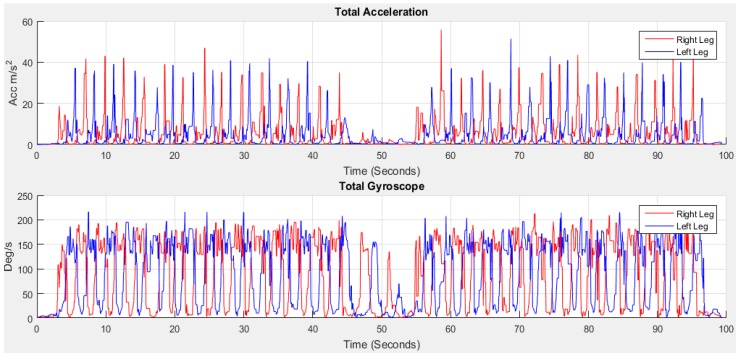
The total acceleration *AT_xyz_* and gyroscope *GT_xyz_*.

Figure 8 shows the acceleration of total *AT_xyz_* and gyroscope *GT_xyz_* towards *x*, *y* and *z* directions estimated using Equation (2):

(2) $$\left|AT_{xyz_{i}}\right|=\sqrt{{am_{xi}}^{2}+{am_{yi}}^{2}+{am_{zi}}^{2}}\;\mathrm{and}\;\left|GT_{xyz_{i}}\right|=\sqrt{{g_{xi}}^{2}+{g_{yi}}^{2}+{g_{zi}}^{2}}$$

#### 2.6.4. Stride, Stance, Swing and Step Events Detection

**Figure 9:**
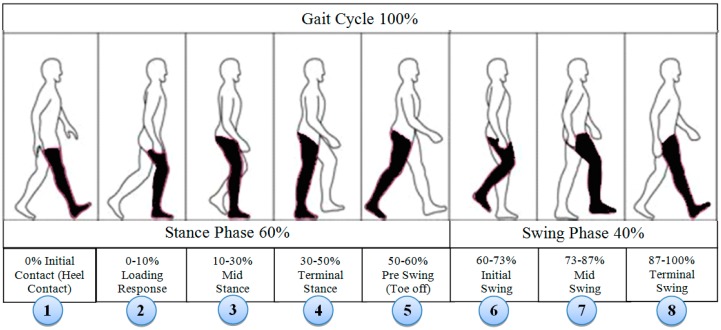
Normal human gait phases [53].

Human walking can be described and characterized in the context of a gait cycle. A stride is the distance between a point on one foot at the first foot contact and the same point on that foot at the next foot contact. It is the equivalent of a gait cycle made up of two steps. Each stride contains stance and swing relevant phases. Stance and swing phases of a gait cycle consists eight relevant phases shown in Figure 9.

**Figure 10:**
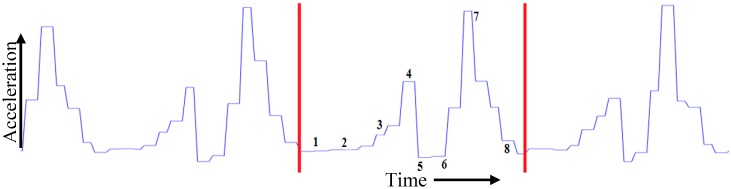
Eight different phases of a gait cycle from accelerometer data.

The first phase starts when the heel contacts the ground and the waist is in its lowest position during the entire step. There is deceleration of the leg towards the horizontal axis as the velocity moves to zero. The zero velocity remains until the terminal stance phase where the foot is flat on the ground. The next phase is pre-swing where the toe is off the ground and starts forward movement demonstrating initial acceleration towards horizontal axis. The swing phase is when the heel moves off the ground. The acceleration interval corresponds to the change from the heel lift to the swing at the height point at mid-swing phase. Deceleration starts during the terminal swing phase from the highest point to the foot back flat on the ground. There is zero velocity again in the interval corresponding to the change from a flat foot to a heel lift. These different phases of gait cycle presented in Figure 10 are identifiable from the IMU acceleration signal. The same phenomenon of human limb kinematic with accelerometer signal output during a typical walking cycle has been identified in the literature. Our gait cycle accelerometer signal *AT_xyz_* (Figure 10) is agreed with the signal pattern in [53,54]. The different phases of the gait cycle (Figure 9) with corresponding accelerometer signal are shown in Figure 10.

From Figure 10, we can observe that at the start and end of each stride, the walker’s feet are stationary on the ground. As the IMU sensor is attached to the foot, the stance phase is stationary and swing phase is non-stationary. Due to the walker’s movement towards the *x* axis, the acceleration shows its highest value in the swing phase. The output of the accelerometer signal will be different, if the sensor is placed on foot [54,55], waist [56] or different body locations [34,57]. 

**Figure 11:**
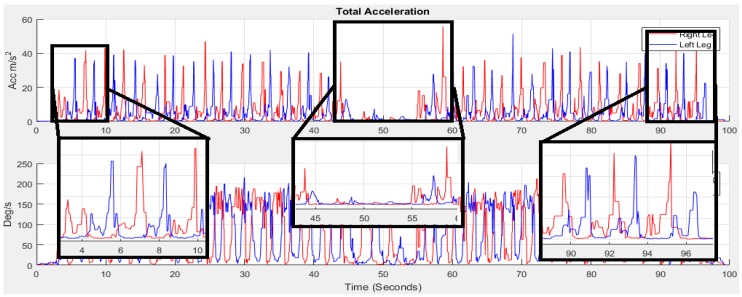
Peaks magnitude variation from Figure 8.

Many algorithms [34] are available for stride event detection from IMU sensors. During human walking, a consistent sequence of motions is performed at each stride that results in a maximum peak value that lies in the mid-swing phase. This mid-swing phase appears when a user pushes off this foot and shortens the limb to clear ground thus releasing the foot from the ground until it again contacts with ground as shown in Figure 9 and Figure 10. A particular threshold value is set to detect these characteristics for detecting stride [35,58]. One disadvantage of these algorithms is that any motion with a similar periodicity of walking will trigger for a false stride event. In addition, difficulty arises in finding the automatic selection of the threshold value which can vary between users, surfaces and shoes [59]. The variation in the peak magnitude gets larger for faster human waking velocities [57] and a window based threshold calculation [58] was used to obtain an acceptable level of accuracy for a larger window size. However increasing the window size may degrade the step detection accuracy during the translation of step mode because the threshold calculated from a larger window may not be able to effectively handle the variation in the recent statistics [57]. Due to peak magnitude variation, the threshold value also varies based on individuals walking style and even differs from left to right leg as shown in Figure 11.

**Figure 12:**
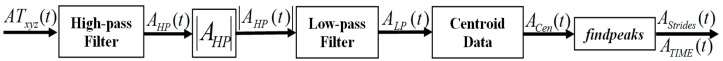
The proposed step detection technique.

The different threshold may result in a different output of detecting steps. Another important point is that when a subject begins walking from a standing state, stops walking for a turnaround or stops, there is poor acceleration and it is crucial to detect the gait cycle in these situations. For this reason the 1st stride is not considered for gait analysis by researchers [60]. We take this in consideration to address this point in this study. As the mid-swing phase in accelerometer data is a good indicator for performing a complete gait cycle, thus for counting the number of strides, the number of mid-swing phase in accelerometer data is analysed as walking strides are equal to the number of mid-swing phases. The highest peak is occurred at the push off phase starting from the terminal stance at the 4th to pre-swing at 5th phases shown in Figure 9 for gyroscope data. We apply threshold based algorithms obtaining low accuracy to detect the stride number for our collected accelerometer and gyroscope dataset. As the peaks at terminal stance phase are more prominent than the mid-swing phase, the threshold based algorithm detects two strides instead of single stride. To avoid this, a novel stride detection technique is proposed based on the local minimal prominence characteristics of strides associated with the time-varying magnitude of acceleration shown in Figure 12. The technique consists of designing a high-pass filter, computing the absolute value, designing a low-pass filter, shifting data to centroid and finding the strides using *findpeaks* [61] function.

The accelerometer converts acceleration to an electrical signal and in the process, unwanted constant bias in acceleration becomes a linear error called drift. Thus the 2nd order Butterworth digital high-pass filter with a sampling rate *fs* = 50 Hz and cut off frequency *fc* = 1000 Hz is applied to *AT_xyz_* to remove the corrupted data and the DC component of the acceleration signal. The smoothness is achieved at the price of decreased roll off steepness. The output from the filter is then passed to the zero phase *filtfilt* delay filter. The *filtfilt* corrects for phase distortion introduced by a one-pass filter [62]. The output of *filtfilt* filter then is passed through a low-pass filter with *fc =* 5 Hz to obtain *A_LP_* which is shifted to centroid using Equation (3).

(3) $$A_{Cen}=A_{LP}-mean(A_{LP})$$

**Figure 13:**
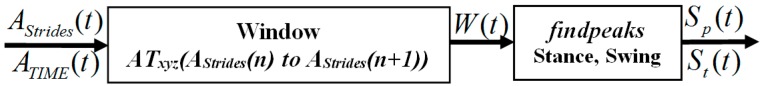
Proposed stance and swing detection technique.

To find the local minima prominences, *A_Cen_* is passed through a *findpeaks* function which finds local peaks in the data vector and ignores small peaks that occur in the neighbourhood of a larger peak. A local peak is a data sample that is either greater than its two neighbouring samples or is equal to Inf. If a peak is flat, the function returns only the point with the lowest index. The *findpeaks* function detects the stationary periods when the foot touches the ground the point of minimal prominence during walking. The function returns two vectors containing the minimal local peaks *A_Strides_* and the locations *A_TIME_* at which the peaks occur. The number of strides is the same as the length of *A_Strides_* vector. Again, as each stride consists of stance and swing events, thus the initial contact and the transition between pre-swing and initial swing (4th and 5th phases in Figure 9) are detected using steps in Figure 13 to get stance and swing information.

**Figure 14:**
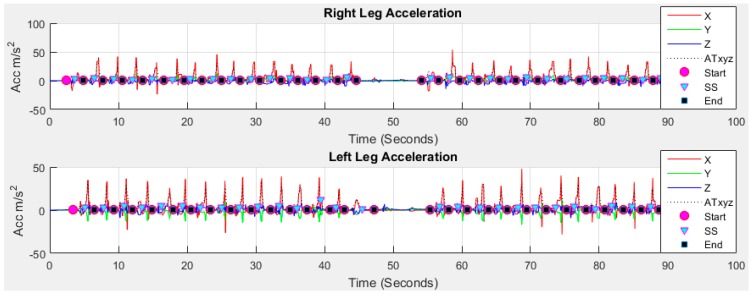
Result of stride, stance and swing event detection using proposed method.

A *window* is prepared whose size is the difference between a pairwise consecutive strides from *A_Strides_*. Each *window* is then passed through *findpeaks* function as there is only one local maximum in each stride located between 4th and 5th phases in Figure 9. A loop from 1 to total detected strides number is used to find the stance and swing event for all strides. The detected *Start* (purple circle), *SS* (cyan triangle) and *End* (black rectangle) information of each stride are shown in Figure 14 for right and legs where the stance phase information is provided by the difference between *Start* and *SS*; and the swing information is the difference between *SS* and *End*.

**Figure 15:**
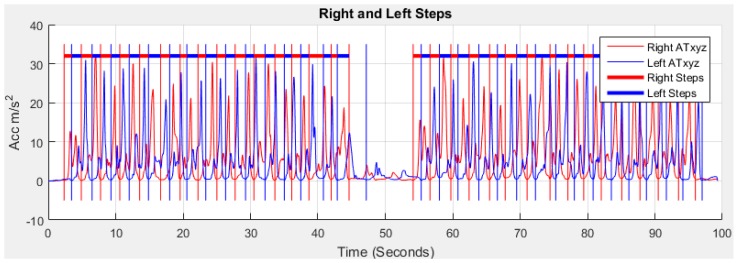
Result of step event detection using proposed method.

A step is the sequence of events between the contact of one foot and the next contact of the opposite foot. At the beginning of the stance phase, the initial contact of the foot contacts with ground of the one leg. The loading response begins at the initial contact and ends when the toe of opposite leg leaves the ground, midstance then begins and finishes when the center of gravity is over the same foot. The terminal stance begins when the center of gravity is over the supporting foot and ends when the opposite leg contacts the ground. The strides, stance and swing event are detected from right and left legs. The step event is then detected between the heel of two subsequent feet shown in Figure 15.

#### 2.6.5. Velocity and Distance Estimation

A pedestrian navigation system (PNS) has diverse applications in airports, theatres, underground parking, other indoor and outdoor places and modern cities. In order to estimate PNS in a virtual environment, a number of navigation methods [63] are available to derive pose estimates from electrical measurements of mechanical, inertial, acoustic, magnetic, optical, and radio frequency sensors. There are popular conventional approaches of IMU-based PNS known as Pedestrian Dead-Reckoning (PDR) solution and inertial navigation system (INS) [64]. In PDR, a constant step length is assumed on a relatively smooth surface, often usable in office-like environment and the average step length is integrated along with the orientation estimations obtained from IMU at each detected step to track the position. IMU based foot mounted PNS methods and systems have been proposed in [55,65]. The foot mounted method uses a double integral on horizontal acceleration to estimate distance and a gyroscope or magnetometer to measure the heading distance [64]. The disadvantage of PDR is that it assumed on relative smooth surfaces and a constant step length that needs tuning for individual users. Again, if the walking pattern is different from the predefined step length model, this may adversely affect the distance estimation [64]. The INS is adapted from the aerospace community in which IMU is used for tracking position, velocity and attitude [55,66,67]. Sensor drift is a well-known problem in INS system which depends on the precision of the sensor used and high end inertial sensors are very costly [66]. The IMU sensors have some small errors when estimating the distance and direction and signal noise can further exacerbate this problem described details in [64,66].

In this paper, we consider the walking constrains of a user with an IMU fitted on the both right and left legs. We apply appropriate methods to detect the movement of the leg, changes in position and compute its velocity and travelled distance from the initial location by means of the data collected from the accelerometers. The basic approach lies on the double integral of the accelerometer data where the first applying integration retrieves the current velocity and then the second applying integration computed on the velocity provides the distance travelled. Distance travelled is obtained principally from trapezoidal double integration [68] of the user movement signal on each stride detected in the direction of travel as mentioned in Section 2.6.4. However, there are two main problems for performing a double integration of the acceleration signal, unknown initial condition and drift. The unknown initial condition problem means integration requires a known initial condition. Drift means IMU sensors are subject to errors in acceleration that when integrated in to velocity and distance, leads to drastic integration error. This can be unbound over time if the acceleration signal is integrated without filtering [55,64,65,66,68]. The integration works properly with known initial conditions. Thus, to calculate the actual displacement, integration errors must be minimized. A method known as zero-velocity update (ZUPT) [64,66,67] is often used to correct for drift and is often used to aid in autonomous inertial pedestrian navigation. ZUPT uses the fact that during human walking time, one foot is always stationary on the ground. When a stationary period of the acceleration is detected the assumption is made that the foot is on the ground and the velocity at that time is set to 0. In this way, the drift is greatly reduced. However, ZUPT assumption implies that the angular rate is 0 as well and consequently if the accelerometer is moving at a constant velocity, the algorithm would misjudge the motion as stationary. ZUPT therefore cannot reduce all errors [50,66]. Based on our experience, an accelerometer is very sensitive to movement and walking is a complex course of acceleration and deceleration. The detection of zero velocity does not fail due to misjudgement, but adjusting the threshold value for motion detection plays an important role in that misjudgement when motion detection is not properly set (discussed and showed in Section 2.6.4).

**Figure 16:**
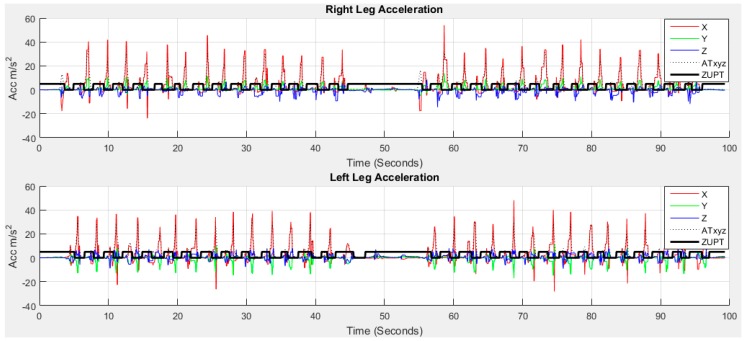
Zero-velocity update (ZUPT) from $\left|AT_{xyz_{i}}\right|$.

In addition, this issue may not relevant to this study as the “foot stationary event” is already detected based on local minimal prominence as described in Section 2.6.4. The stationary period remains in the stance phase and the movement period remain in the swing phase shown in Figure 9. As IMU sensors are mounted on each foot, the acceleration is high in the swing phase due to the movement of the leg during walking. The zero-velocity in non-stationary period of stance phase is used in the ZUPT scheme to reduce the drift. The ZUPT based on local minimal prominence to detect the swing phase is shown in Figure 16.

**Figure 17:**
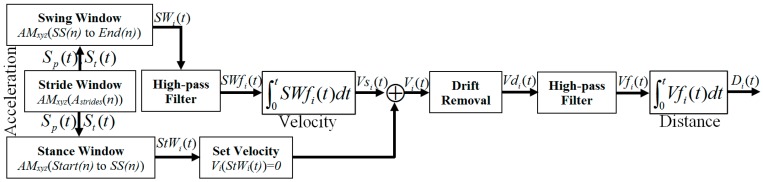
Proposed method for estimating travelled distance.

Another concern regarding the double integration is that the displacement signal emphasizes the low frequency data more than the acceleration signal, a low-pass filter effect of the integrator. Therefore, the input data are passed through a high-pass filter to remove the direct component of the acceleration signal. Considering these issues, a double integral method shown in Figure 17 is proposed for calculating travelled distance. 

**Figure 18:**
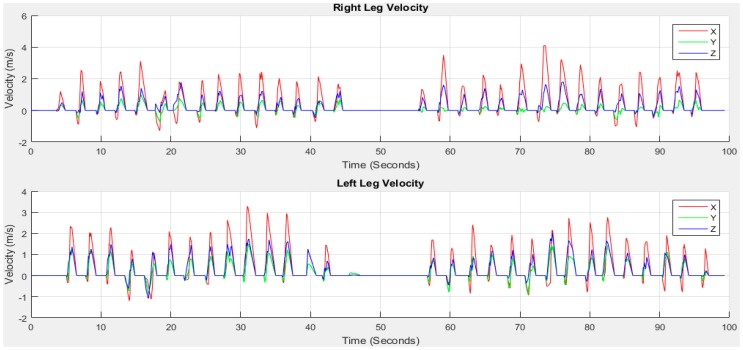
First integral operation to get velocity *V_i_*(*t*).

In order to obtain the velocity and distance in time series, two stages of integration and two stages of high-pass filtering are applied. A stride window is prepared from *A_Strides_* and *AM_xyz_*. The swing and stance windows are brought out from the corresponding stride window using *Start*, *SS* and *End* mentioned in Section 2.6.4. A 1st order Butterworth high-pass filer is designed with *fs* = 50 Hz and *fc* = 1000 Hz. The 1st integral operation *cumtrapz* in Matlab is applied on *SWf_i_* with respect to time *t* that gives the *Vs_i_*(*t*) velocity for the 1st swing phase. The ZUPT is applied on stance phase to set the stationary velocity to 0. The non-stationary period of swing velocity and stationary period of stance zero velocity are then combined to obtain *V_i_*(*t*) shown in Figure 18.

As the stationary period in stance phase velocity is set to zero, the integral constant from non-stationary period in swing phase exists in *V_i_*(*t*). Therefore, it is important to remove the drift caused by integration from *V_i_*(*t*). To remove the integral drift [50], the velocity difference between the initial and end of a non-stationary period is estimated. The velocity difference is then divided by the number of samples during this non-stationary period to get the drift rate. The drift rate is multiplied with the corresponding data index to estimate the drift value at that certain point. The drift value is then subtracted from the calculated velocity *V_i_*(*t*) to obtain the error free velocity *Vd_i_*(*t*). *Vd_i_*(*t*) is then passed through the high-pass filter for the 2nd time and the distance *D_i_*(*t*) is estimated after 2nd integral operation. *D_i_*(*t*) consists of the distance towards *x*, *y* and *z* coordinates. Repeat the same procedure for all strides to calculate velocity and distance. Then estimate the travelled distance using Equation (4):

(4) $$TD_{xyz_{i}}=\sqrt{{D_{x_{i}}}^{2}+{D_{y_{i}}}^{2}+{D_{z_{i}}}^{2}}$$

**Figure 19:**
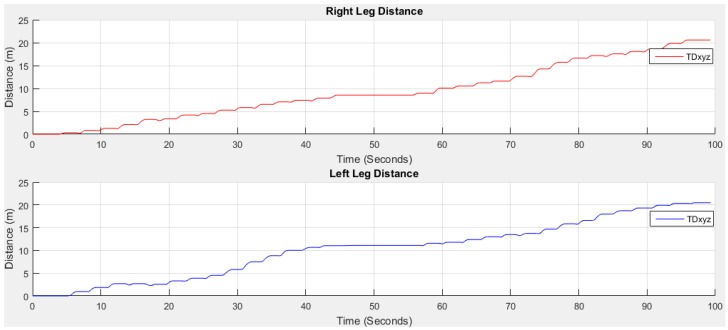
2nd integral operation to get distance *D_i_*(*t*).

Figure 19 shows the estimated distance *D_i_*(*t*) towards *x*, *y* and *z* and travelled distance *TD_xyz_*.

#### 2.6.6. Selection of Gait Asymmetry Variables

**Figure 20:**
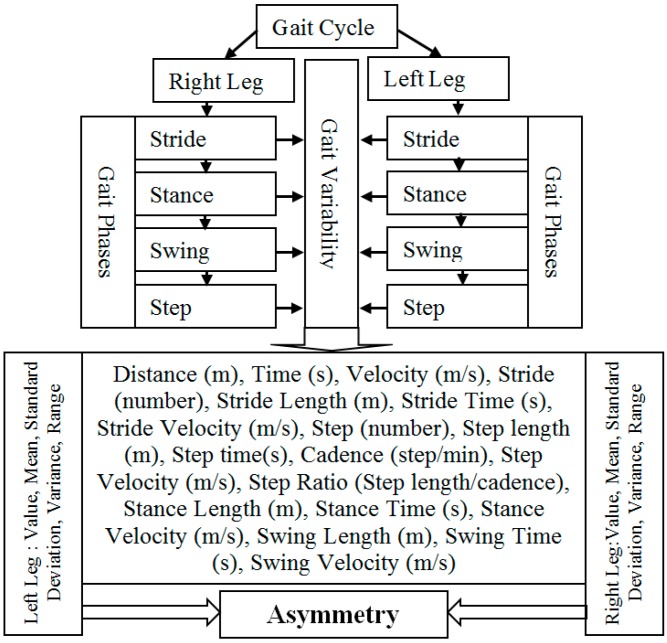
Proposed variability monitoring for GA.

GA in this study is considered as an indicator to show the difference between right and left leg walking which may serve as a diagnostic tool for clinicians. There is no commonly accepted superior guideline, preferred methodology or protocol for GA evaluation. The European GAITRite Network Group has developed Guidelines for Clinical Applications of Gait Analysis [69] to provide guidance to clinicians who implement spatiotemporal gait analysis to the clinic. Two issues are addressed in [69]: (1) Environmental measurement conditions and safety issues describe lighting, noise, visual distraction, clothing, footwear and safety; (2) Measurement procedures describe steady-state gait at different velocities, standardized walking instructions, assistive devices, stride-to-stride variability, gait analysis in association with simultaneous cognitive tasks and description of study population. To evaluate stride-to-stride variability, they recommend the highest possible number of gait cycles from a practical standpoint, with a minimum of three consecutive gait cycles for both left and right sides (i.e., a total of six gait cycles) [69]. Many issues relevant in GA assessment is reported in [69]. However there is no recommended systematic procedure for developing a GA assessment. General measurement of gait variability includes cadence, stride length and gait velocity [70], alone or in combination with other outcome measures such as stride to stride variability assessed by an accelerometer, gyroscope and magnetometer [24,36,39,40]. Researchers presented a set of 31 gait variables in [71] and 16 variables were investigated. Stride-to-stride variability [72] is commonly used to quantify walking consistency which is strongly associated with motor ability [73], mild cognitive impairment [74], dementia [75] and stroke [76]. In our study, a set of 24 commonly reported physical gait variables are initially considered for this analysis from both right and left legs. Figure 20 shows the GA variables.

**Figure 21:**
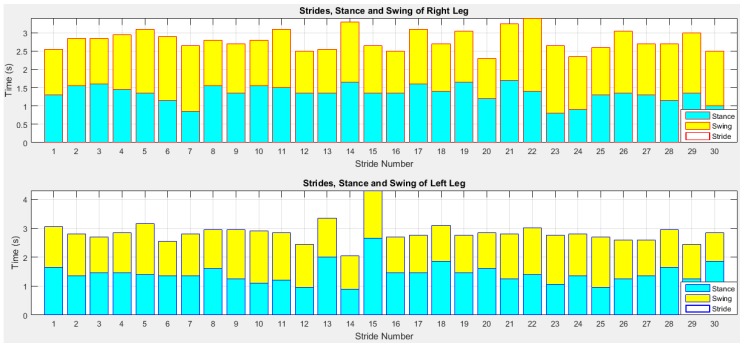
Stride, stance and swing information of right and left legs.

A stride length is the distance between the heel contacts of the same foot and a stride time is the interval between sequential initial heel contacts by the same limb. A step length is the distance and a step time is the interval from one foot strike to the other foot strike. The number of full steps taken within a minute is known as cadence. A stance length is the distance between the heel contact and pre-swing phases and a stance time is the interval of stance length. As the IMU sensor is placed at the foot location, the stance length is stationary. During the rest gait cycle, the foot is off the ground as the limb is swung forward to begin the next stride referred as swing phase. A swing length is the distance between the initial swing and terminal swing phases and a swing time is the interval of swing length. Figure 21 shows the stride, stance and swing information of older subject 1.

In the normal gait and the gait in patients after disease or injury, a certain GA level should be considered as normal [77]. Structural GA in human movement in limb length without underlying disease or injury is present in 90% of the population with an average magnitude of 5.2 mm and limb inequality of 20 mm must be present before considering clinically significant [78]. 

**Figure 22:**
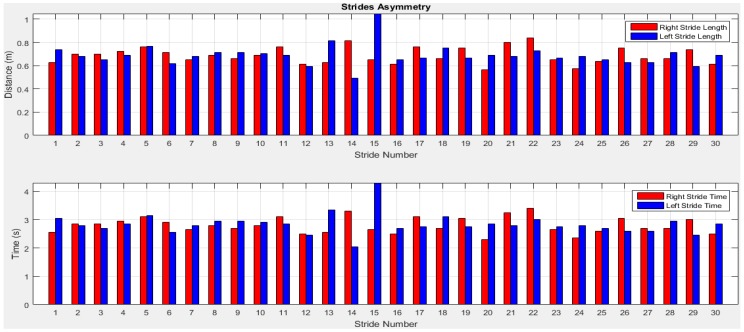
Stride asymmetry information of right and left legs.

**Figure 23:**
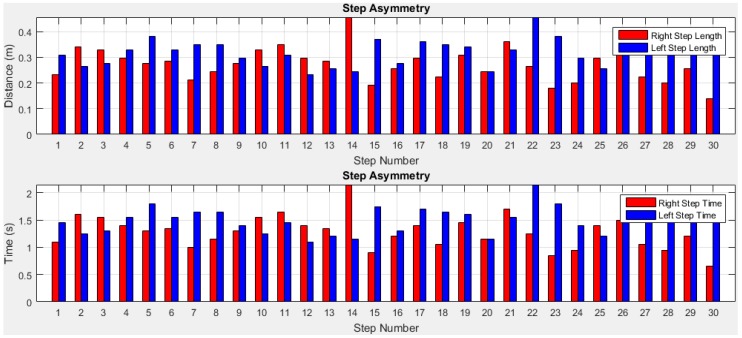
Step asymmetry estimation of right and left legs.

To quantify the temporal and spatial asymmetry of gait pattern, symmetry deviations (unaffected side − affected side, expressed as a fraction of the stride duration) [79], symmetric index (dividing the absolute difference of unaffected and affected by their average) [80], asymmetry ratios (1 − (affected/unaffected)) [81], Robinson symmetry index (2 × ((unaffected − affected)/(unaffected + affected)) × 100) [82], a log-transformed symmetry ratio (|100 × (*ln*(affected/unaffected))|) [83], and symmetry angles (([45° − arctan(affected/unaffected) × 100]/90) [84] were used. However quantifying indices have known limitations as described in [77]. A certain amount of GA is usually present in able-bodied individuals [77], but no agreement exists regarding the clinical criteria for quantifying of GA. In visual observation or self-reports of physical function, GA is frequently reported as present or not present which may not satisfy scientific criteria of reliability and validity [85]. Thus, an arbitrary cut-off value of 10% deviation from perfect symmetry was previously used as a criterion of asymmetry in gait assessment [76,82]. This was subsequently criticized due to its non-parameter specific nature [86]. Other previously used criteria to describe the presence or absence of GA included sensitivity and specificity [87], the use of 95% confidence intervals (GA within the limits of a 95% confidence interval (CI) obtained in a healthy population would define able-bodied gait, while GA outside the 95% CI would define pathologic gait) [86], and significant limbs difference [77]. In this study, we present gait variability quantities and validate the results with the ground truth for temporal GA monitoring. However, a parameter-specific criterion with optimal cut-off value that best discriminate GA from normal GA for each individual will be investigated in future study. The stride and step asymmetry information from older subject 1 is presented in Figure 22 and Figure 23.

#### 2.6.7. Statistical Analysis

Our estimated travelled distance and Qualisys results are tested for normality using Shapiro-Wilk [88]. The data is found to be normally distributed and a comparison of means is performed using the t-test for significance assuming equal variance. The estimated period and Qualisys results are found not normally distributed and a comparison of means were performed using the Wilcoxon Signed Ranks Test [89] for significance. The *p* values of <0.05 are considered to be significant for both analysis. Statistics are performed using SPSS Version 24 [90].

## 3. Experimental Results

Initial experimental results from older subject 1 (male, age 67, height 1.52 m and weight 68 kg) are presented. We extract automatic GA features based on the data collected from both feet. 

### 3.1. GA Results of Older Subject 1

Table 1 shows the accuracy of the distance travelled and estimated, detecting stride and step number from both legs.

(5) $$Accuracy=\left(100-\left|\frac{ActualValue-EstimatedValue}{ActualVale}\right|\times 100\right)\%$$

The accuracy is estimated using Equation (5). The actual distance travelled is 21.03 m measured using manual tape with 99.3 s walking time. The estimated both legs travelled distances are 20.59 m and 20.47 m. The actual and estimated distances are very close. Normal human walking velocity may vary from 1.5 to 2.5 m/s [91] and the walking velocity for this subject is 0.21 m/s which is slow. The accuracy of stride and step event detection are 100%. Table 2 shows the summery of average gait variability.

**Figure 24:**
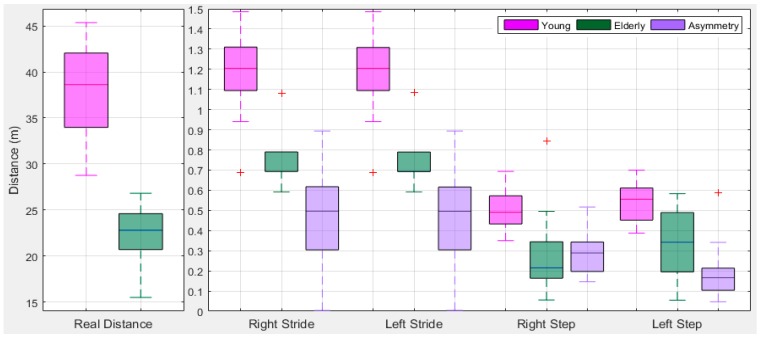
BoxPlot of stride and step asymmetry in distances from right and left legs.

**Figure 25:**
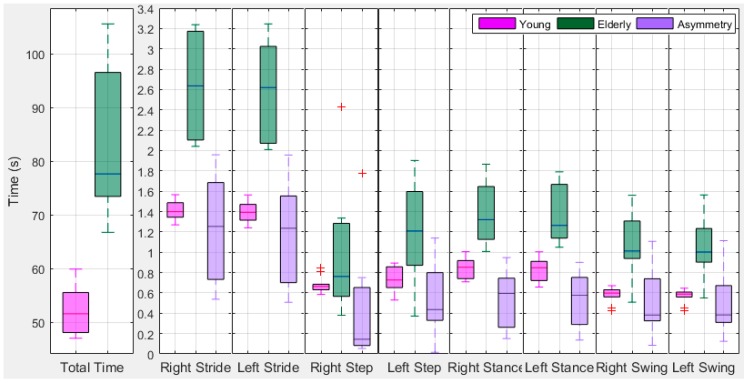
Box Plot of stride and step asymmetry in times from right and left legs.

We can observe from Table 2 that the mean stride lengths of both legs are the same. Although, the standard deviations are low and the right leg’s value is lower indicating that the left stride length has more variation compared to the right stride length. The highest stride length is found at the 15th stride (last stride) on the left leg which is before turning. The mean stride times are close for both legs. Although, the right and left leg stride length, time and velocity difference is low, Figure 24 shows that a little stride asymmetry is noticeable in right and left strides time and distance. The difference of other parameters between the legs is also low. However, it is noted from Figure 25 that step asymmetry is more prominent than stride asymmetry which may result in an inconsistent gait.

We validate our results using 10 young subjects (age average 27.55 ± 3.54) by conducting trials using the Qualisys Motion Capture System (Qualisys Medical AB, Gothenburg, Sweden) [92] and our IMU sensor concurrently. Applying our method to the collected data leads to the result in Table 3. The average accuracy of the result is 97.57% with 95% confidence interval 1.327 for the estimated distance and 99.01% with 95% confidence interval 0.266 for the Period.

Although the sample size is small, the significance of the test of normality for Qualisys and Estimated Distance are 0.83 and 0.37 using Shapiro-Wilk. The *t-*test shows that there is no difference in means (*p* = 0.094) between Qualisys (*μ*_1_ = 7.67, σ_1_ = 0.26) and Estimated Distance (*μ*_2_ = 7.49, σ_2_ = 0.39). There is a strong correlation (*r =* 0.81) present. The Wilcoxon Signed Ranks Test for Qualisys and Estimated Period shows no mean difference (*p* = 0.83).

### 3.2. GA Results of Young and Older Subjects

Table 4 shows the gait data from the 10 young subjects. Table 4 shows that the accuracy of estimating the total distance compared with the actual distance is also high for both legs. The detected stride and step number using the proposed method is excellent. For all young subjects, the accuracy of detecting stride number using proposed method is 100%. The accuracy of estimating travelled distance using proposed method is 97.73% for right and 98.82% for left legs.

Table 5 shows the details of both legs asymmetry variables information. The stride lengths of legs are the same for young subjects. The overall difference between legs is low for young subjects. In natural walking, the foot is on the ground for about 60% of the total gait cycle during stance phase and 40% during swing phase shown in Figure 9. The ratio of stance and swing is found closest to the 60:40% split for average stride, stance and swing information (Table 5) for young subjects.

Table 6 shows that the accuracy of estimating the total distance compared with the actual distance is high for both legs. For all older subjects the accuracy of detecting stride number using the proposed method is 92.67%. The accuracy of estimating the travelled distance using the proposed method is 88.71% for the right and 89.88% for the left legs. The detected stride and step number using the proposed method is also high. However, comparing to results in young subjects (Table 4), the accuracy is lower for older subjects. This is likely to be due to older people walking slowly resulting in a poorer signal output. Table 7 shows the details of both legs asymmetry variables for older subjects. Overall the stride lengths of both legs are similar. The overall difference between legs is very low.

We check the data for statistical errors and assessed whether the estimated values are reasonable. Figure 24 shows the boxplot of travelled distance from young and older subjects. It is noted that the observations identified by the boxplots are not especially extreme. The young subjects’ travelled distance for 30 strides has a wider range and is significantly different than older ones. On average young subjects travelled distance is 37.77 (95% CI ± 3.57) m and in older ones is 22.50 (95% CI ± 2.34) m. Similarly the legs stride and step variation is low for older ones than young ones. Older ones gait is slow and results in a low variation in walking comparing with young ones. The step length has more variation then stride length. Based on the total travelled distance, stride and step information, it can be seen that young and older subjects are distinguishable.

Figure 25 shows a boxplot of total time from young and older subjects with their difference. The total time for performing a total of 30 strides is lower for young subjects than older ones. On average the young subjects travelled time is 51.85 (95% CI ± 3.08) s and older ones is 84.02 (95% CI ± 9.98) s. Young subjects show low leg variation with a lower range than older ones. Based on the total time, stride and step timing information, it can be seen that young subjects and older ones are distinguishable.

The detailed results of the 20 subjects are presented in the Supporting Information.

### 3.3. Discussion

In this research we show that in a clinical setting outside of a gait laboratory it is possible to collect information about GA using IMU sensors. From Figure 10, we can see that our gait cycle accelerometer signal *AT_xyz_* is agreed with the signal pattern in [53,54]. We demonstrate the systematic steps of an automatic gait features extraction method that we deployed. Our research enriches the current literature in GA assessment. It is possible to evaluate walking distance using a multisensor approach. Current methods however rely on the threshold based detection of the spike [35,36,58,60]. Our method uses minimal prominence characteristics for detecting gait phases. The former relies on generating a movement of sufficient magnitude to generate the spike and therefore has limited utility in people with slow gait. Our method therefore has the potential for broader use as it can be used in people with slower gaits such as older adults. We demonstrate that our method can deliver accurate results of stride detection and distance travelled similar to accuracy levels demonstrated by other authors [36,60]. We believe that there are advantages to using the minimal prominence approach as it can be used in a wider population people with different gait patterns.

There are however a number of limitations. The number of subjects is still relatively small (20). There is the potential of a Type 1 error (false positive) in detecting an effect that is not there. IMU calibration is an essential part for distance estimation. Although in our methods we try to minimize errors, as gait features are intrinsically variable from person to person, any such algorithm should involve a degree of calibration and error in the measurements. Individual quirks, heel strike, significant body up-down movement and other factors can affect the results. However this can be considered a proof of concept study that has established our method for extracting automatic GA features. There are several other possible sources of errors [93] that may arise from the use of IMU sensors including errors of repeatability, stability and drift. Although IMU sensors performance has been ramped up dramatically, the errors in measurement are unavoidable, especially for miniature micro-electro-mechanical (MEMS) sensors. Future developments should focus on MEMS sensor error modelling and accommodation to further improve parameter estimation accuracy [94]. Other possible areas of error may arise from frictional noise and the relative movement of clothing and shoes to the sensor. However we compared the output from our sensor to a gold standard Qualisys motion capture system which shows good accuracy making the effect of such errors minimal.

To achieve our goal, data are collected from two sensors placed on the barefoot at the medial aspect of foot over the bony prominence of the first metatarsal. It is noted that the orientation of the sensor has a significant effect on output and placing the sensor in different locations gives a different pattern to the data. The position and orientation of the sensor are crucial as changes in position through human error may give different data patterns which might be difficult to interpret. This highlights the importance of properly fixing the sensor to the optimal location to avoid inaccuracies. The placing of sensors on foot locations requires other generic considerations such as battery life and android device that is BLE enabled to pick up sensor data. 

To estimate the orientation of the IMU sensors, we apply the Madgwick technique [51] for our collected data but not the magnetic field parameter. The technique is developed assuming that the acceleration would only measure gravity. In practice, accelerations due to motion will result in an erroneous observed direction of gravity and the distortion will present for only short periods of time. Therefore, the magnitude of the filter gain *β* (Section 2.6.3) is chosen low enough that the divergence caused by the erroneous gravitational observations is reduced to an acceptance level over the period. In future, an investigation of dynamic values of gains *β* and *ς* will be conducted to reduce errors.

A threshold is used for detecting steps [36,60] and different value may result in a different output. It is crucial to detect the 1st and last strides of gait cycle when a person starts and stop walking. Thus, the 1st stride is not considered by researchers [60]. Our proposed method for detecting the stride information is based on the local minimal prominence which starts when the heel contacts the ground resulting in the stationary period (Section 2.6.4) and estimated the total number of strides. We also confirm these results obtained by counting the highest peak in the mid-swing phase as it also is a good indicator for a complete gait cycle. From each stride, the local minimal prominence which is the transition between pre-swing and initial swing (4th and 5th phases in Figure 10) is detected. We find that that when turning or when stopping there is a poor acceleration signal. As gait of older subjects is much slower, it is crucial to detect strides, stance and swing phases from the gait cycle. However, the stationary stance phase is prominent for both young and older subjects. For this reason, we use the local minimal prominence characteristics to detect different events to avoid these crucial phases. We have shown that it is possible to detect, stride, stance and swing events but further analysis of the eight events including single and double support phases in a gait cycle is necessary to provide more accurate information for GA analysis. 

In order to track the position in a virtual environment, several navigation methods [63] are available to derive pose estimates from electrical measurements of mechanical, inertial, acoustic, magnetic, optical, and radio frequency sensors. Each approach has advantages and limitations including modality-specific limitations related to the physical medium, measurement-specific limitations imposed by the devices, associated signal-processing electronics, and circumstantial limitations that arise in a specific application [95]. Our velocity and distance estimation is based on results of a double integral with ZUPT. We apply the high pass filter on acceleration data that removes linear trend from the signal and then remove drift to estimate distance. We use the simplest technique of trapezoidal rule for estimating distance for our collected data and our estimated distance results are close to the actual distance. There are many other types of numerical integration schemes available which are much more involved and with the potential for more accuracy. However, the trapezoidal rule is the simplest technique of an entire class of numerical integration schemes which are known as the Newton-Cotes formulas [96] and which we have adopted. Our future plan is to investigate other methods with our collected data.

The results show that our method is capable of extracting automatic gait features and has the potential to be used in GA assessment and gait change monitoring for home and clinical use. Gait with slow velocity is common in older adults [97] and an automatic system sensitive enough to detect gait features in these circumstances is required. Our low cost portable personalized proposed solution could bring out automatic GA features for monitoring longitudinal gait changes or abnormalities. In future work, we plan to use our automatic extracted GA features information to classify gait changes over time to identify abnormal gait patterns for the assessment of elderly fall risk, rehabitation and sports applications. 

## 4. Conclusions

In the present work, two IMU sensors were placed at right and left metatarsal barefoot locations to collect accelerometer and gyroscope data. We designed and developed an android app to collect real time synchronous data from both sensors. We proposed a systematic method to extract automatic gait features for the GA assessment. We first applied the quaternion technique to raw data for estimating actual sensor orientation. We applied our proposed stride, stance, swing and step event detection technique and analysed for stride, step, cadence, step ratio, stance, and swing. We then estimated distance using double integration with drift removing from acceleration and analyzed for total velocity, distance and time. Our method was validated with the Qualisys motion capture system. We applied our method for 10 young and 10 older subjects. Our results show that it is possible to extract GA features automatically in a clinical setting outside of a gait laboratory. This has the potential to make the evaluation of GA widely available in clinical practice rather than being limited to gait laboratories.

## Figures and Tables

**Table 1:** Velocity, distance, stride and step information.

Older subject 1	Age	Height (m)	Weight (Kg)	Gender	
	67	1.57	68	Male	
Total Time (s)	99.3				
	Actual *	Right Leg **	Accuracy (%)	Left Leg **	Accuracy (%)
**Total Distance (m)**	21.03	20.59	97.91	20.47	97.34
**Estimated Velocity (m/s)**	0.21	0.21	97.91	0.21	97.34
**Detected Stride Number**	30	30	100	30	100
**Detected Step Number**	30	30	100	30	100

* ActualValue, ** EstimatedValue.

**Table 2:** Gait Asymmetry Variability.

	Right Leg					Left Leg				
Gait Features	Mean	Std	Var	Min	Max	Mean	Std	Var	Min	Max
**Stride Length (m)**	0.69	0.07	0.00	0.56	0.84	0.69	0.09	0.01	0.49	1.05
**Stride Time (s)**	2.80	0.28	0.08	2.30	3.40	2.85	0.37	0.13	2.05	4.30
**Stride Velocity (m/s)**	0.25					0.24				
**Step length (m)**	0.27	0.06	0.00	0.14	0.46	0.32	0.05	0.00	0.23	0.46
**Step time(s)**	1.28	0.30	0.09	0.65	2.15	1.52	0.26	0.07	1.10	2.15
**Step Velocity (m/s)**	0.25					0.24				
**Cadence (step/min)**	18.13					18.13				
**Step Ratio (Step length/cadence)**	0.02					0.02				
**Stance Time (s)**	1.65	0.23	0.05			1.67	0.35	0.12		
**Swing Length (m)**	0.69	0.35	0.12			0.69	0.44	0.19		
**Swing Time (s)**	1.16	0.23	0.05			1.18	0.21	0.04		
**Swing Velocity (m/s)**	0.59					0.58				

Std = Standard Deviation, Var = Variance, Min = Minimum and Max = Maximum.

**Table 3:** Validation our results with Qualisys.

Participants	Leg	Qualisys	Estimated		Qualisys	Period	
		Distance (m)		Accuracy (%)	Time (s)		Accuracy (%)
**1**	**Right**	7.650	7.607	99.435	12.670	12.510	98.740
	**Left**	7.522	7.459	99.159	12.330	12.330	100.000
**2**	**Right**	7.402	7.155	96.664	12.670	12.830	98.740
	**Left**	7.456	7.327	98.270	12.330	12.280	99.590
**3**	**Right**	8.181	8.126	99.330	8.720	8.740	99.770
	**Left**	7.984	7.747	97.034	8.280	8.150	98.430
**4**	**Right**	7.978	7.806	97.848	8.720	8.880	98.170
	**Left**	8.121	8.061	99.259	8.280	8.180	98.790
**5**	**Right**	7.735	7.699	99.531	9.780	9.750	99.690
	**Left**	7.842	7.791	99.345	9.720	9.640	99.180
**6**	**Right**	7.564	7.493	99.066	9.780	9.710	99.280
	**Left**	7.481	7.518	99.505	9.720	9.830	98.870
**7**	**Right**	7.693	6.784	88.181	7.380	7.250	98.240
	**Left**	7.626	7.197	94.377	7.030	7.130	98.580
**8**	**Right**	7.422	6.939	93.497	7.380	7.310	99.050
	**Left**	7.144	6.669	93.344	7.030	7.140	98.440
**9**	**Right**	7.769	7.744	99.678	7.940	7.910	99.620
	**Left**	7.755	7.626	98.331	7.870	8.000	98.350
**10**	**Right**	7.508	7.485	99.698	7.940	7.960	99.750
	**Left**	7.623	7.613	99.870	7.870	7.800	99.110

Estimated = Estimated distance using our method; Period = Total time of travelling the distance.

**Table 4:** Velocity, distance, stride and step results for young subjects.

AVERAGE	Age	Height (m)	Weight (Kg)	Gender	
	25.30	1.61	61.90	9 M, 1 F	
Total Time (s)	51.85				
	Actual *	Right Leg **	Accuracy	Left Leg **	Accuracy
**Total Distance (m)**	37.77	37.19	97.73	37.81	98.82
**Estimated Velocity (m/s)**	0.73	0.72	97.73	0.73	98.82
**Detected Stride Number**	30.00	30.00	100.00	30.00	100.00
**Detected Step Number**	30.00	30.00	100.00	30.00	100.00

* ActualValue, ** EstimatedValue.

**Table 5:** Right and left legs asymmetry of young subjects.

	Right					Left				
Gait Features	Mean	Std	Var	Min	Max	Mean	Std	Var	Min	Max
**Stride Length (m)**	1.17	0.17	0.03	0.91	1.65	1.17	0.17	0.03	0.89	1.61
**Stride Time (s)**	1.41	0.20	0.04	1.10	1.97	1.39	0.19	0.04	1.07	1.91
**Stride Velocity (m/s)**	0.83	0.83	0.83			0.84	0.84	0.84		
**Cadence (step/min)**	34.93					34.93				
**Step Velocity (m/s)**	0.83	0.85	0.75			0.84	0.86	0.77		
**Step length (m)**	0.50	0.14	0.02	0.15	0.84	0.54	0.18	0.04	0.24	1.10
**Step time(s)**	0.68	0.19	0.04	0.21	1.14	0.74	0.24	0.07	0.33	1.48
**Step Ratio (Step length/cadence)**	0.01					0.01				
**Stance Time (s)**	0.84	0.16	0.03			0.83	0.13	0.02		
**Swing Length (m)**	1.17	0.68	0.49			1.17	0.94	1.14		
**Swing Time (s)**	0.58	0.12	0.02			0.57	0.15	0.02		
**Swing Velocity (m/s)**	2.07	1.80	1.80			2.10	1.83	1.83		

**Table 6:** Velocity, distance, stride and step results for older subjects.

Average	Age	Height (m)	Weight (Kg)	Gender	
	69.40	1.52	63.40	9 M, 1F	
Total Time (s)	80.26				
	Actual *	Right Leg **	Accuracy	Left Leg **	Accuracy
**Total Distance (m)**	22.49	22.21	88.71	21.19	89.88
**Estimated Velocity (m/s)**	0.31	0.29	88.71	0.27	89.88
**Detected Stride Number**	30	27.80	92.67	27.80	92.67
**Detected Step Number**	30	27.80	92.67	27.80	92.67

* ActualValue, ** EstimatedValue.

**Table 7:** Right and left legs asymmetry of older subjects.

	Right					Left				
Gait Features	Mean	Std	Var	Min	Max	Mean	Std	Var	Min	Max
**Stride Length (m)**	0.74	0.14	0.02	0.54	1.16	0.74	0.13	0.02	0.55	1.09
**Stride Time (s)**	2.47	0.46	0.24	1.84	3.88	2.44	0.39	0.16	1.80	3.47
**Stride Velocity (m/s)**	0.32	0.32	0.32			0.33	0.32	0.32		
**Cadence (step/min)**	24.34					24.34				
**Step Velocity (m/s)**	0.32	0.33	0.13			0.33	0.33	0.13		
**Step length (m)**	0.22	0.23	0.06	0.22	0.63	0.35	0.18	0.04	0.04	0.57
**Step time(s)**	0.54	0.86	1.02	1.02	2.07	1.13	0.73	0.81	0.18	2.20
**Step Ratio (Step length/cadence)**	0.007					0.014				
**Stance Time (s)**	1.39	0.30	0.10			1.37	0.28	0.08		
**Swing Length (m)**	0.74	0.78	0.73			0.74	0.76	0.66		
**Swing Time (s)**	1.09	0.30	0.10			1.07	0.23	0.05		
**Swing Velocity (m/s)**	0.78	0.68	0.68			0.78	0.65	0.65		

## Supplementary Materials

The supporting information is available online at http://www.mdpi.com/1424-8220/18/2/676/s1.
