# Supplementary material for: An Automatic Gait Feature Extraction Method for Identifying Gait Asymmetry Using Wearable Sensors
Source: Sensors (Basel). 2018 Feb 24;18(2):676. doi: 10.3390/s18020676 (PMC5855014; doi:10.3390/s18020676)
Supplement: Supplementary file 1 [file sensors-18-00676-s001.pdf]

# Appendix A

1: Young Participant 1

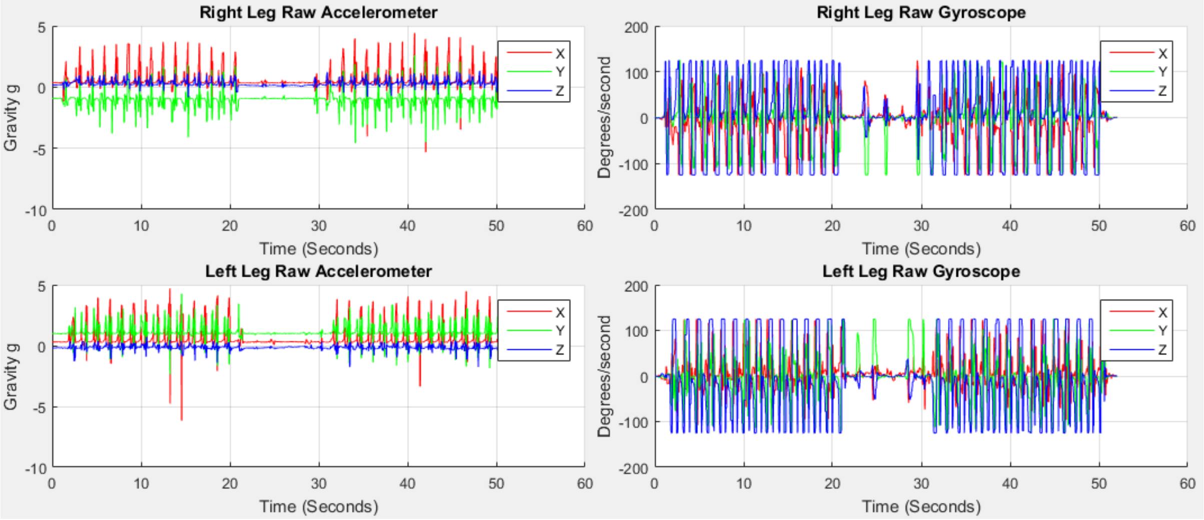


Figure 1.1: Accelerometer and gyroscope data from right and left legs

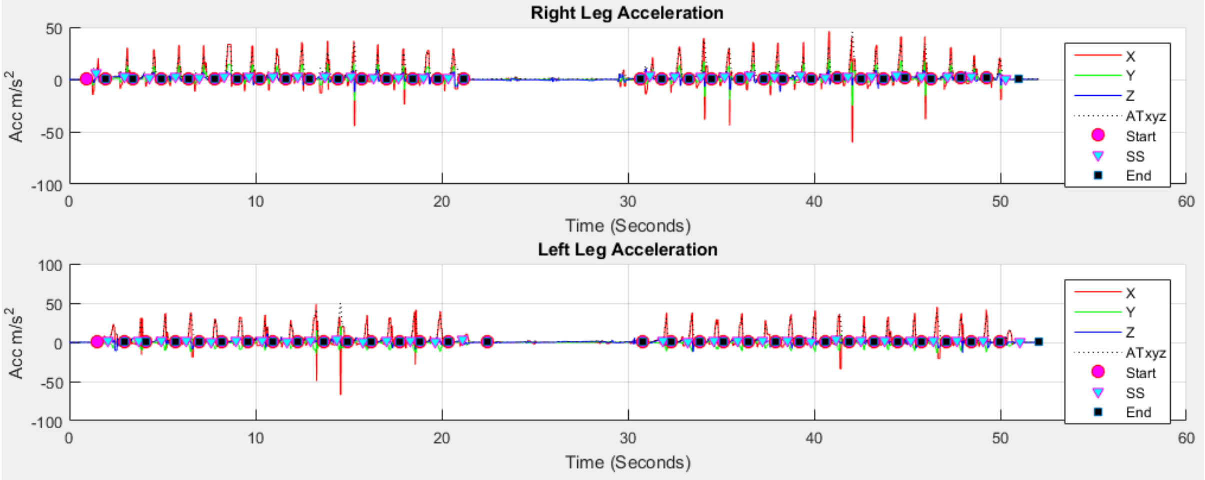


Figure 1.2: Result of stride, stance and swing event detection using proposed method

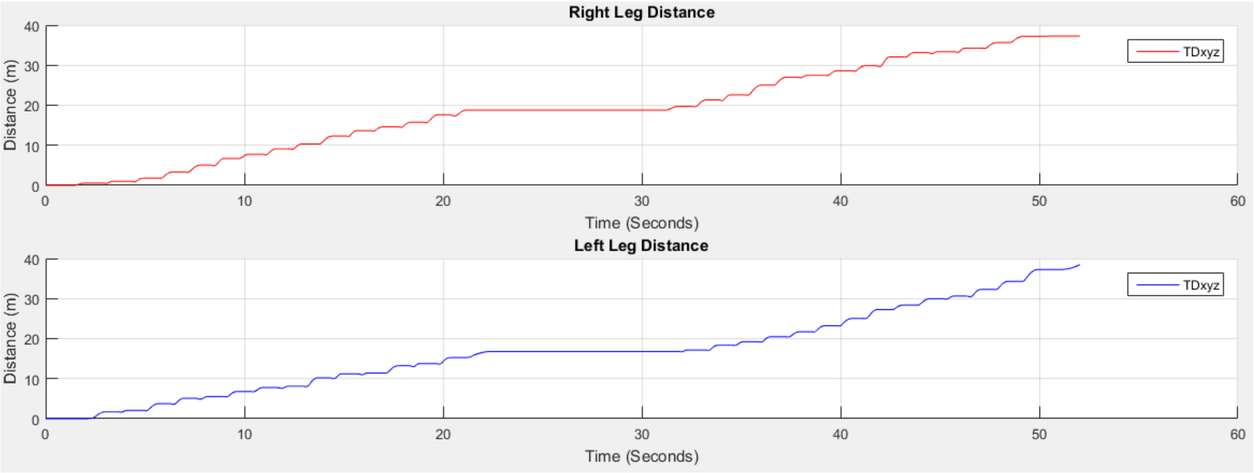

Figure 1.3: Result of distance estimation using proposed method

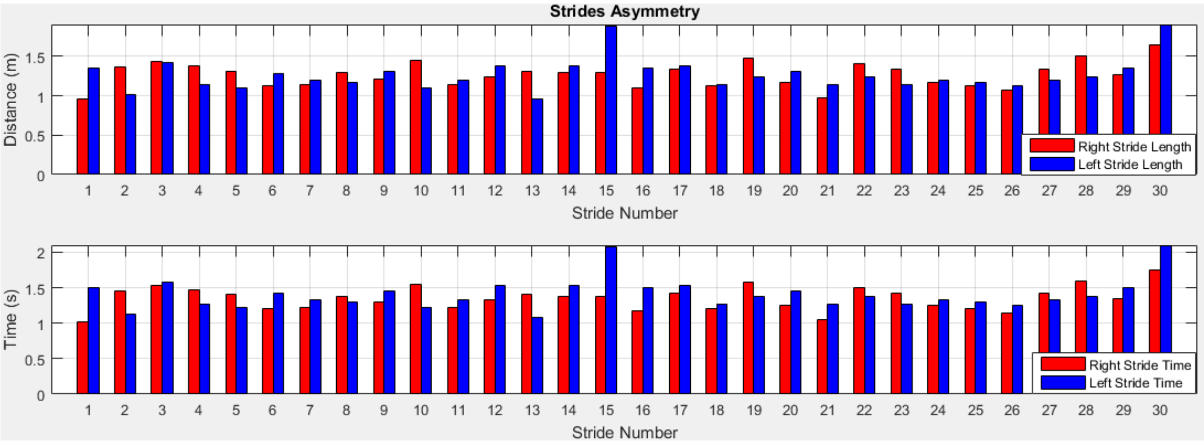

Figure 1.4: Stride asymmetry estimation of right and left legs

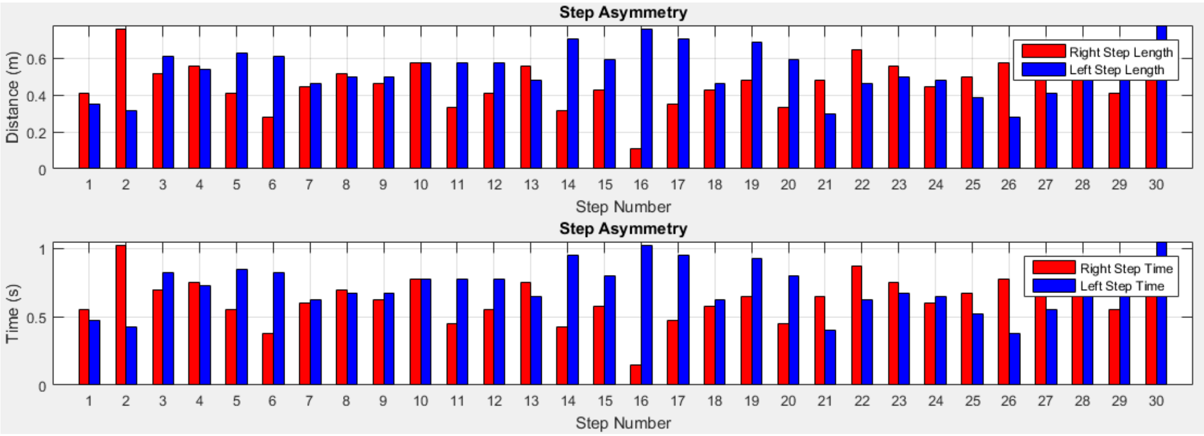

Figure 1.5: Step asymmetry estimation of right and left legs

## 2: Young Participant 2

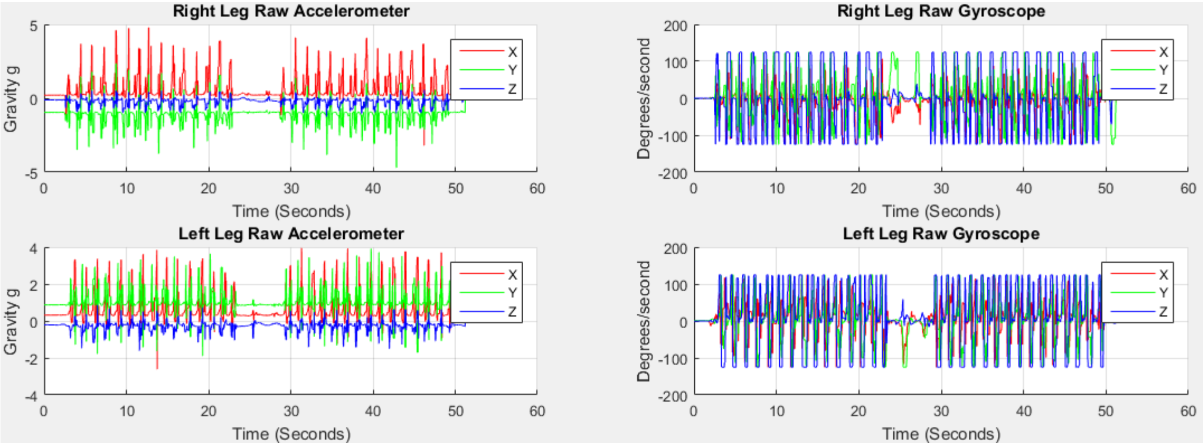

Figure 2.1: Accelerometer and gyroscope data from right and left legs

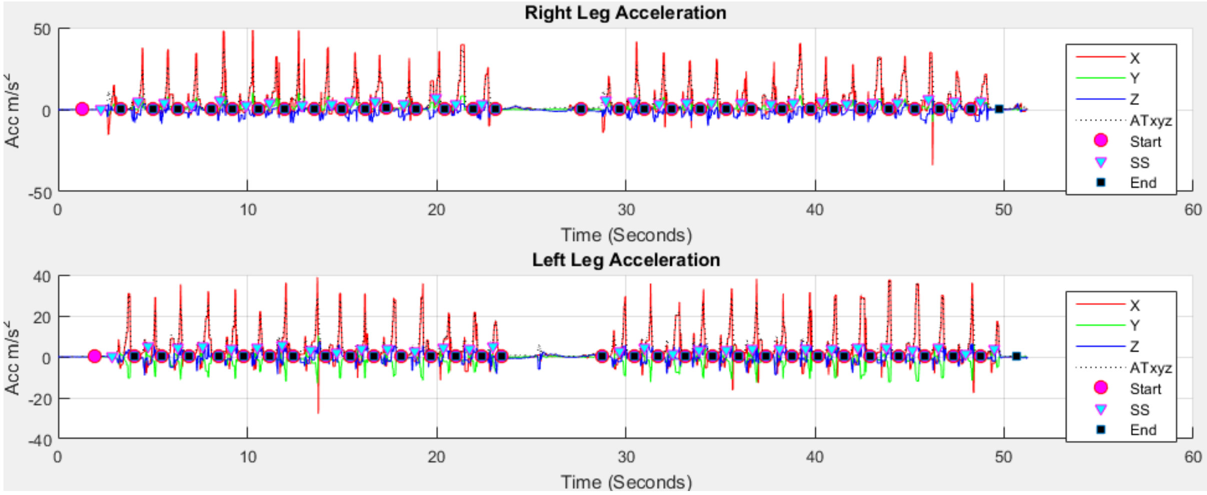

Figure 2.2:Result of stride, stance and swing event detection using proposed method

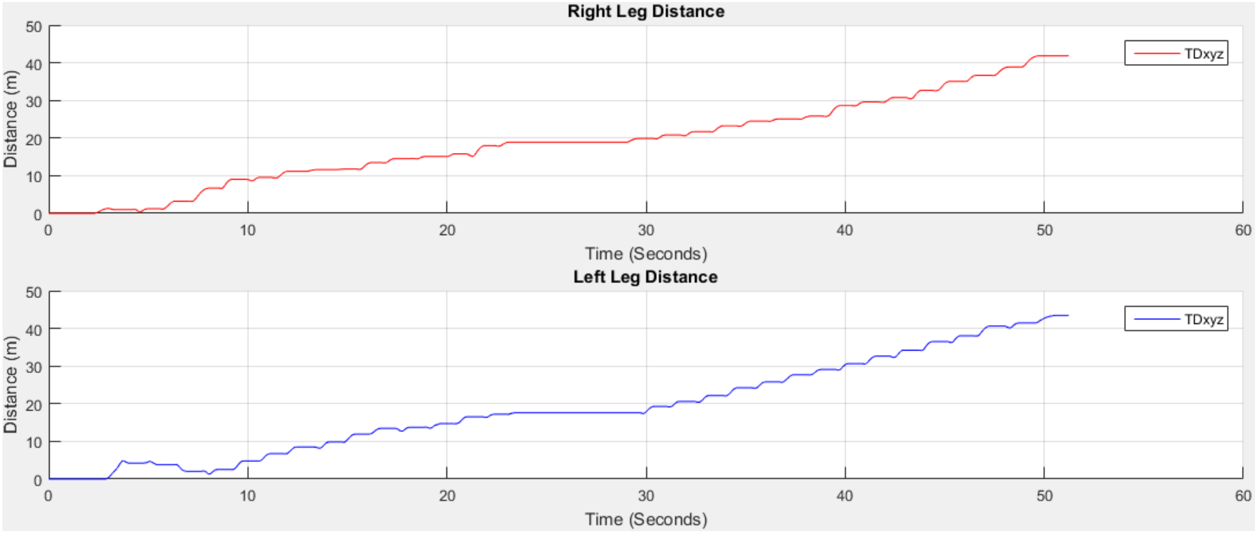


Figure 2.3: Result of distance estimation using proposed method

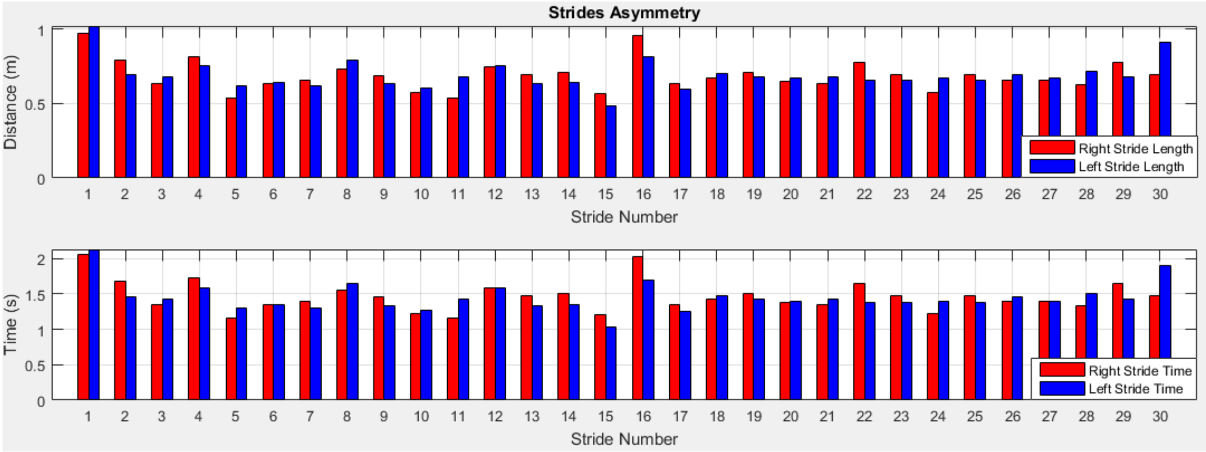


Figure 2.4:Stride asymmetry estimation of right and left legs

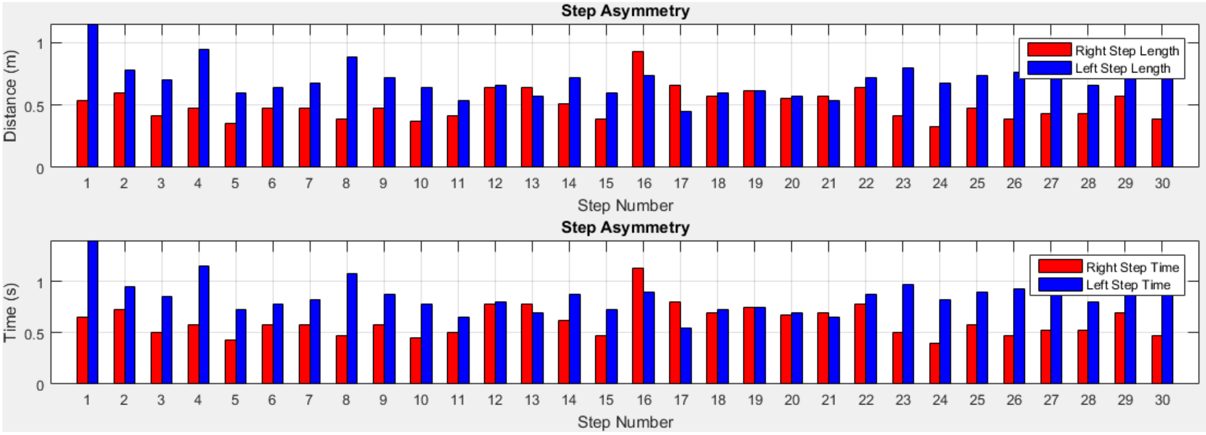


Figure 2.5: Step asymmetry estimation of right and left legs

3: Young Participant 3

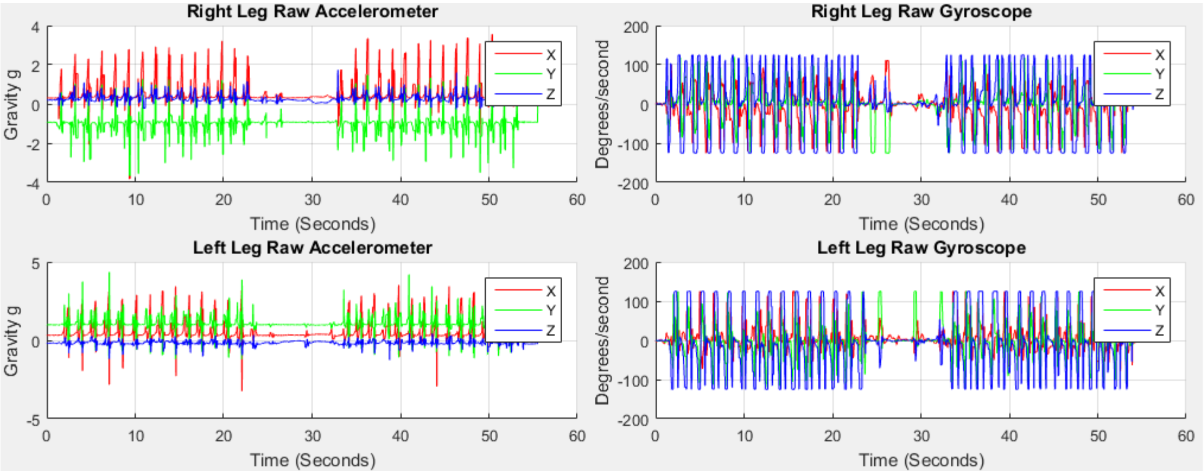


Figure 3.1: Accelerometer and gyroscope data from right and left legs

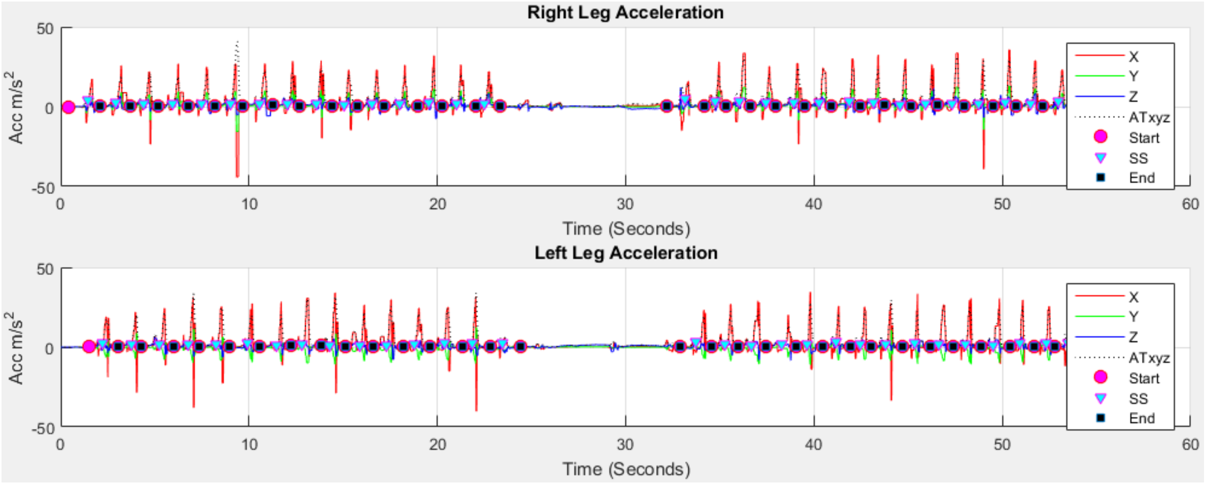


Figure 3.2: Result of stride, stance and swing event detection using proposed method

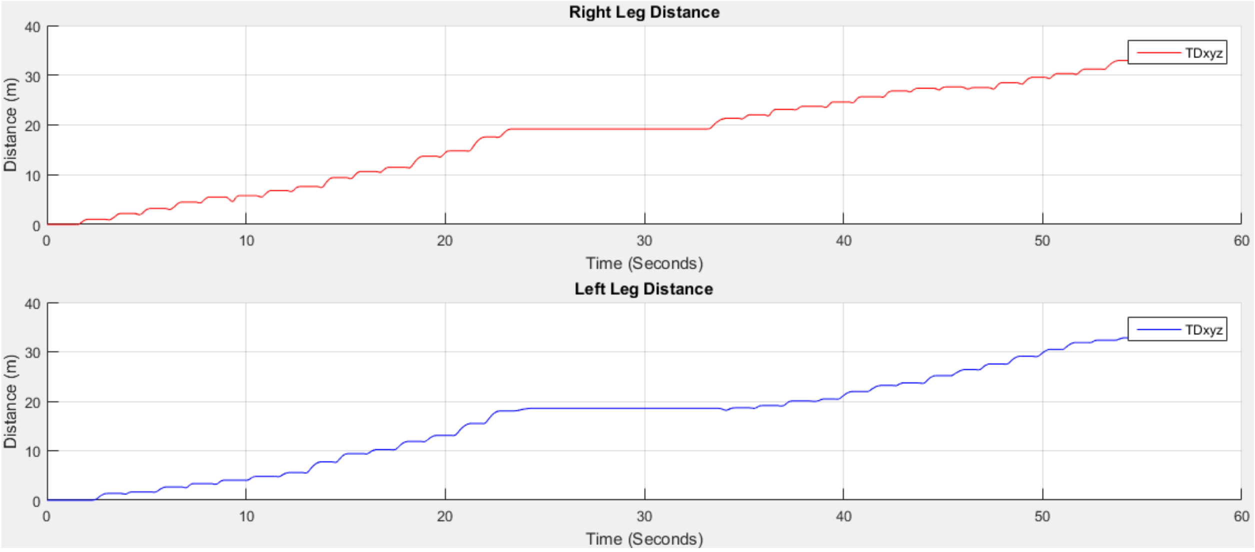


Figure 3.3: Result of distance estimation using proposed method

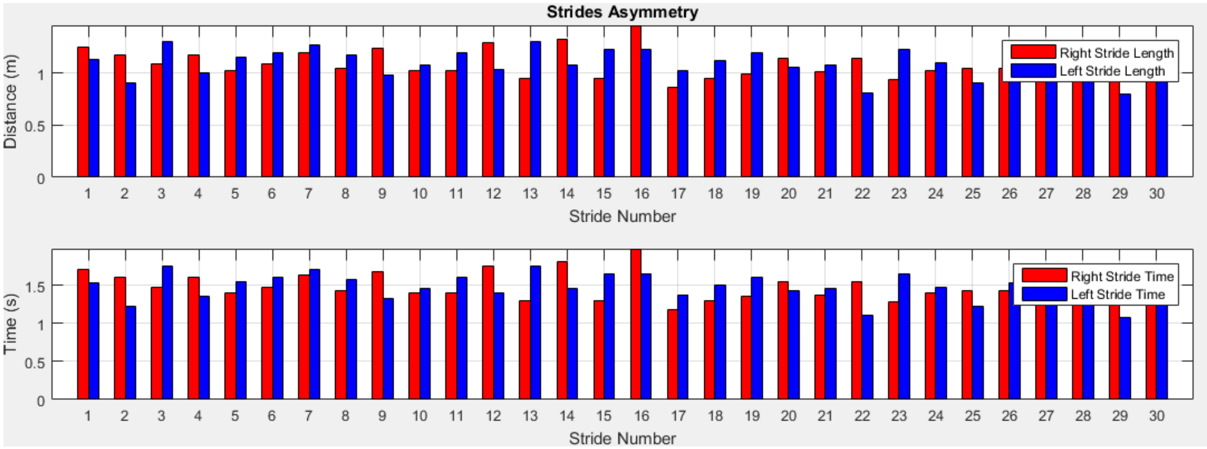

Figure 3.4: Stride asymmetry estimation of right and left legs

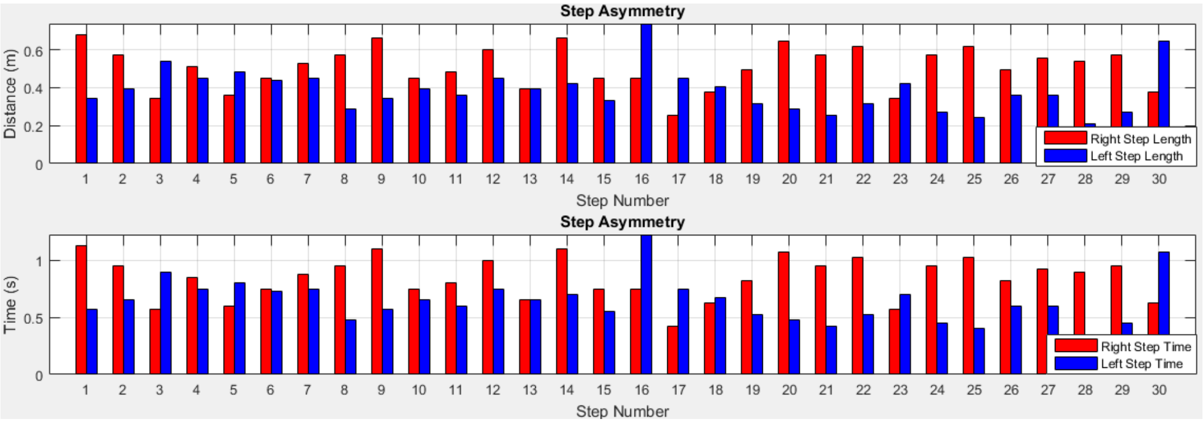


Figure 3.5: Step asymmetry estimation of right and left legs

4: Young Participant 4

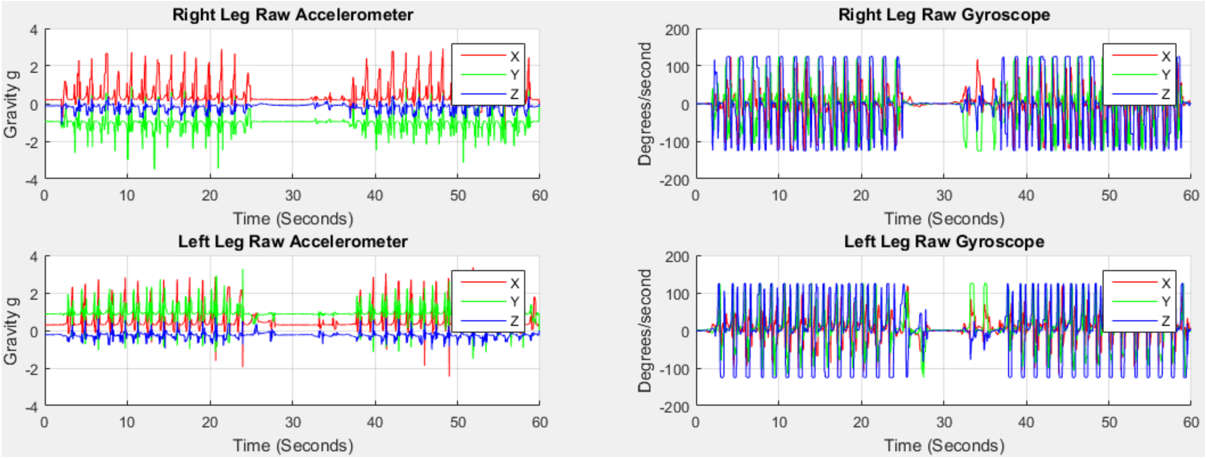


Figure 4.1: Accelerometer and gyroscope data from right and left legs

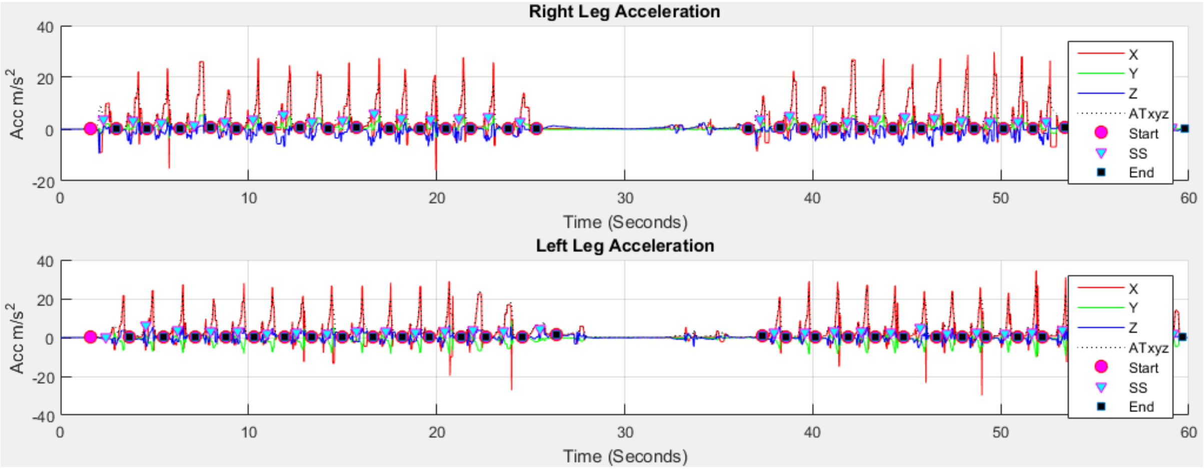


Figure 4.2: Result of stride, stance and swing event detection using proposed method

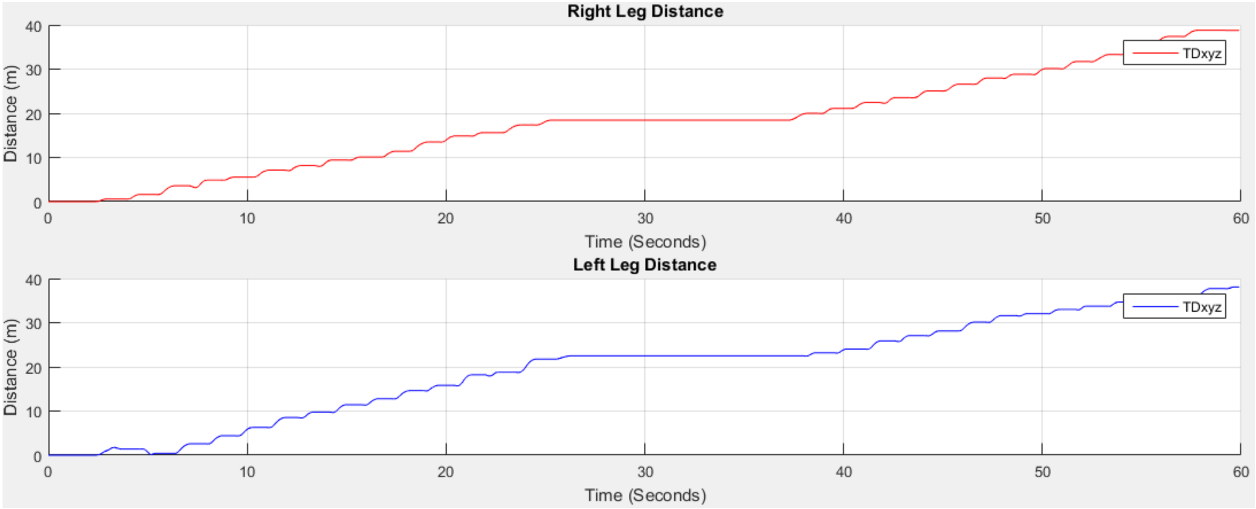


Figure 4.3: Result of distance estimation using proposed method

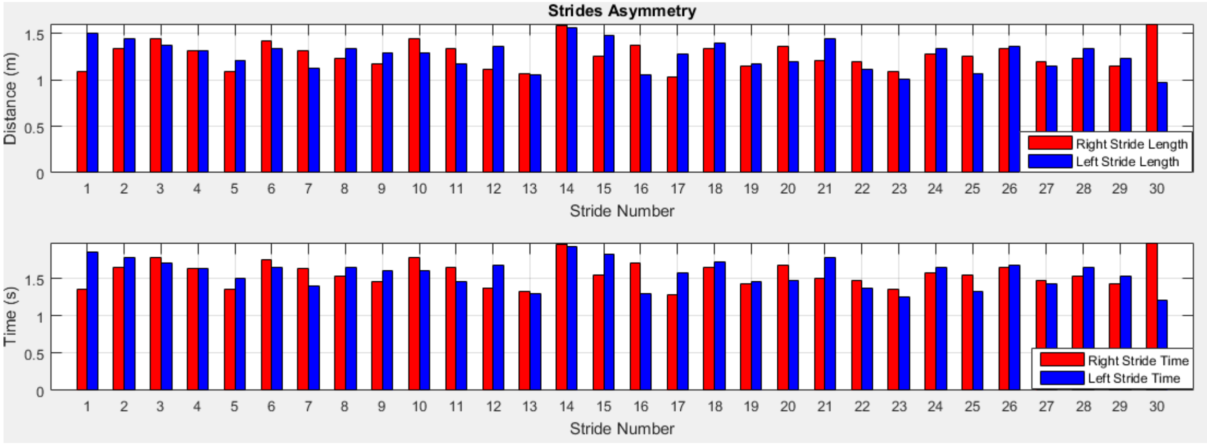


Figure 4.4: Stride asymmetry estimation of right and left legs

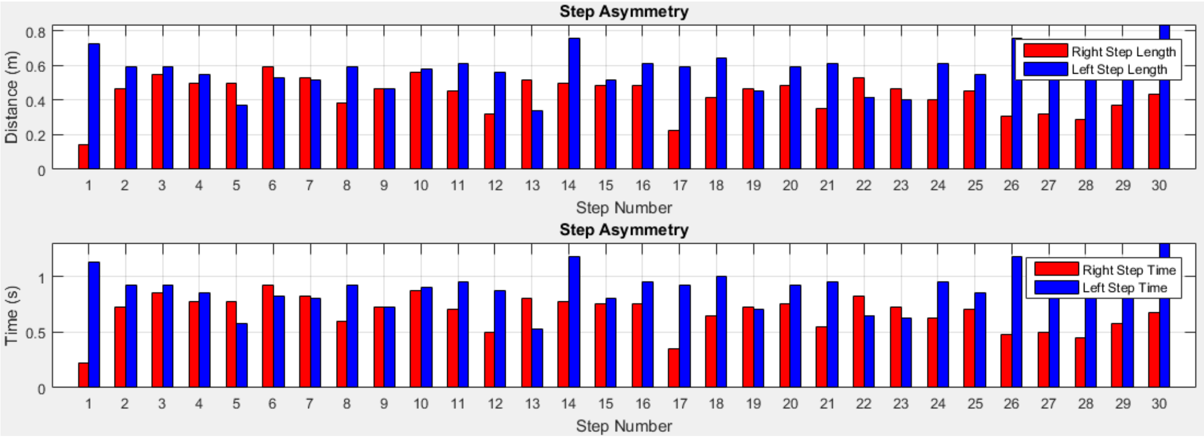


Figure 4.5: Step asymmetry estimation of right and left legs

5: Young Participant 5

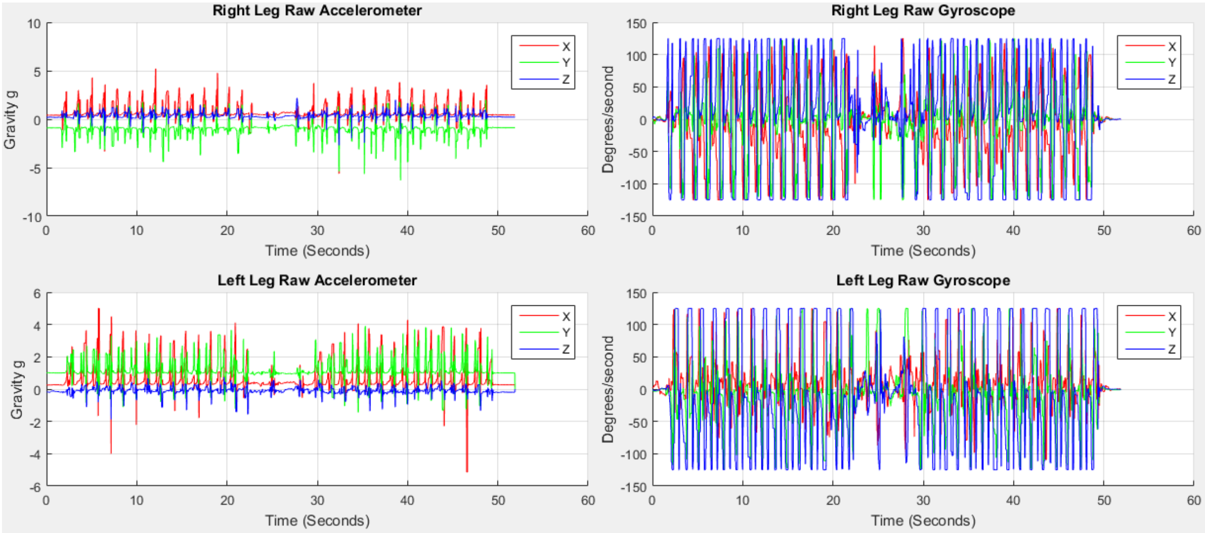


Figure 5.1: Accelerometer and gyroscope data from right and left legs

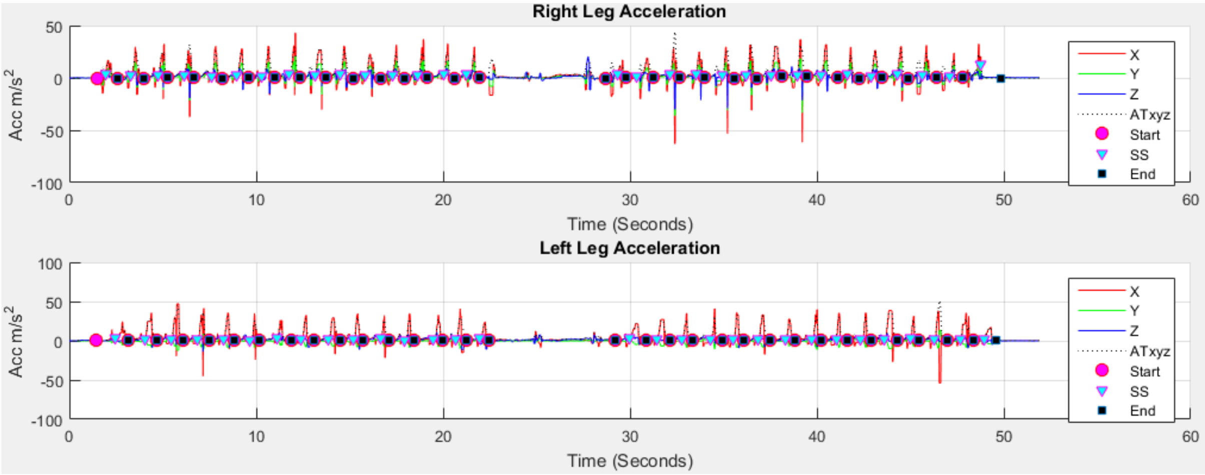


Figure 5.2: Result of stride, stance and swing event detection using proposed method

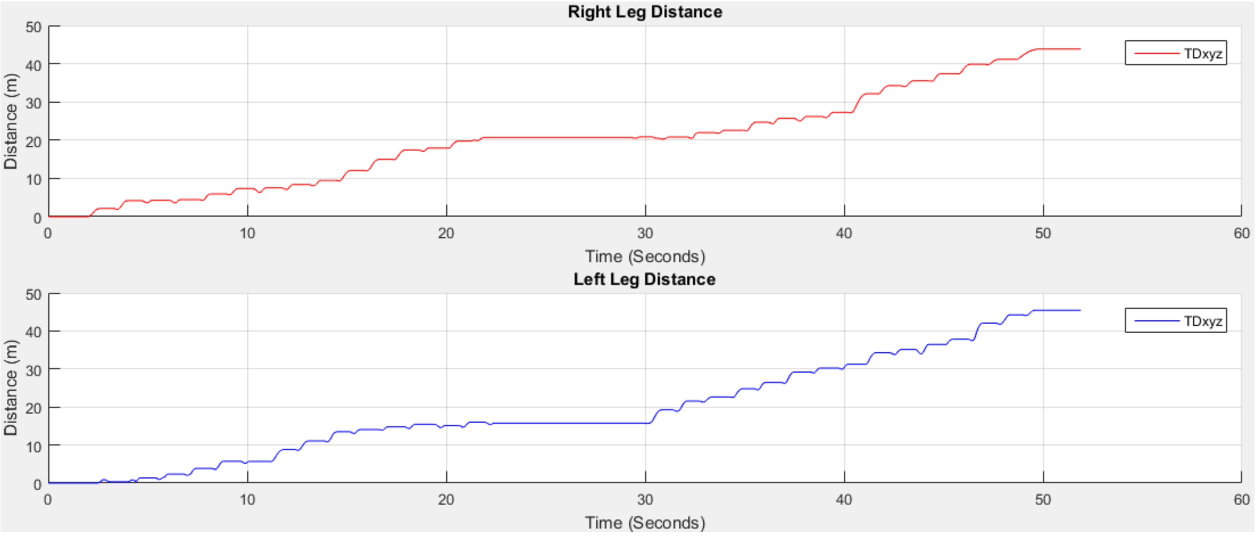


Figure 5.3: Result of distance estimation using proposed method

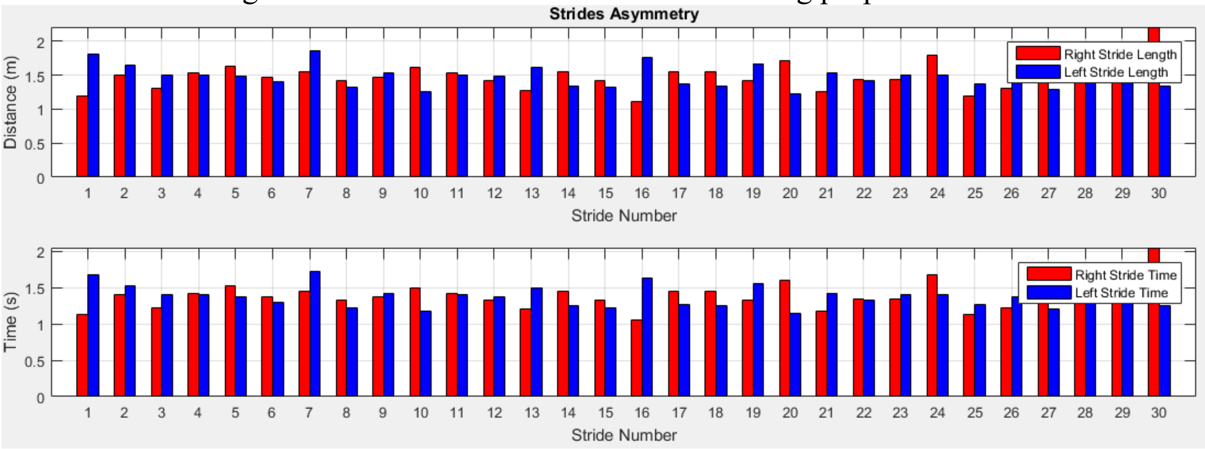


Figure 5.4: Stride asymmetry estimation of right and left legs

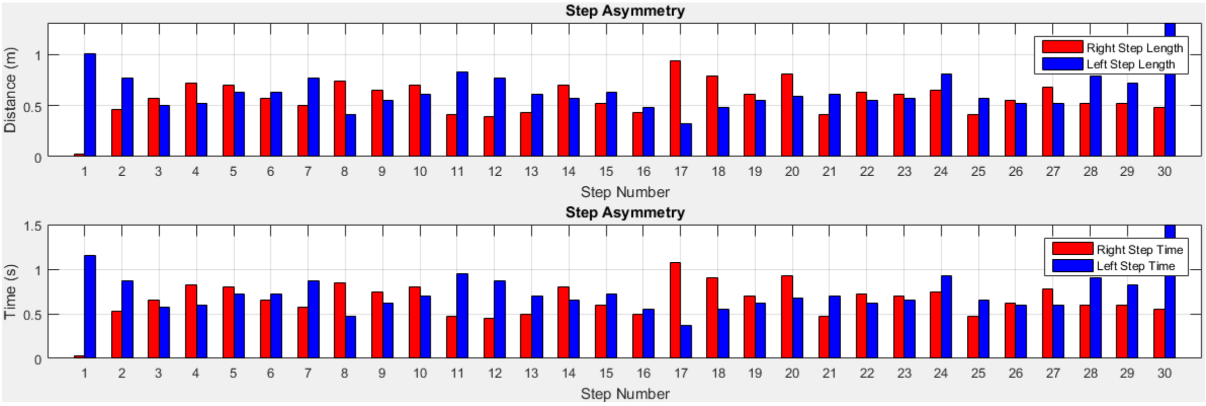


Figure 5.5: Step asymmetry estimation of right and left legs

6: Young Participant 6

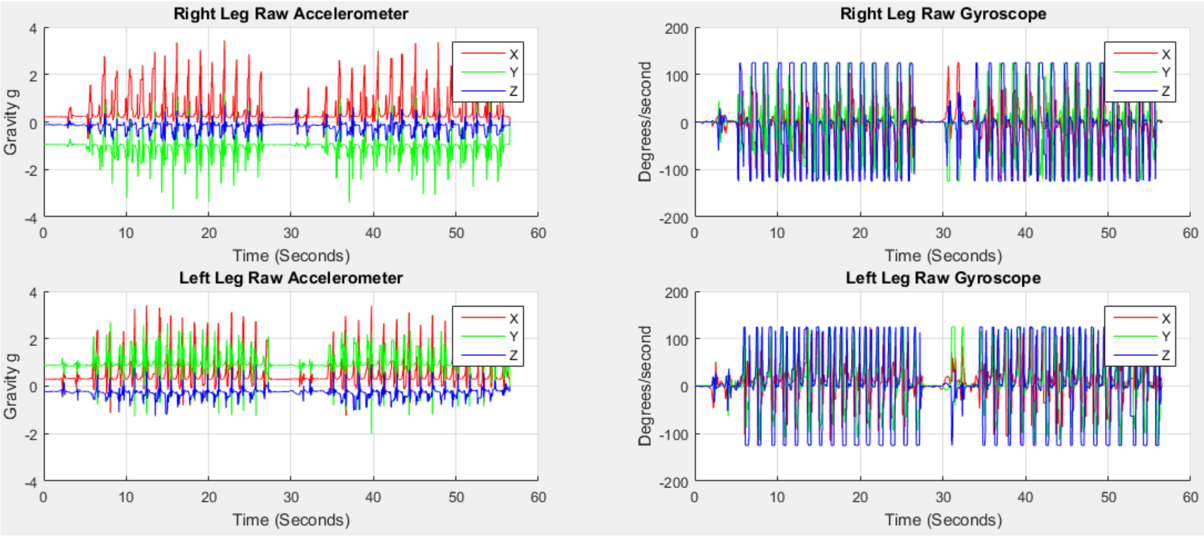


Figure 6.1: Accelerometer and gyroscope data from right and left legs

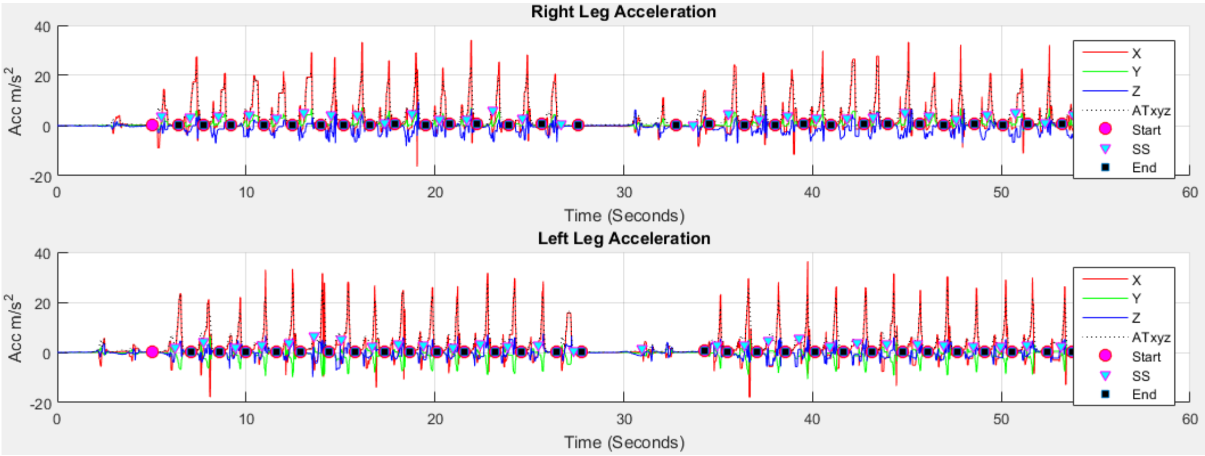


Figure 6.2: Result of stride, stance and swing event detection using proposed method

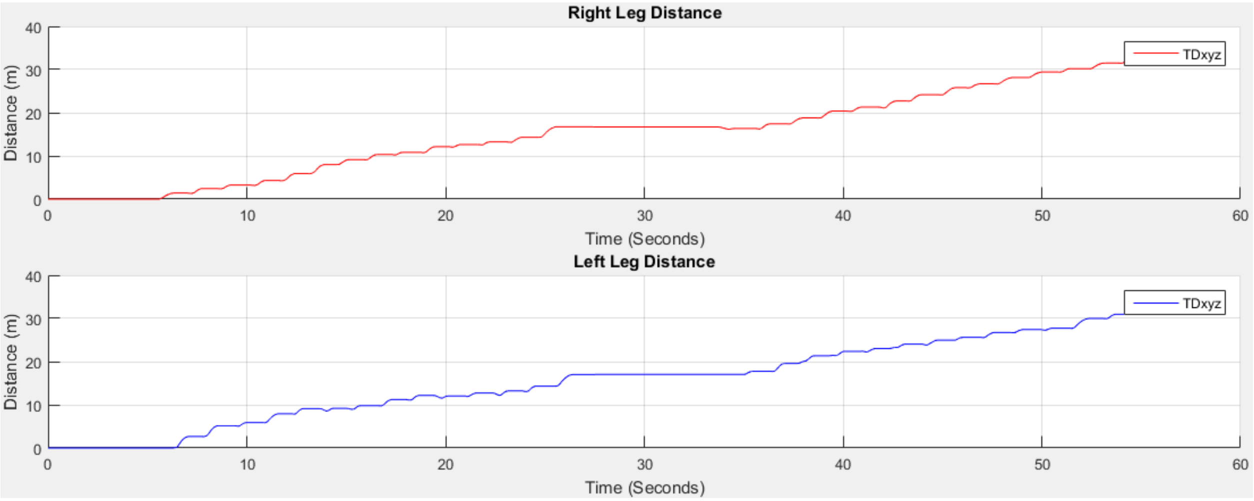


Figure 6.3: Result of distance estimation using proposed method

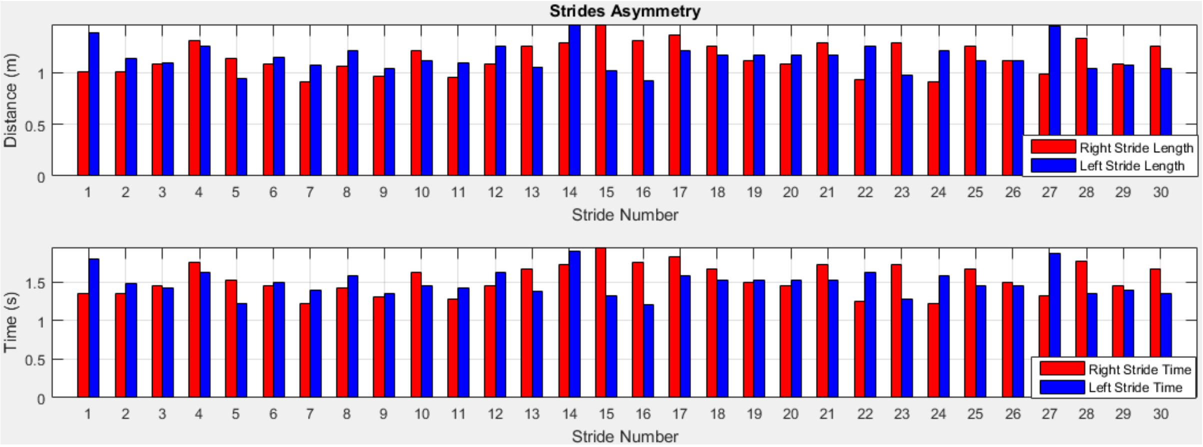


Figure 6.4: Stride asymmetry estimation of right and left legs

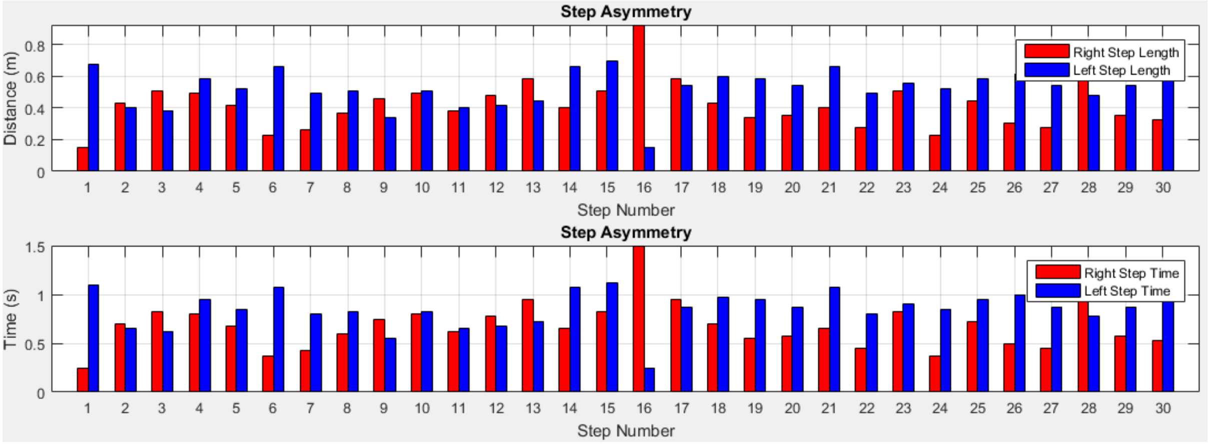


Figure 6.5: Step asymmetry estimation of right and left legs

7: Young Participant 7

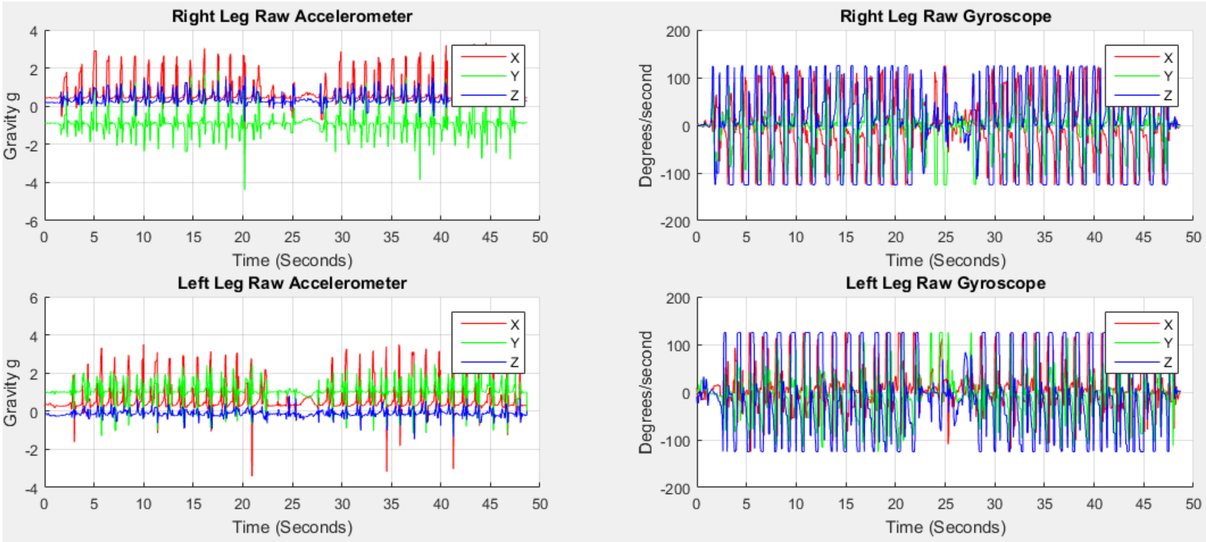


Figure 7.1: Accelerometer and gyroscope data from right and left legs

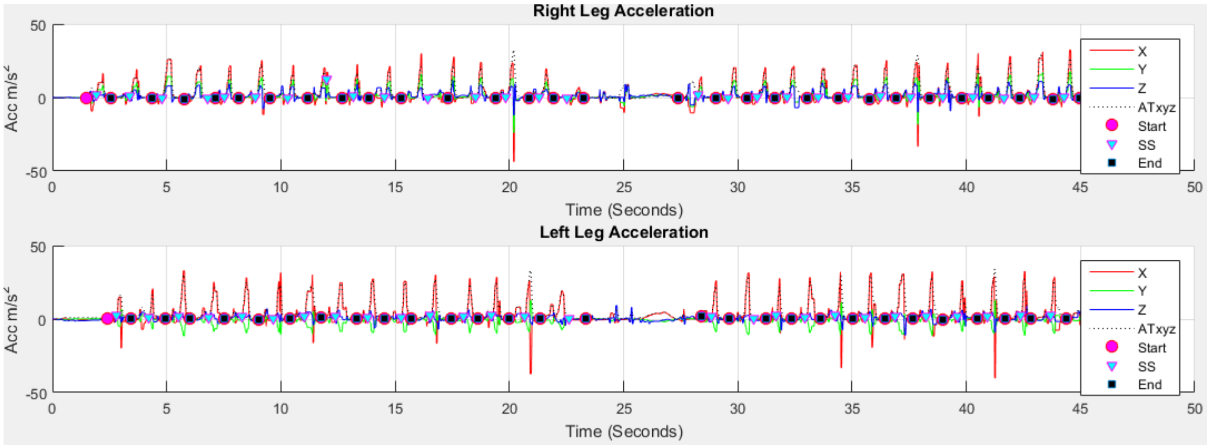


Figure 7.2: Result of stride, stance and swing event detection using proposed method

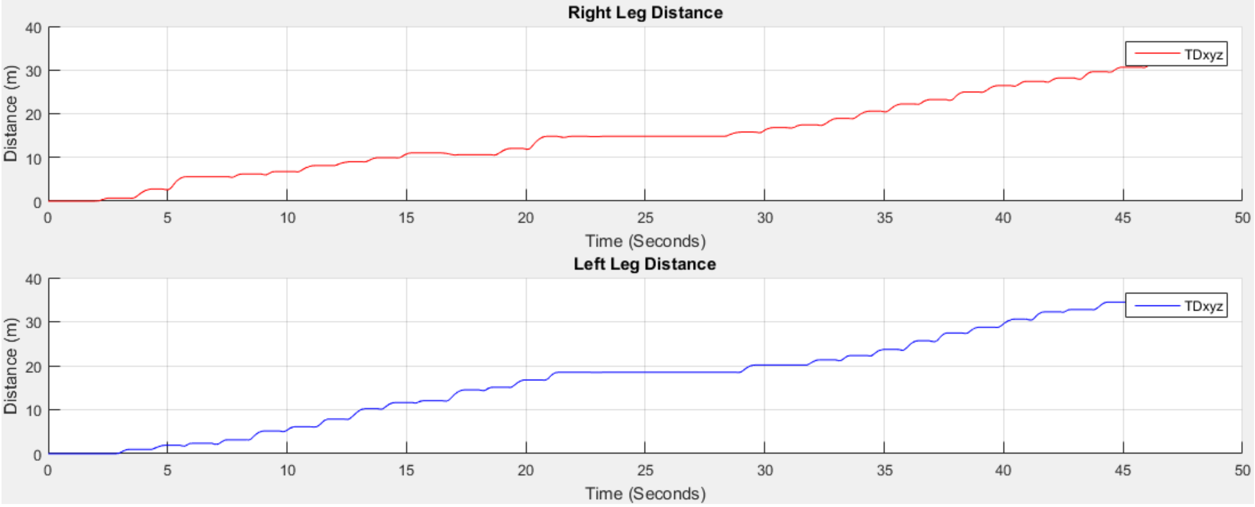


Figure 7.3: Result of distance estimation using proposed method

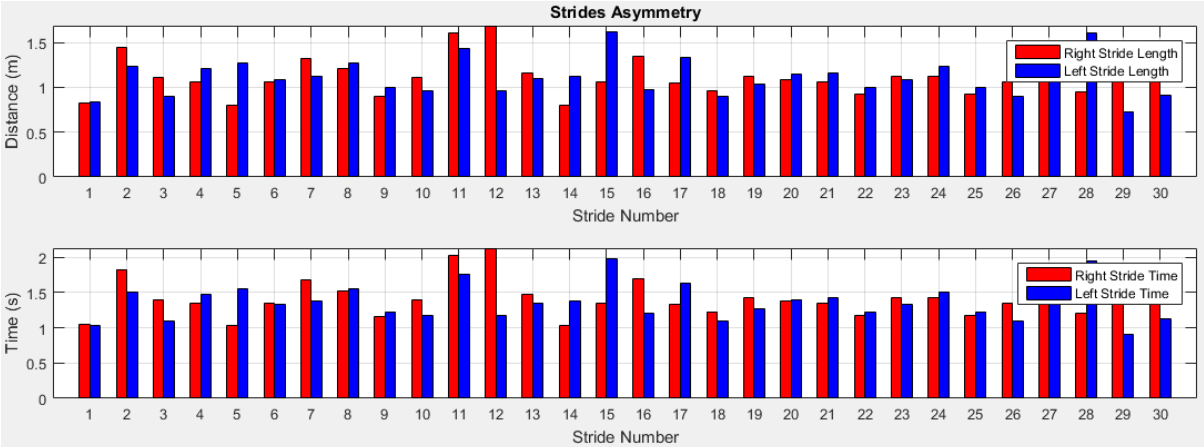


Figure 7.4: Stride asymmetry estimation of right and left legs

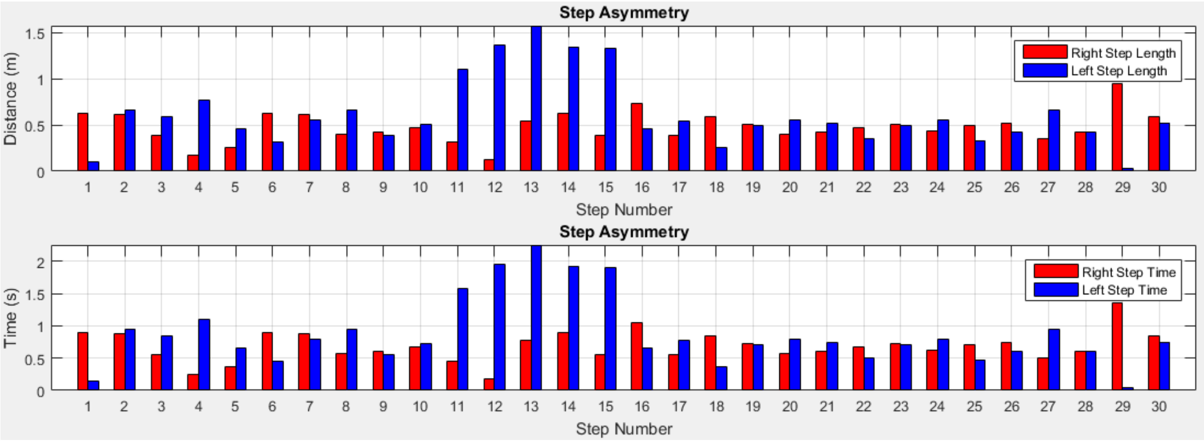


Figure 7.5: Step asymmetry estimation of right and left legs

## 8: Young Participant 8

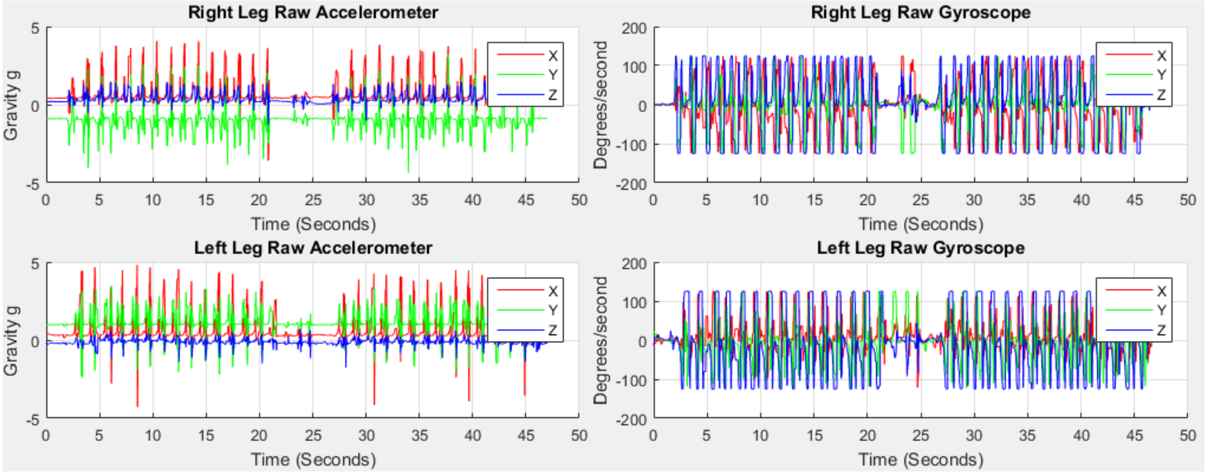


Figure 8.1: Accelerometer and gyroscope data from right and left legs

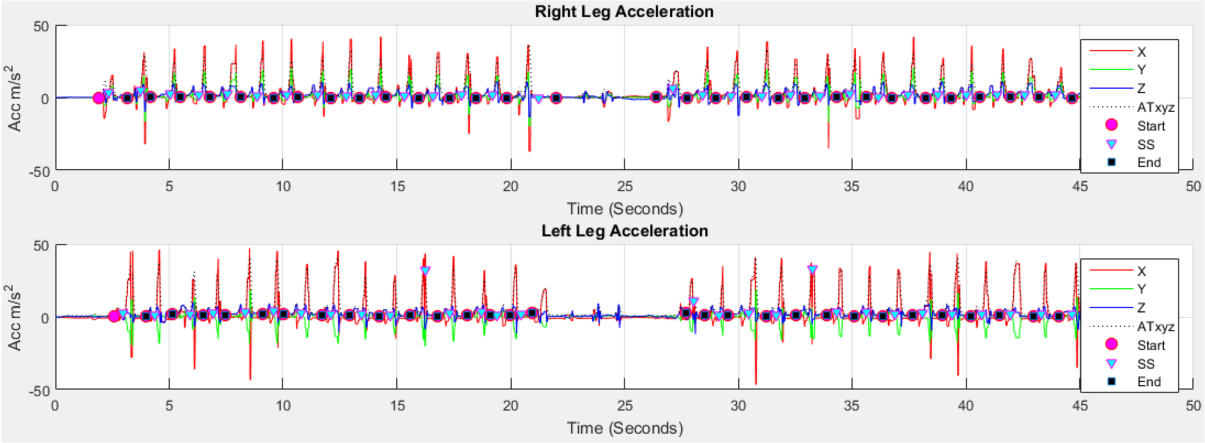


Figure 8.2: Result of stride, stance and swing event detection using proposed method

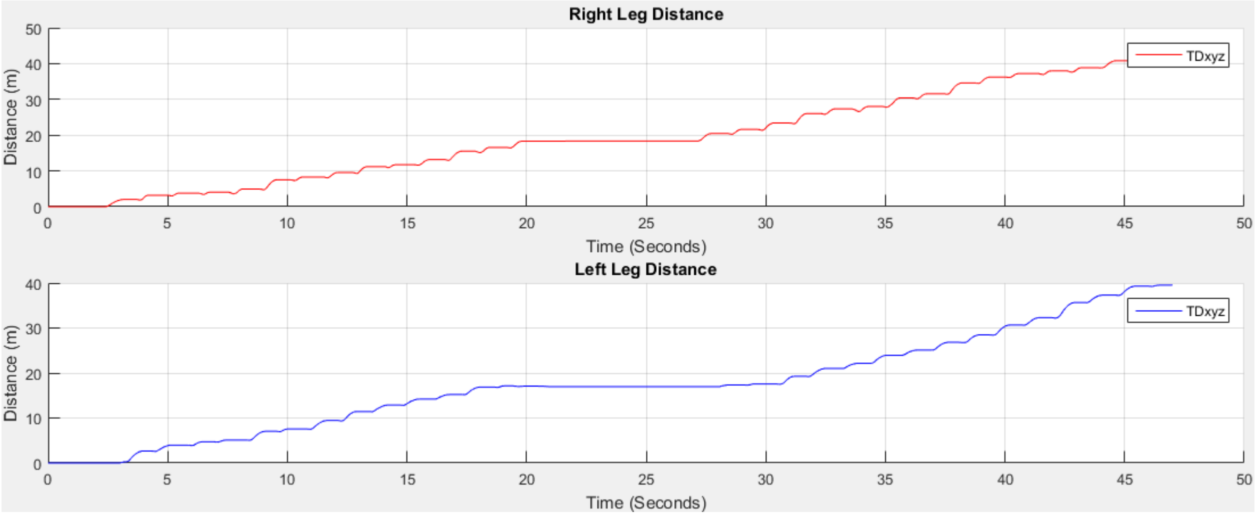


Figure 8.3: Result of distance estimation using proposed method

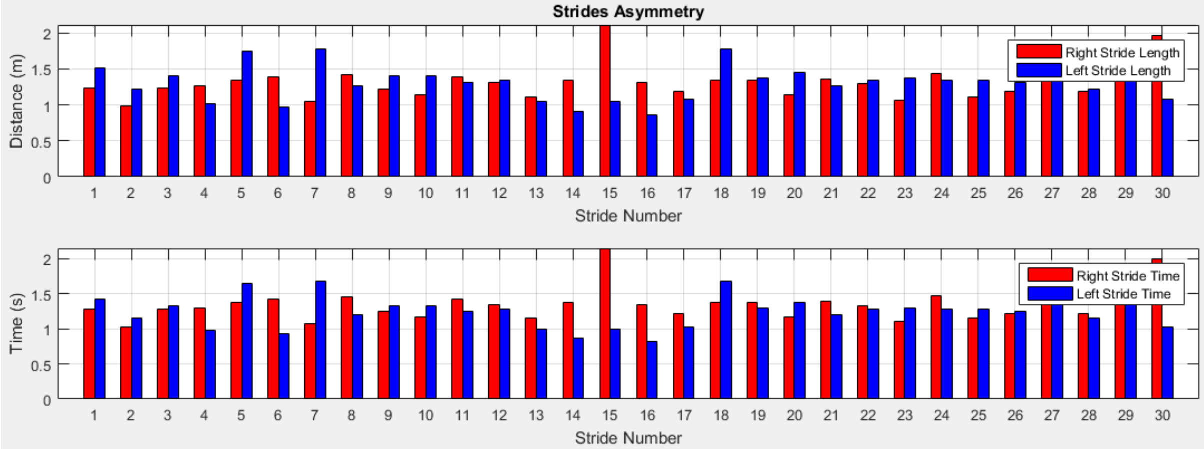


Figure 8.4: Stride asymmetry estimation of right and left legs

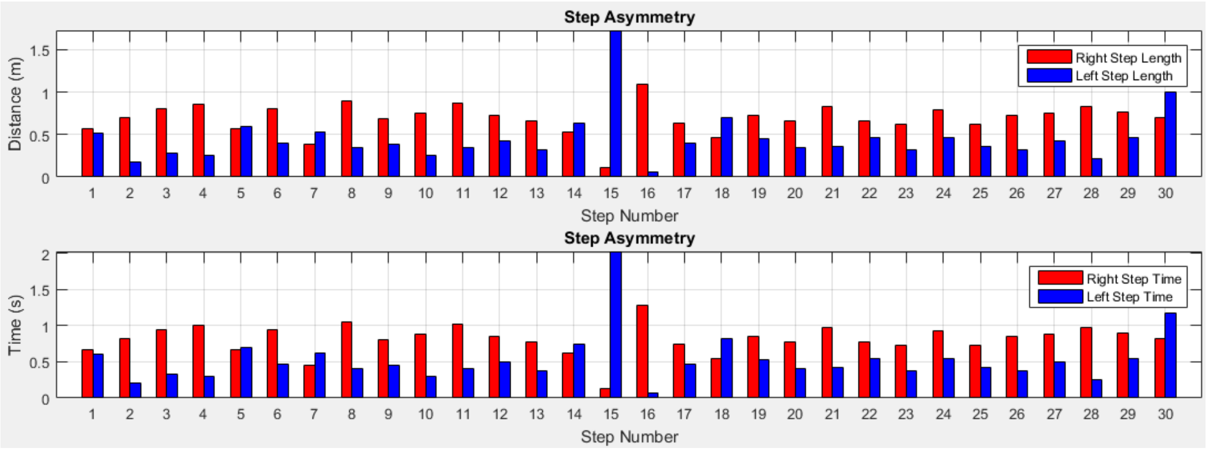


Figure 8.5: Step asymmetry estimation of right and left legs

9: Young Participant 9

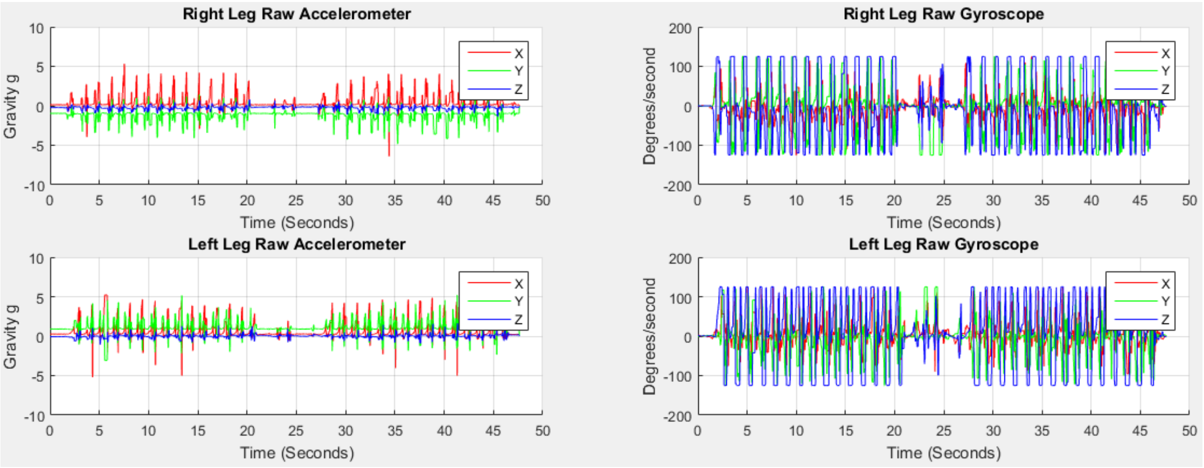


Figure 9.1: Accelerometer and gyroscope data from right and left legs

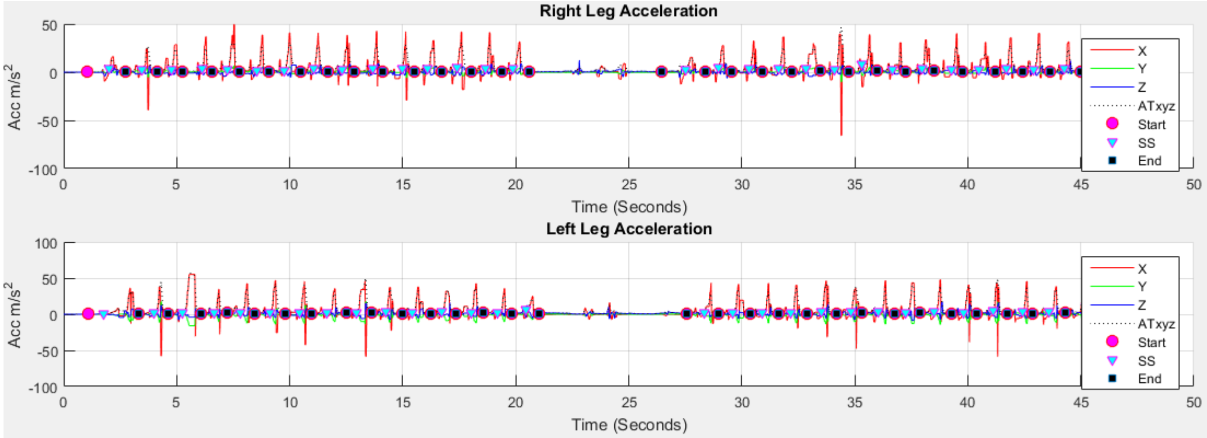


Figure 9.2: Result of stride, stance and swing event detection using proposed method

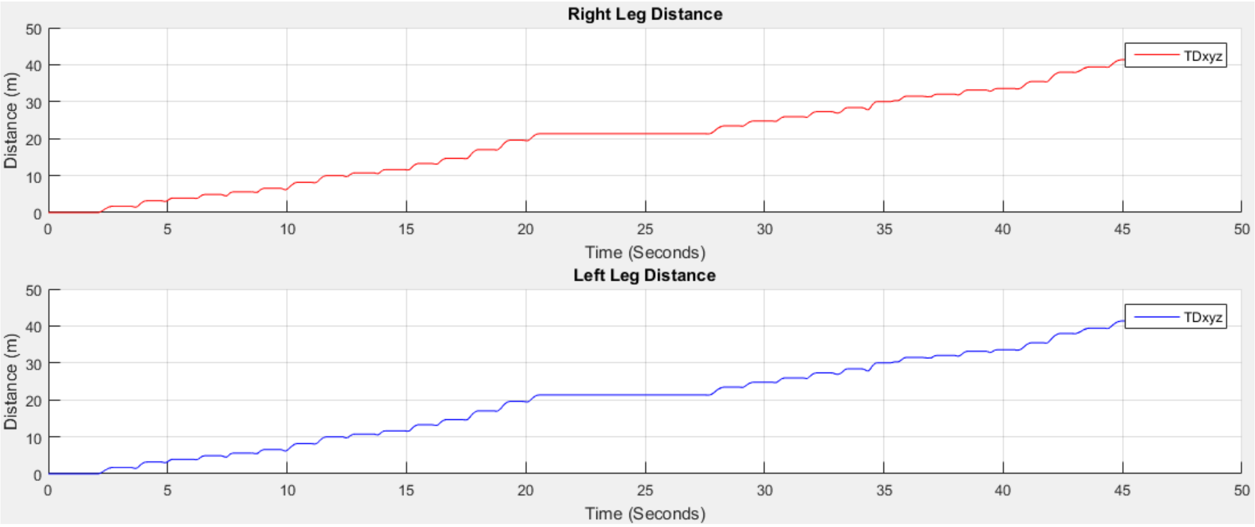


Figure 9.3: Result of distance estimation using proposed method

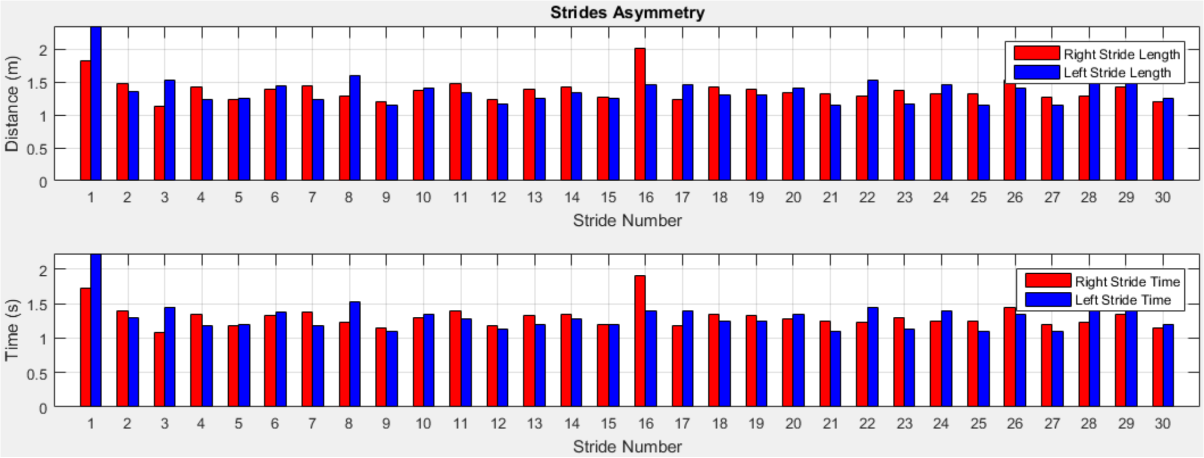


Figure 9.4: Stride asymmetry estimation of right and left legs

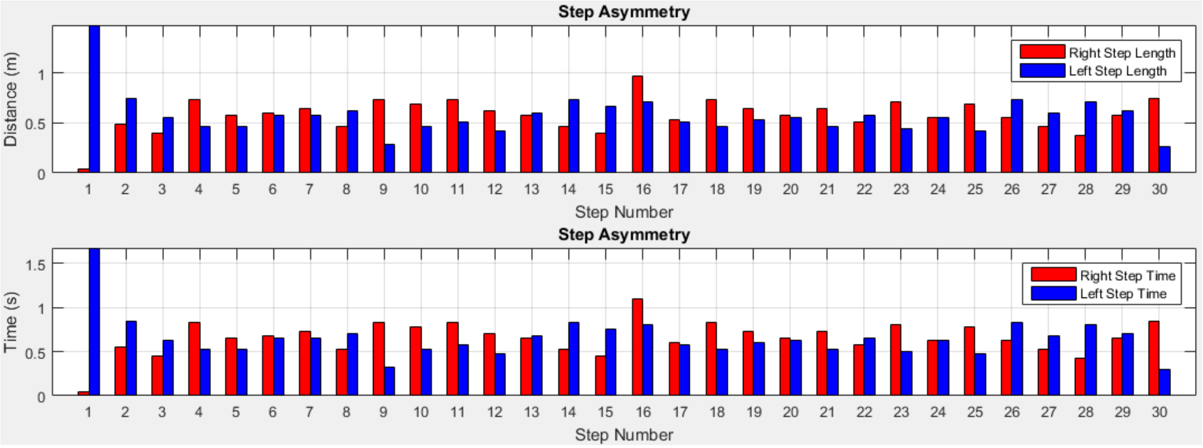


Figure 9.5: Step asymmetry estimation of right and left legs

10: Young Participant 10

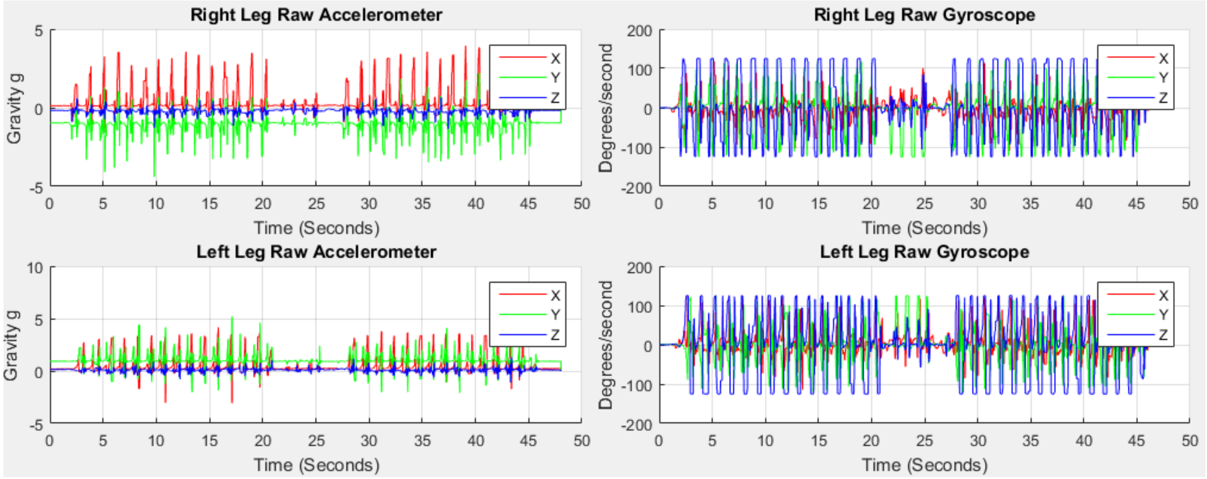


Figure 10.1: Accelerometer and gyroscope data from right and left legs

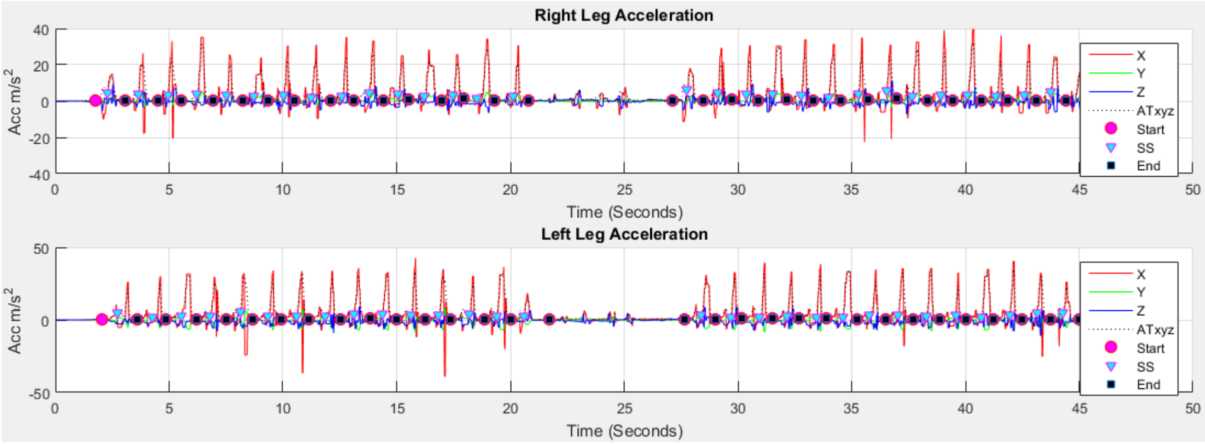


Figure 10.2: Result of stride, stance and swing event detection using proposed method

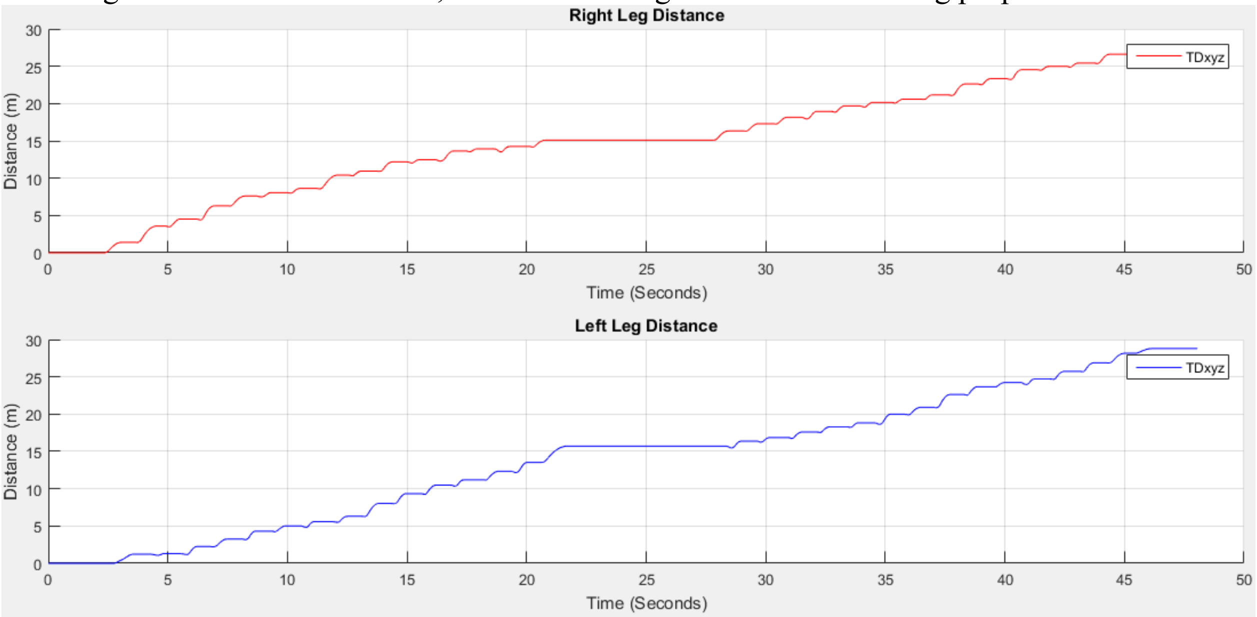

Figure 10.3: Result of distance estimation using proposed method

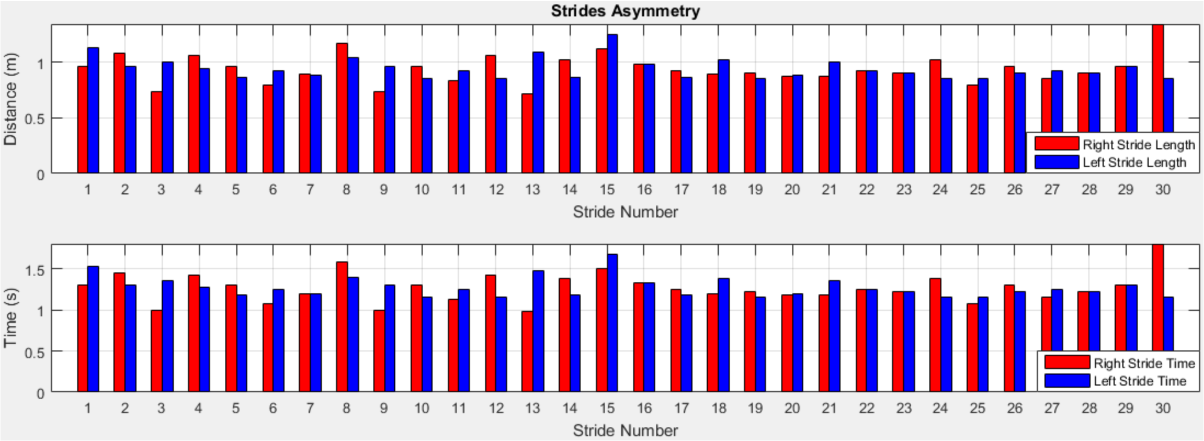


Figure 10.4: Stride asymmetry estimation of right and left legs

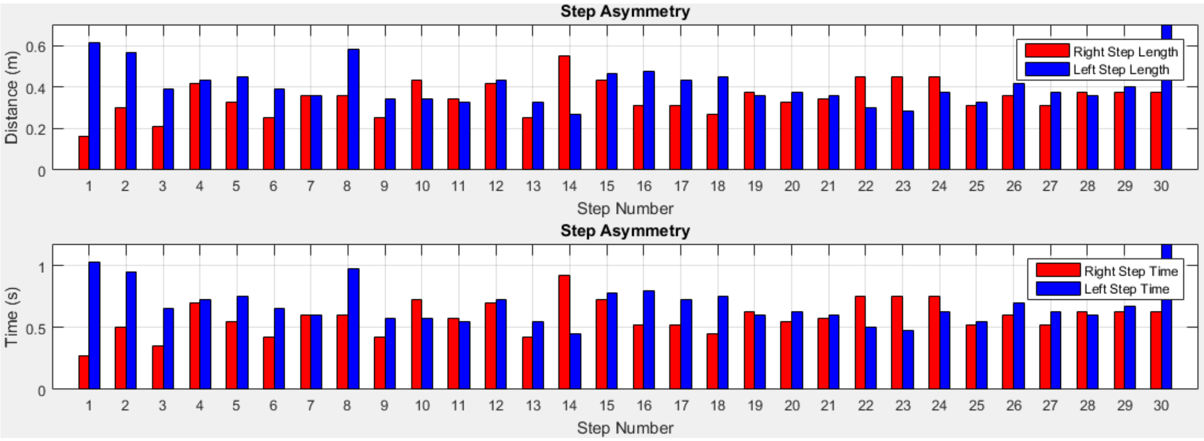


Figure 10.5: Step asymmetry estimation of right and left legs

1:Elderly Participant 1

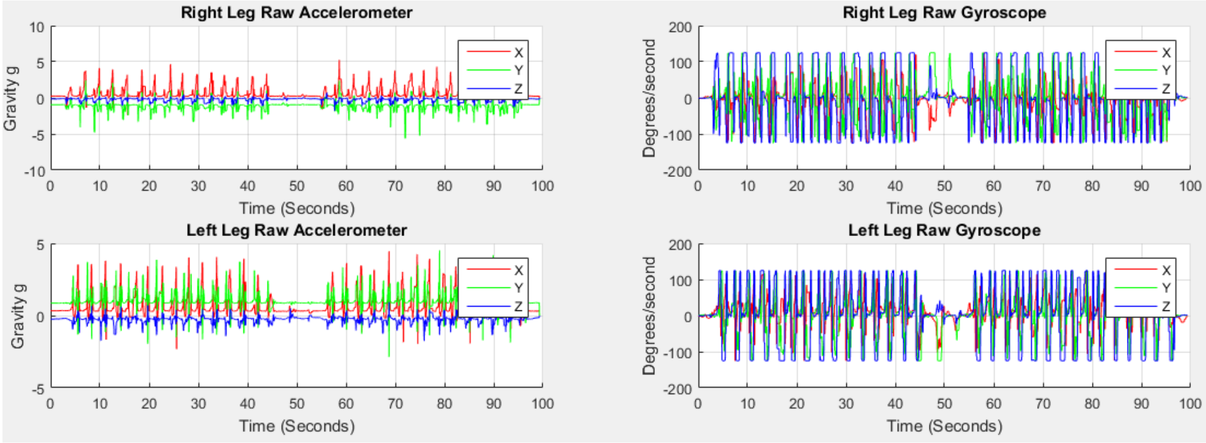


Figure 1.1: Accelerometer and gyroscope data from right and left foots

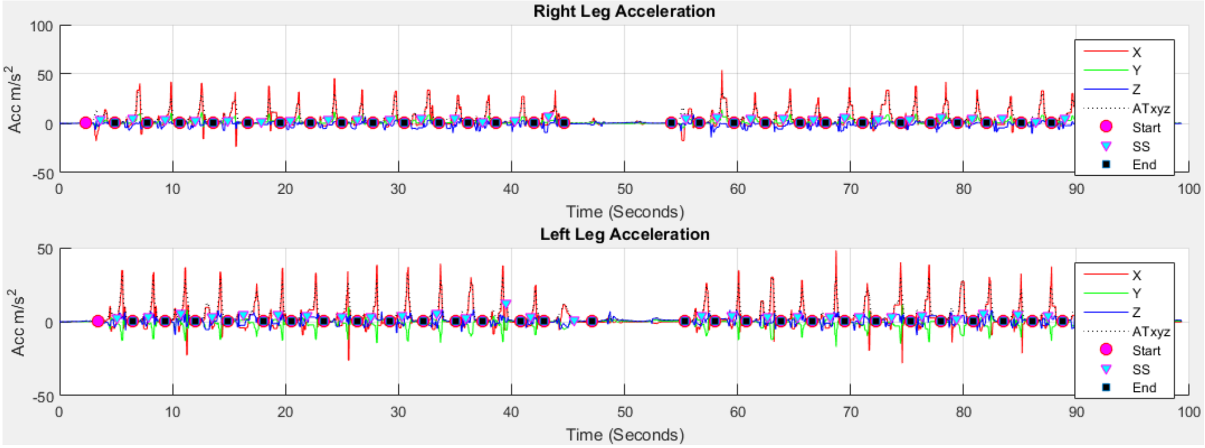


Figure 1.2: Result of stride, stance and swing event detection using proposed method

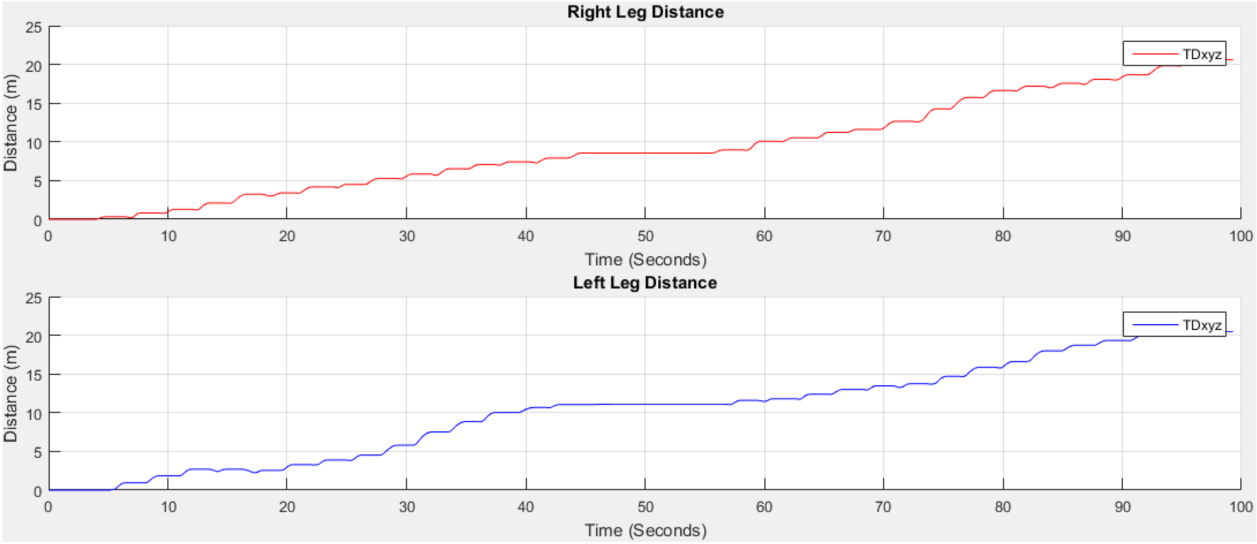

Figure 1.3: Result of distance estimation using proposed method

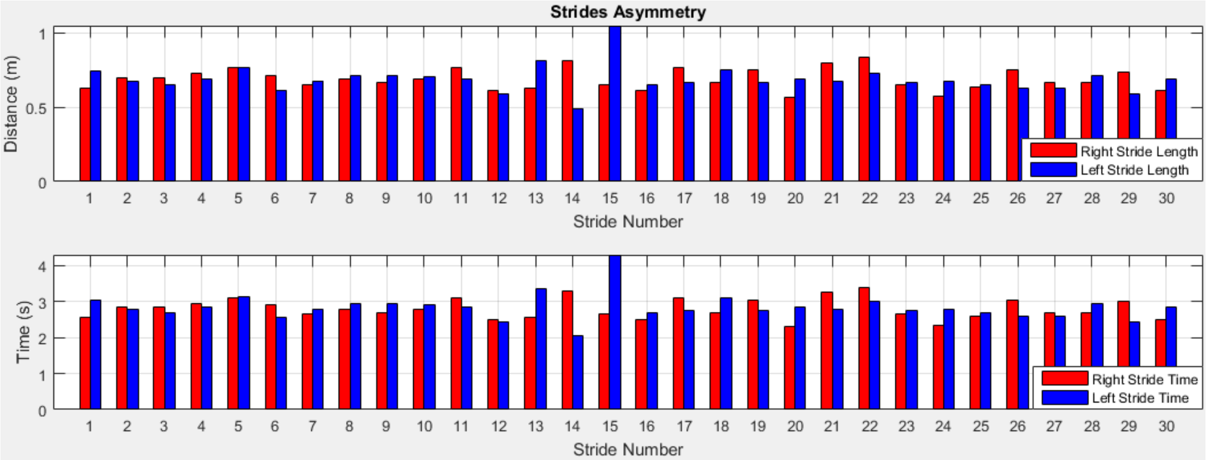


Figure 1.4: Stride asymmetry estimation of right and left legs

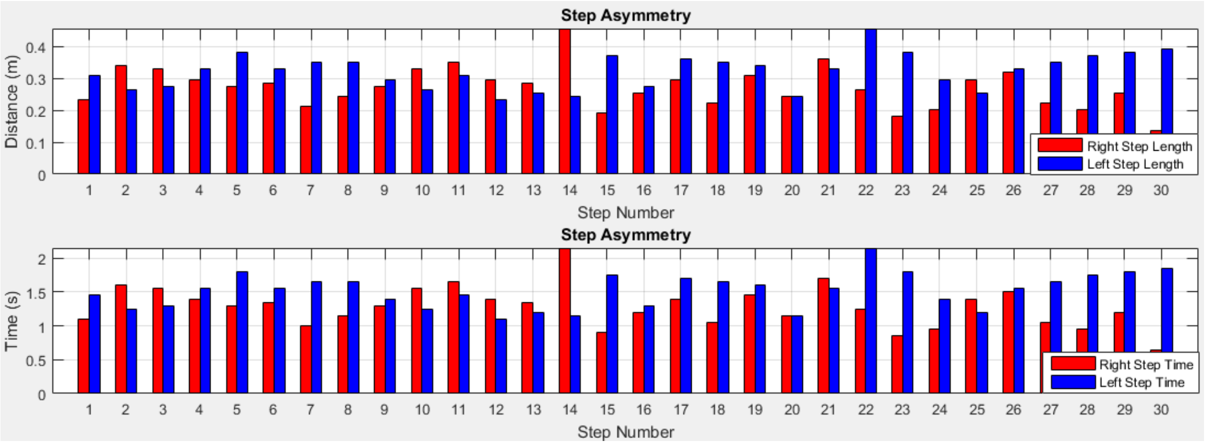


Figure 1.5: Step asymmetry estimation of right and left legs

2: Elderly Participant 2

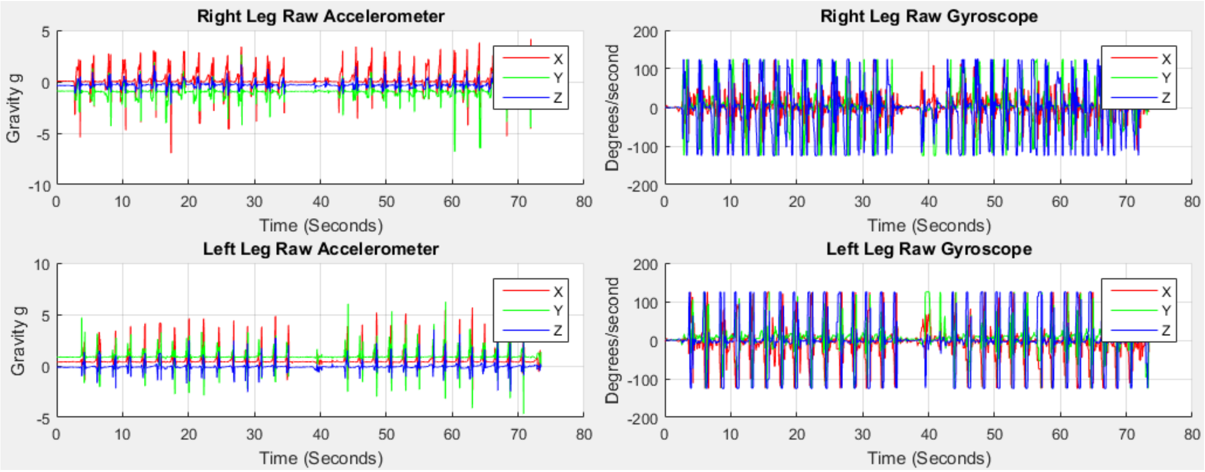


Figure 2.1: Accelerometer and gyroscope data from right and left legs

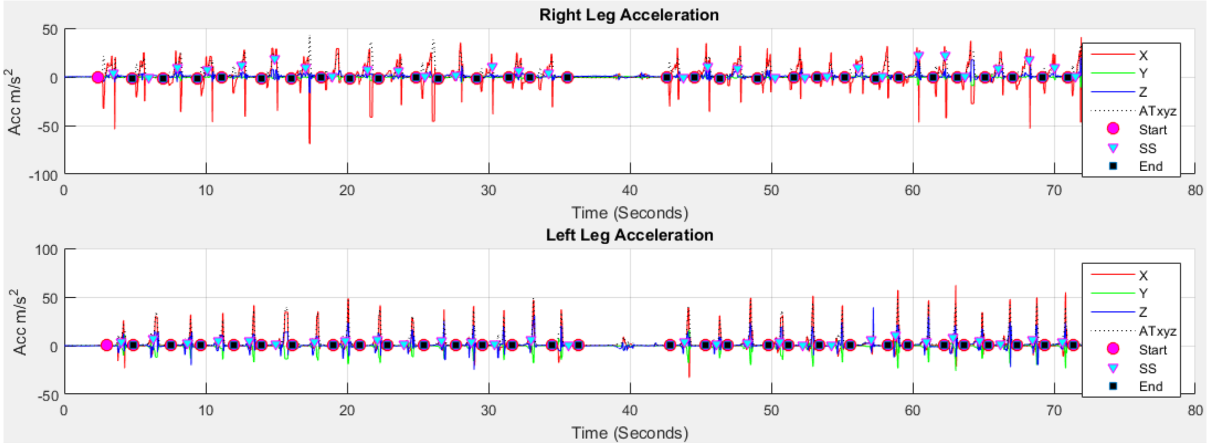


Figure 2.2: Result of stride, stance and swing event detection using proposed method

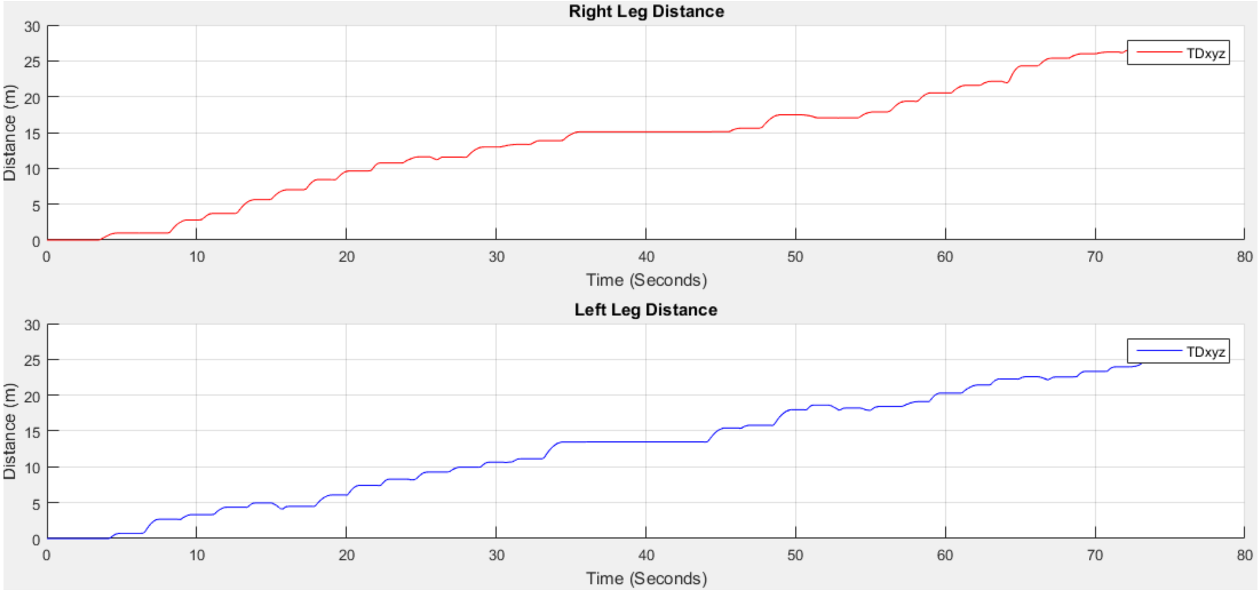


Figure 2.3: Result of distance estimation using proposed method

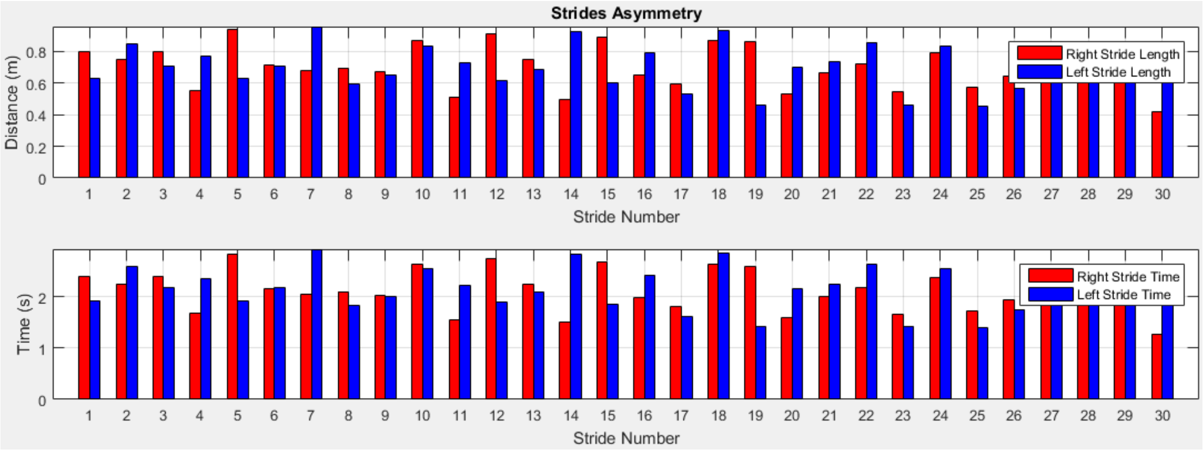

Figure 2.4: Stride asymmetry estimation of right and left legs

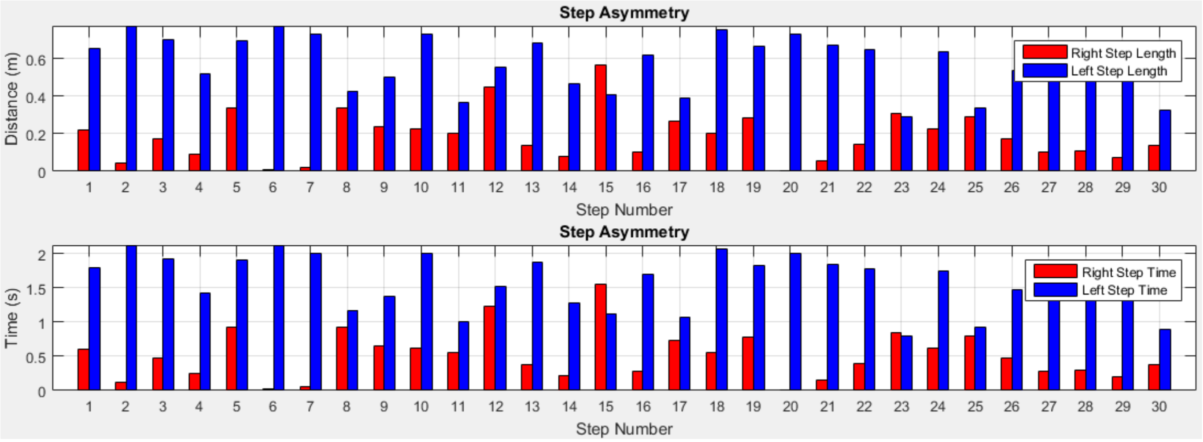

Figure 2.5: Step asymmetry estimation of right and left legs

3: Elderly Participant 3

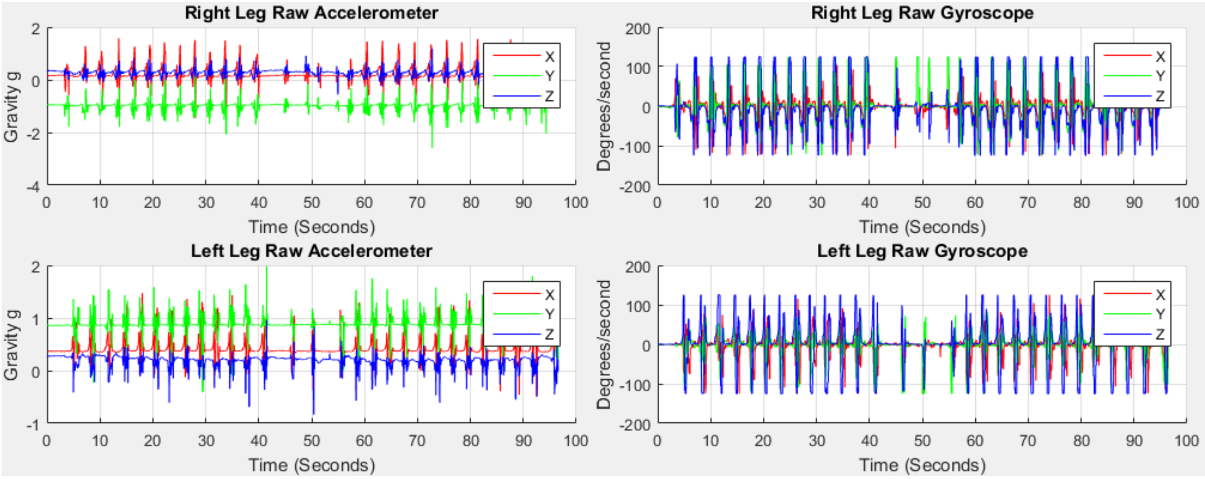

Figure 3.1: Accelerometer and gyroscope data from right and left legs

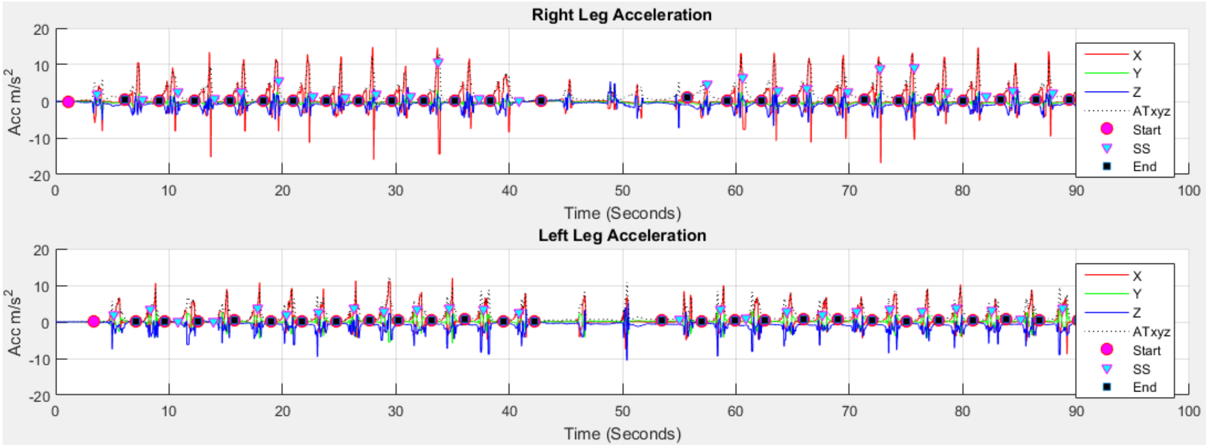

Figure 3.2: Result of stride, stance and swing event detection using proposed method

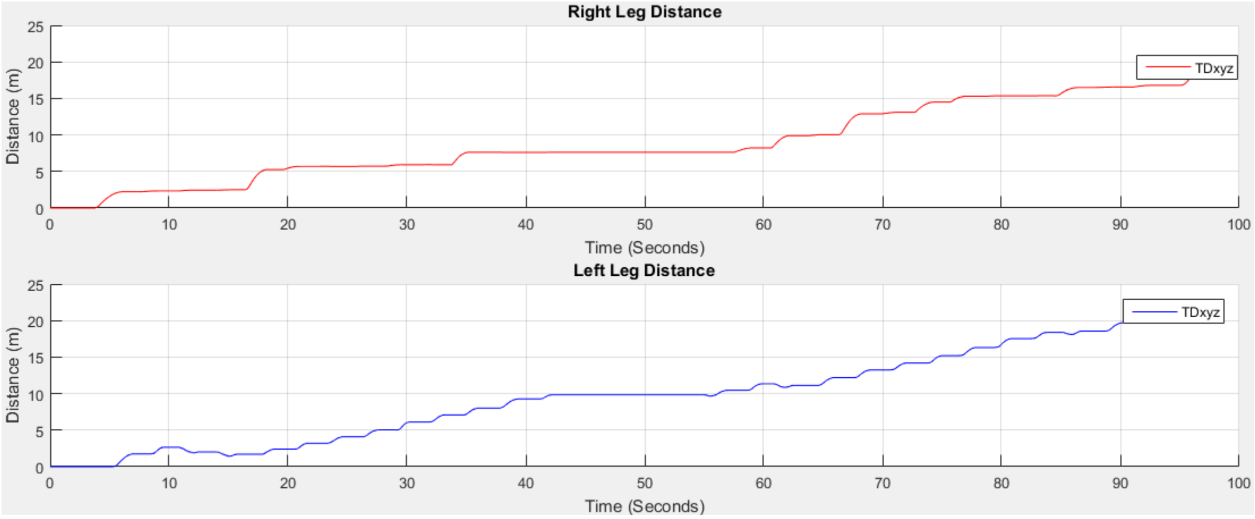

Figure 3.3: Result of distance estimation using proposed method

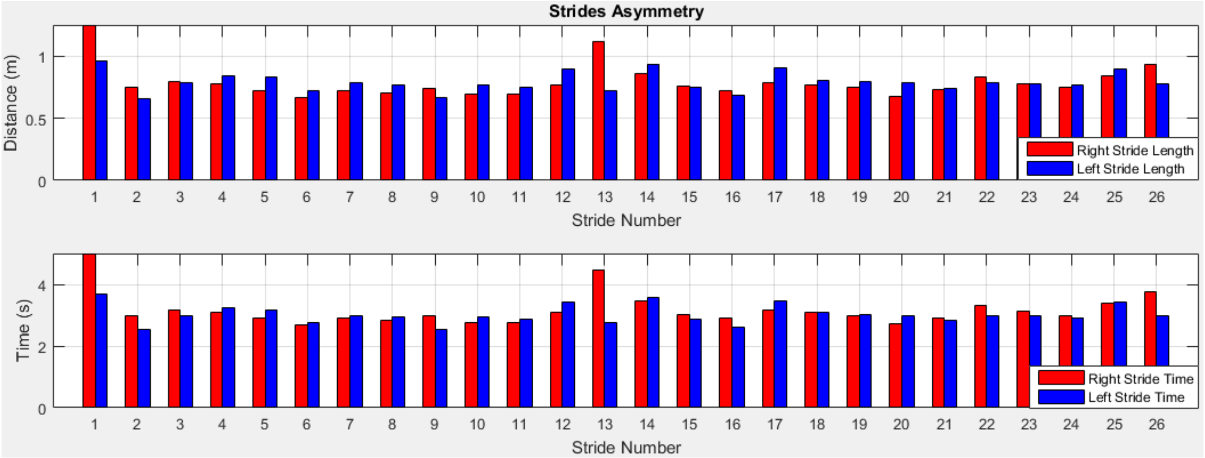

Figure 3.4: Stride asymmetry estimation of right and left legs

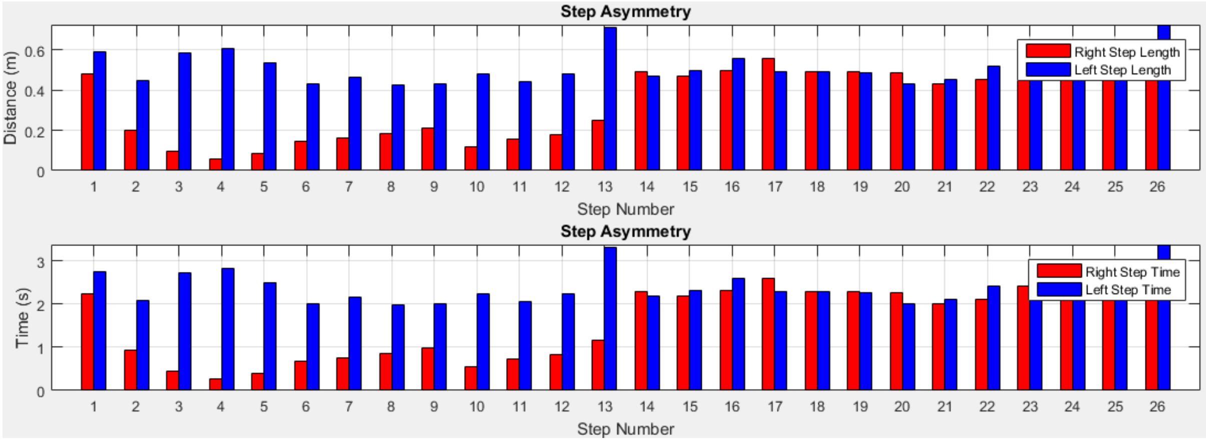

Figure 3.5: Step asymmetry estimation of right and left legs

4: Elderly Participant 4

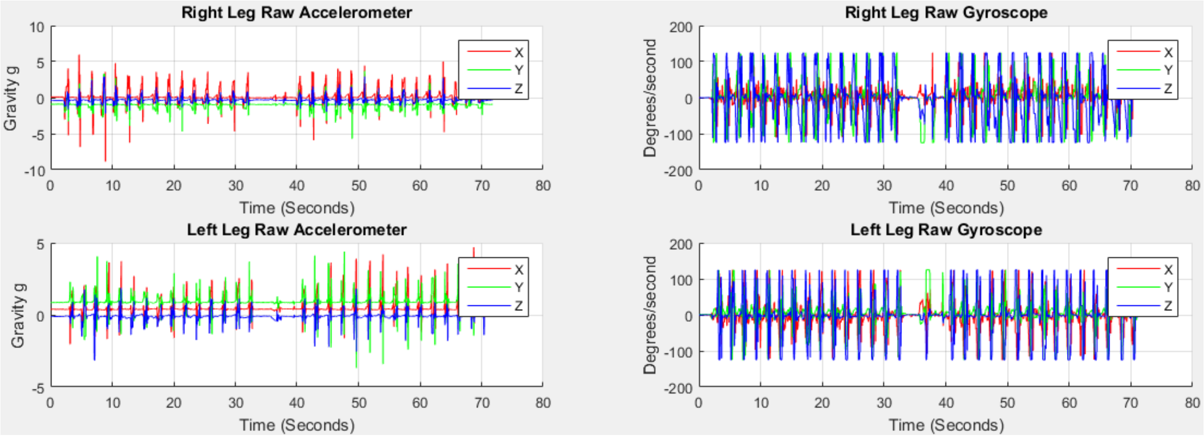


Figure 4.1: Accelerometer and gyroscope data from right and left legs

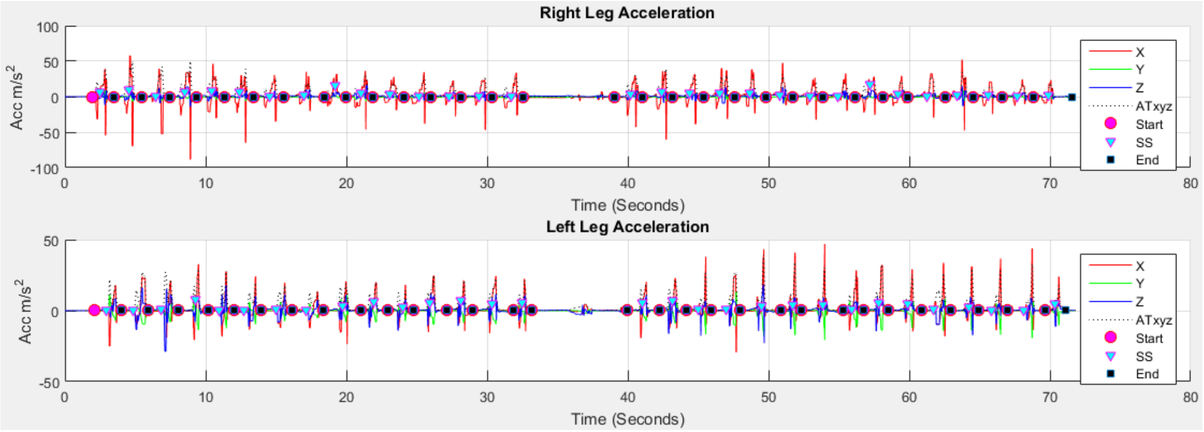


Figure 4.2: Result of stride, stance and swing event detection using proposed method

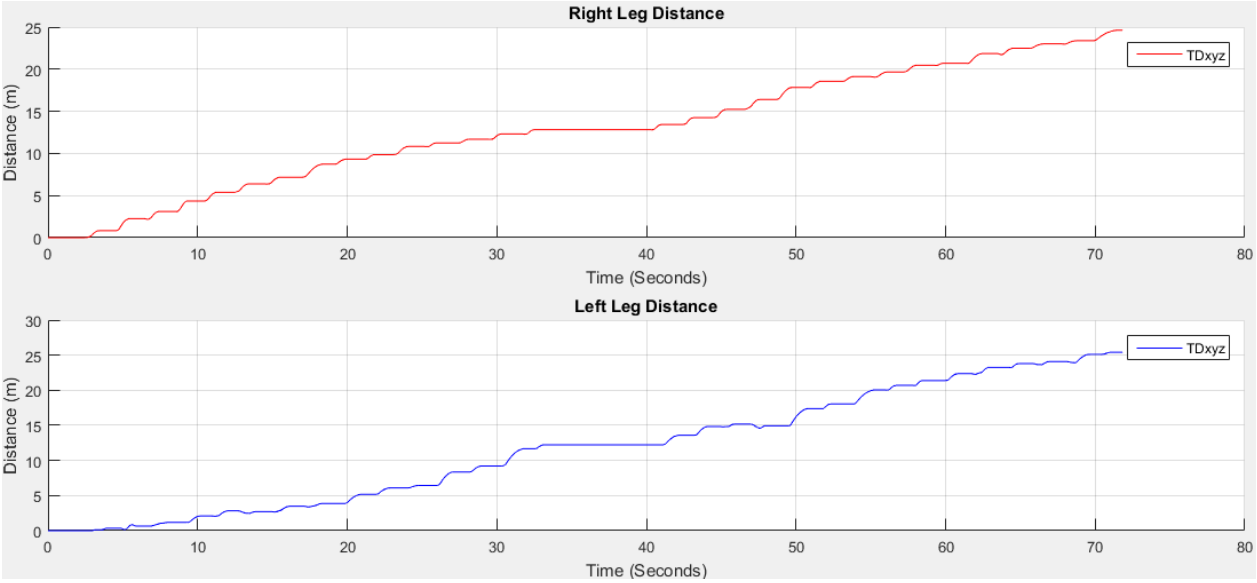


Figure 4.3: Result of distance estimation using proposed method

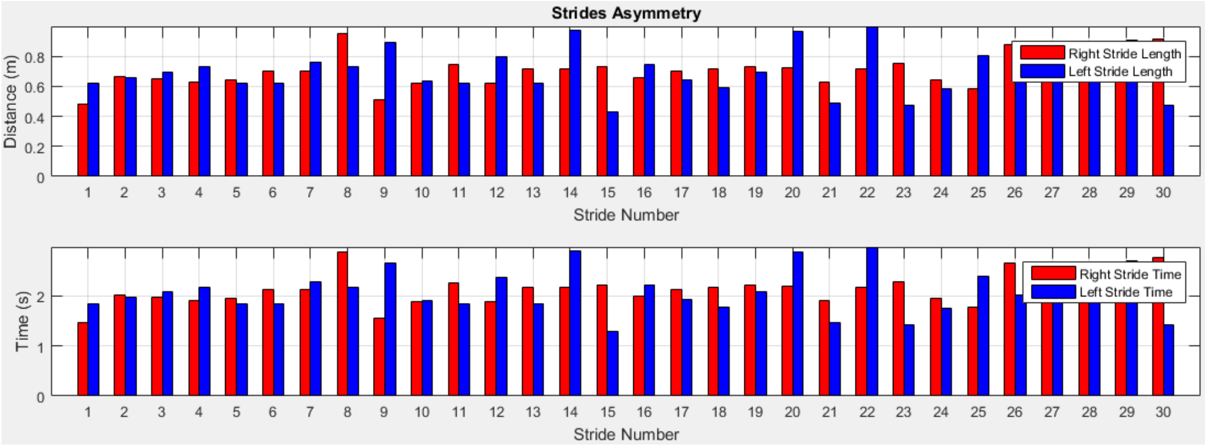


Figure 4.4: Stride asymmetry estimation of right and left legs

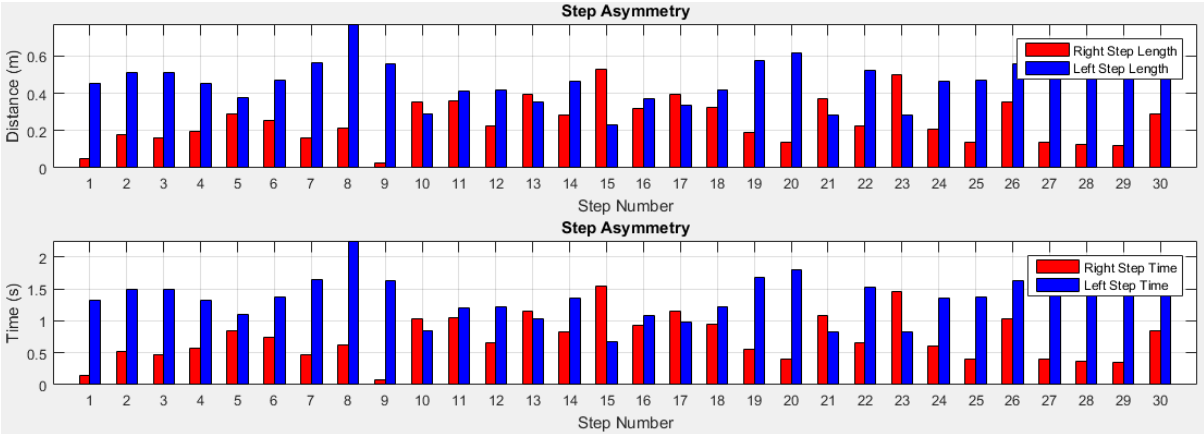


Figure 4.5: Step asymmetry estimation of right and left legs

## 5: Elderly Participant 5

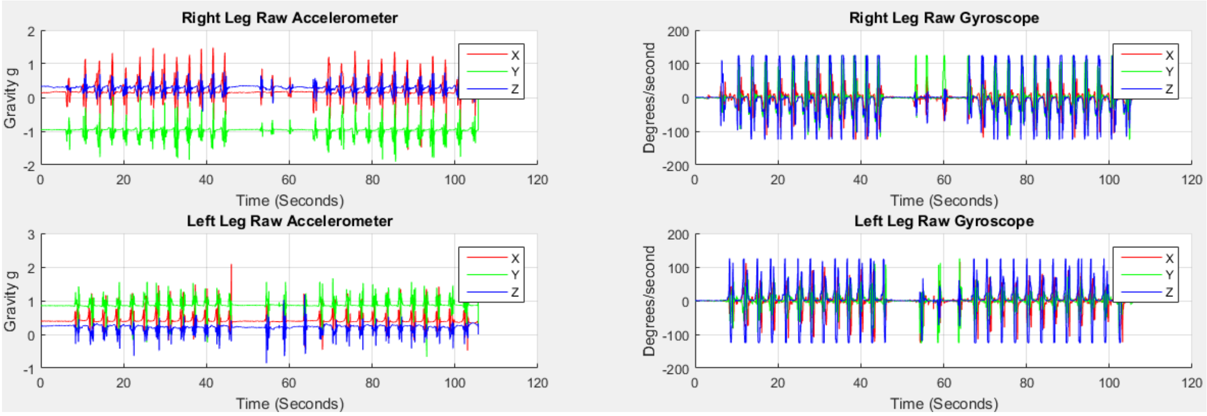


Figure 5.1: Accelerometer and gyroscope data from right and left legs

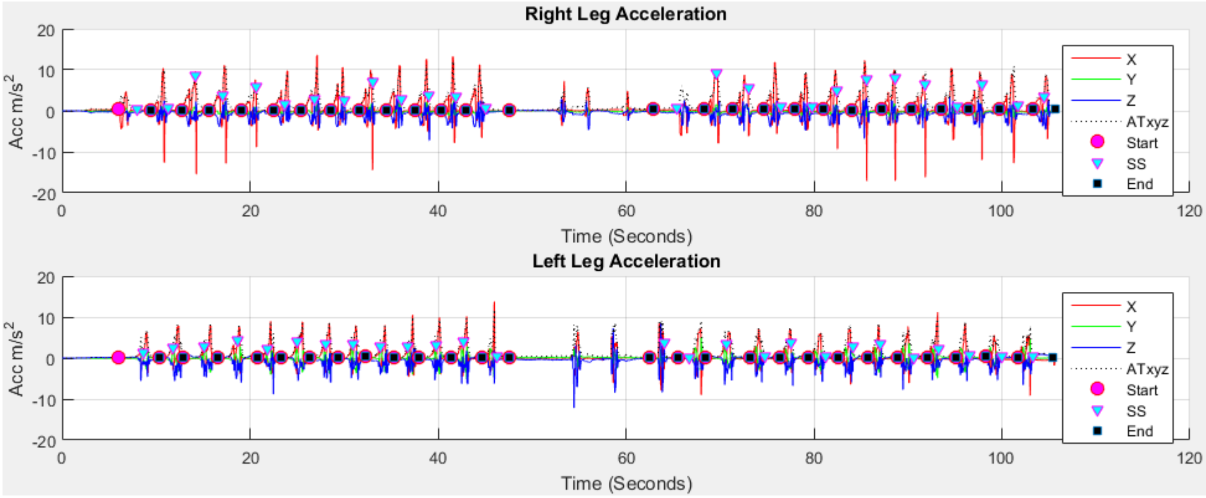


Figure 5.2: Result of stride, stance and swing event detection using proposed method

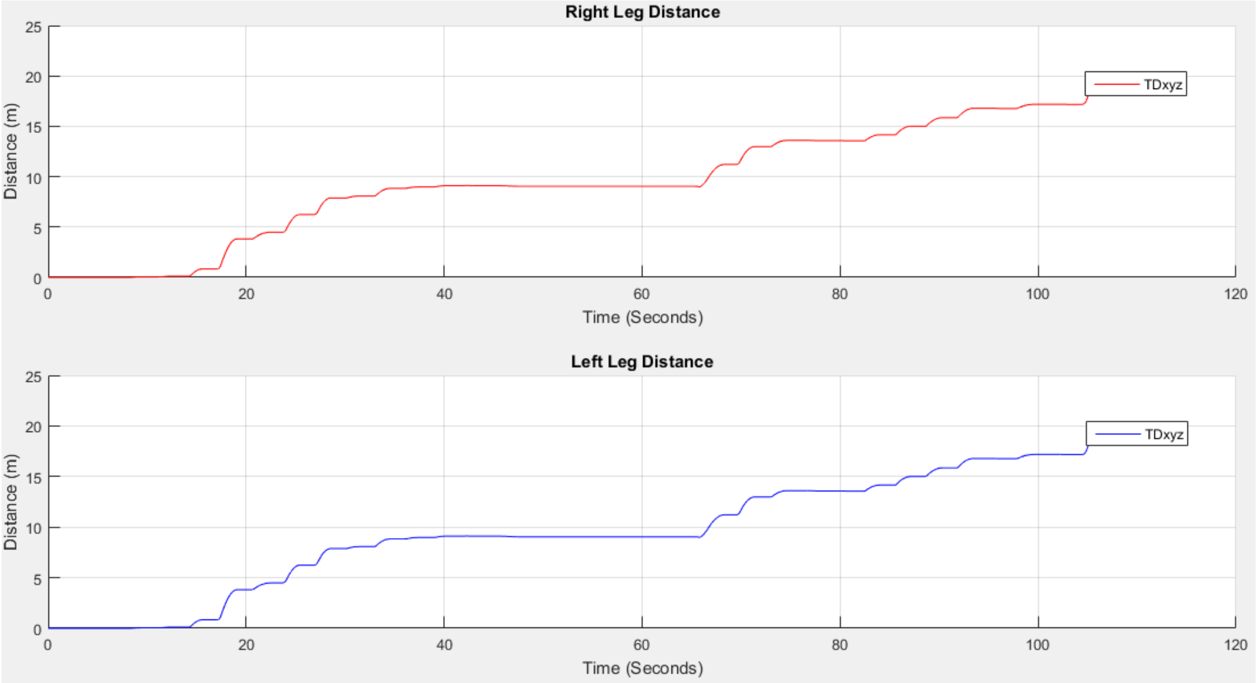


Figure 5.3: Result of distance estimation using proposed method

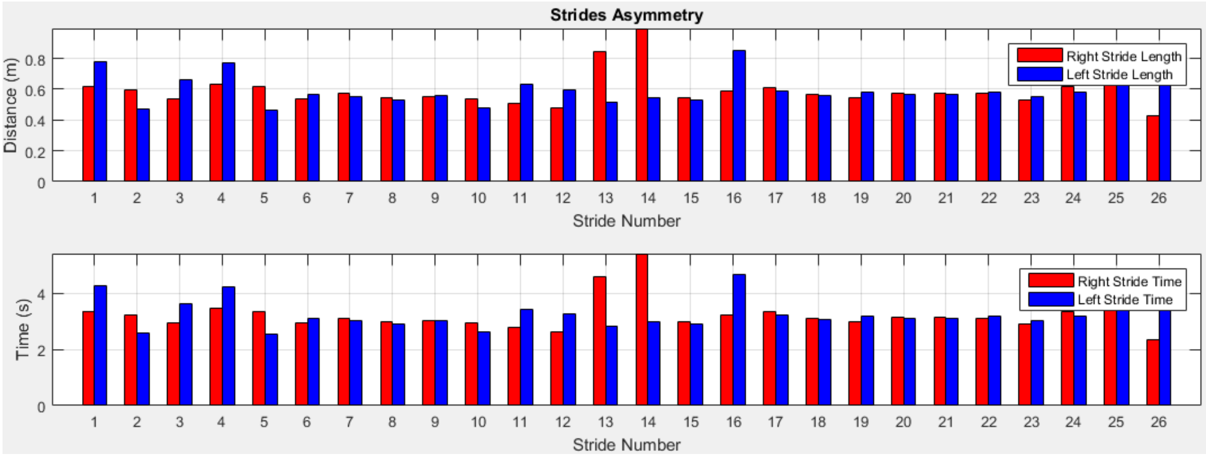


Figure 5.4: Stride asymmetry estimation of right and left legs

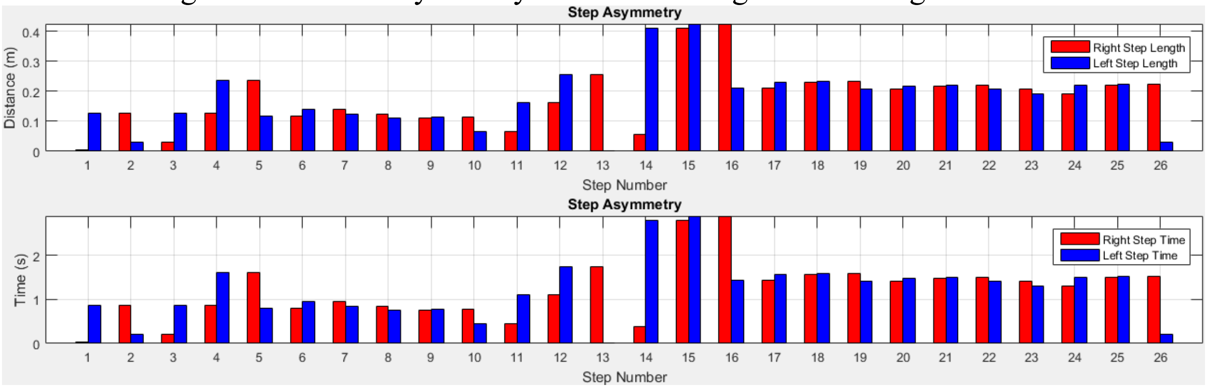


Figure 5.5: Step asymmetry estimation of right and left legs

6: Elderly Participant 6

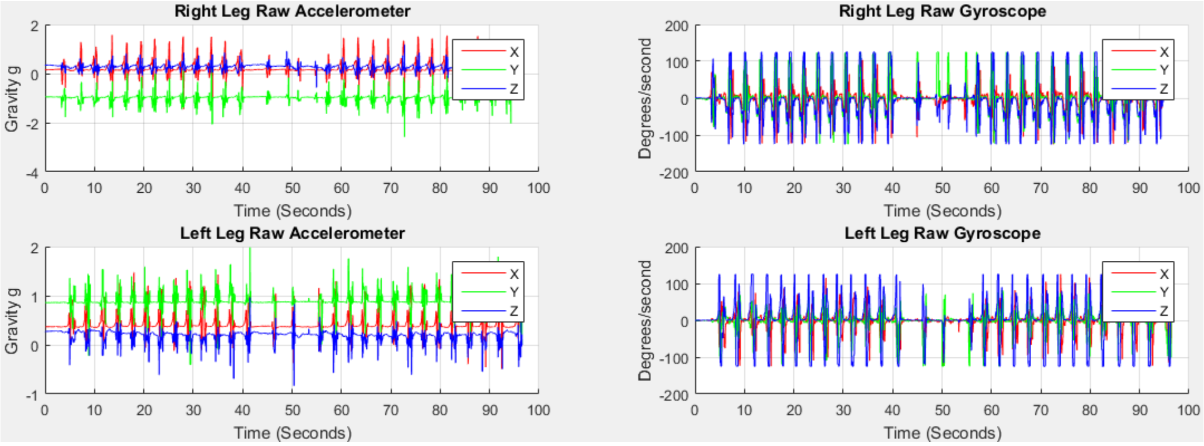


Figure 6.1: Accelerometer and gyroscope data from right and left legs

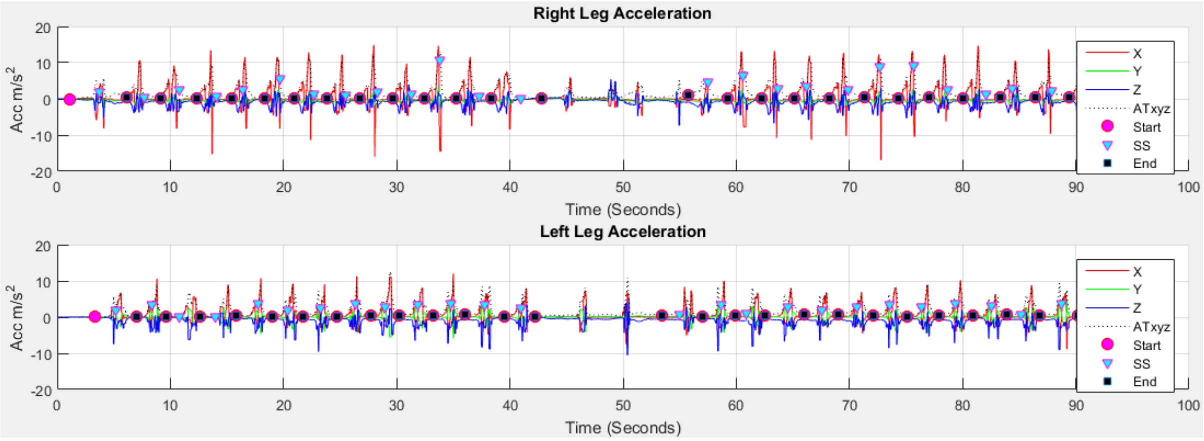


Figure 6.2: Result of stride, stance and swing event detection using proposed method

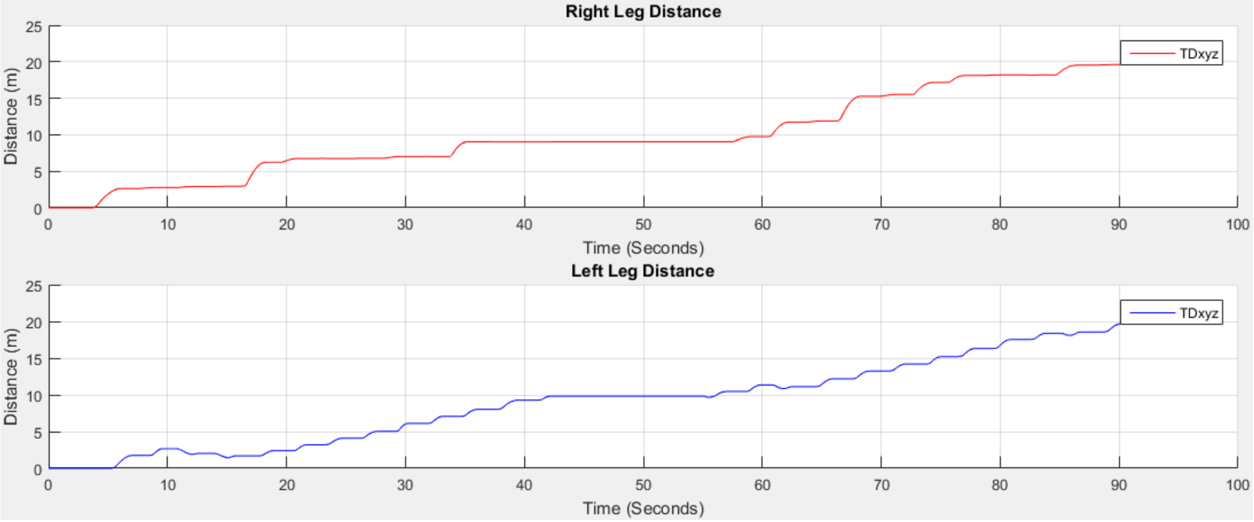


Figure 6.3: Result of distance estimation using proposed method

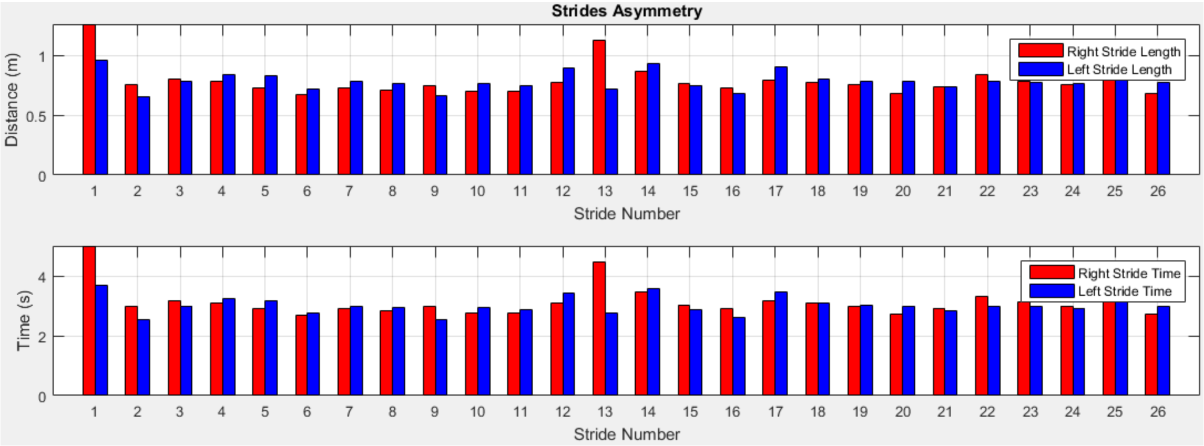


Figure 6.4: Stride asymmetry estimation of right and left legs

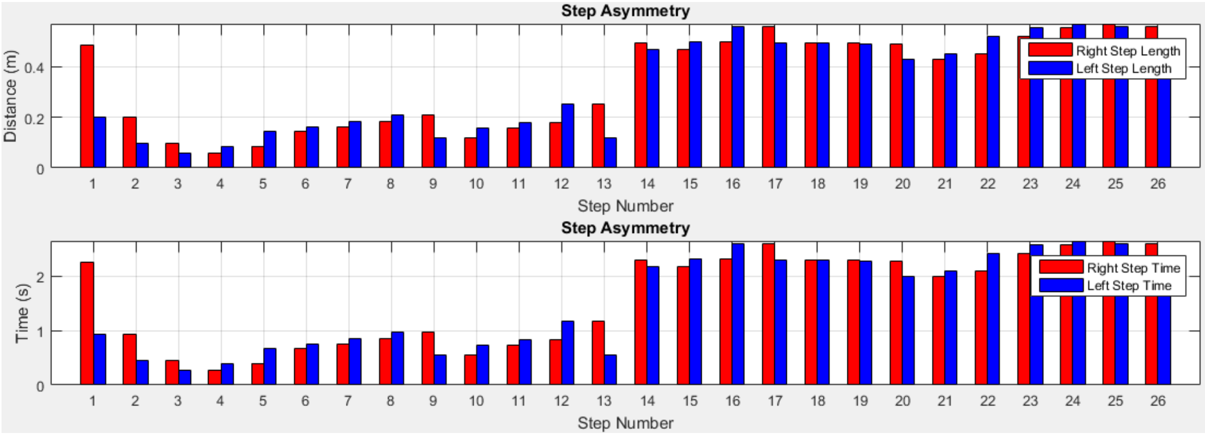

Figure 6.5: Step asymmetry estimation of right and left legs

7: Elderly Participant 7

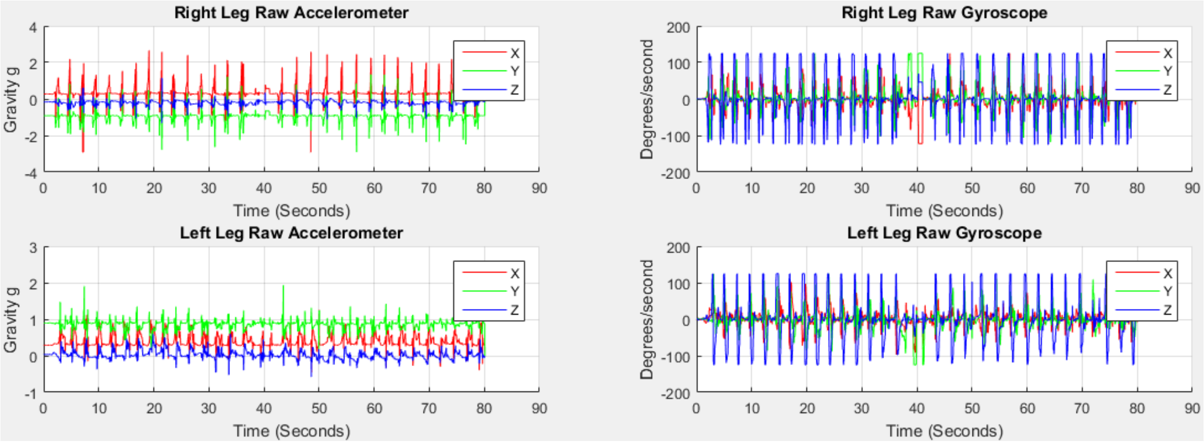

Figure 7.1: Accelerometer and gyroscope data from right and left legs

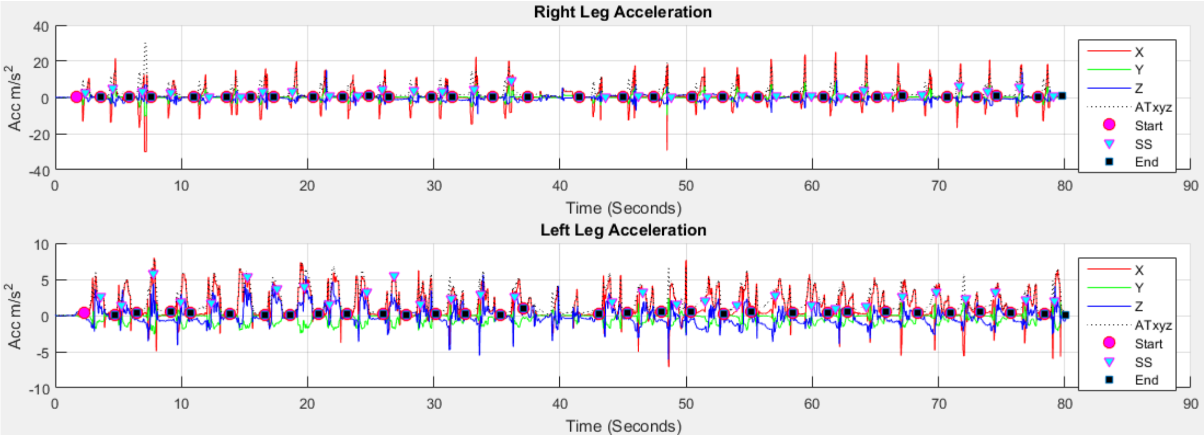

Figure 7.2: Result of stride, stance and swing event detection using proposed method

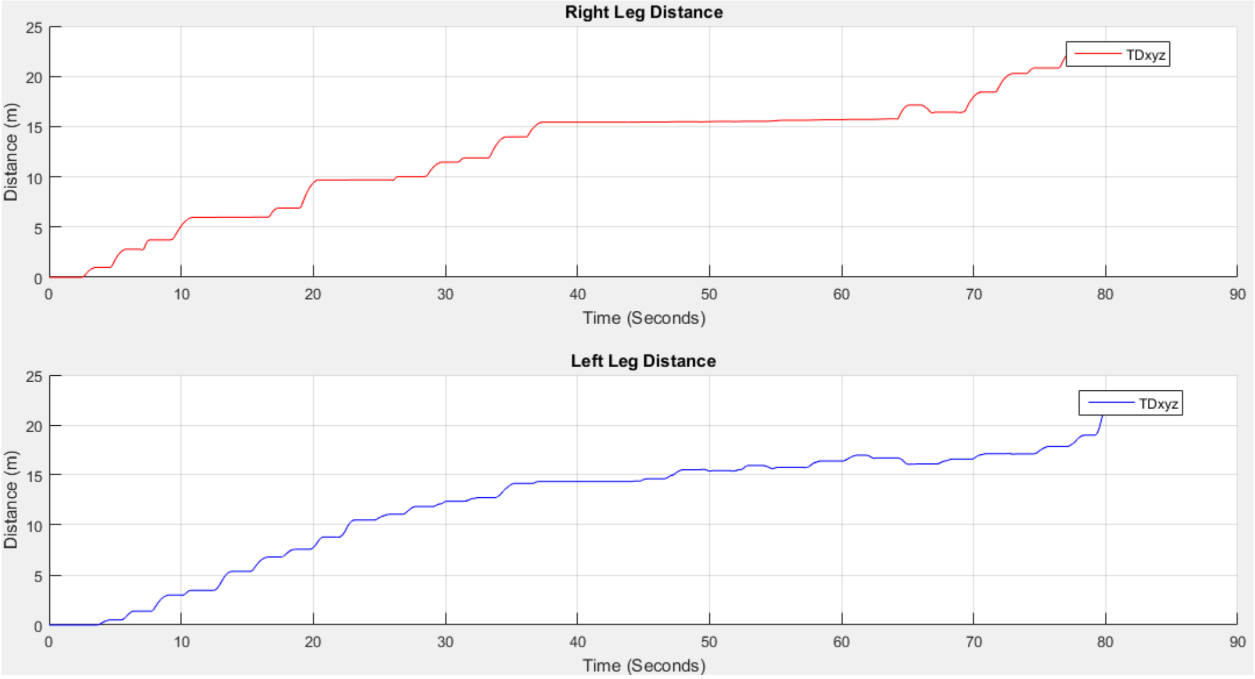

Figure 7.3: Result of distance estimation using proposed method

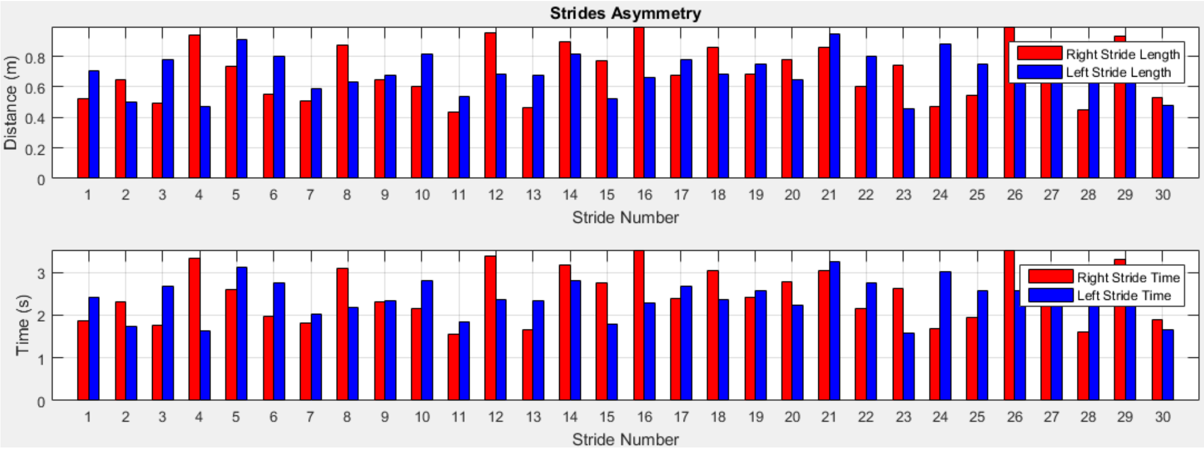

Figure 7.4: Stride asymmetry estimation of right and left legs

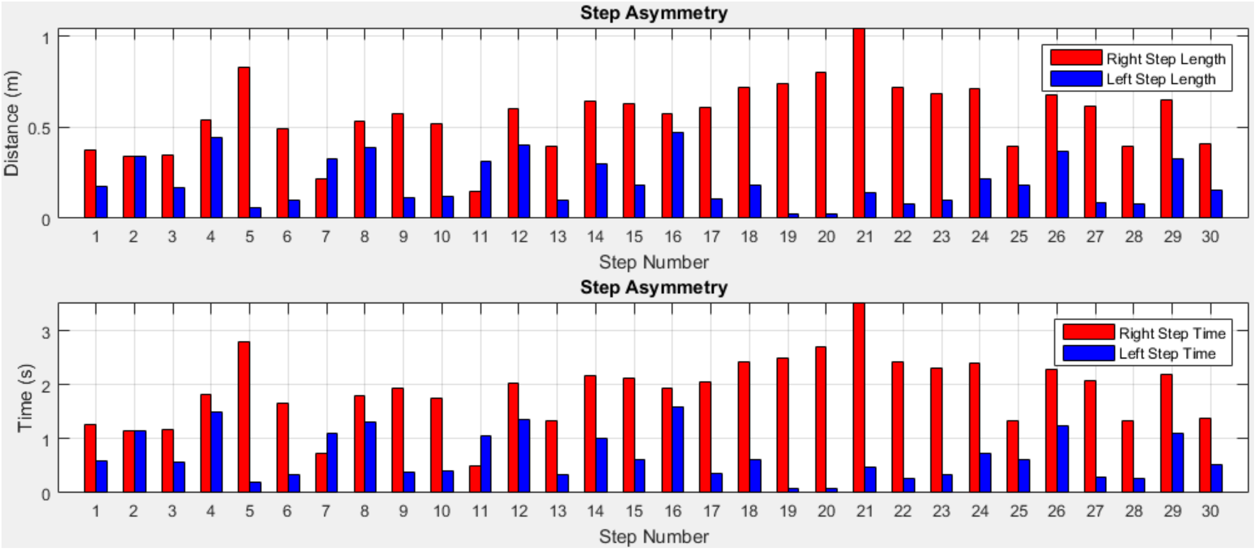

Figure 7.5: Step asymmetry estimation of right and left legs

8: Elderly Participant 8

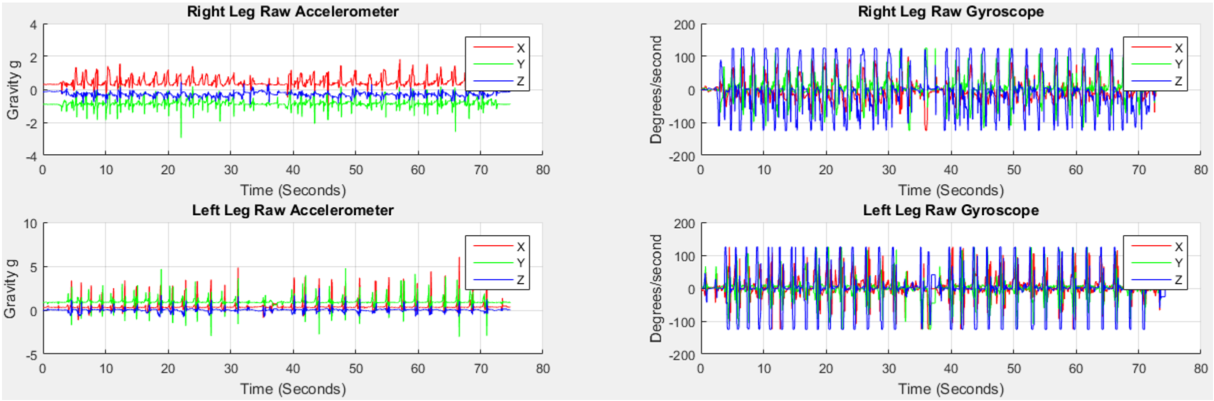

Figure 8.1: Accelerometer and gyroscope data from right and left legs

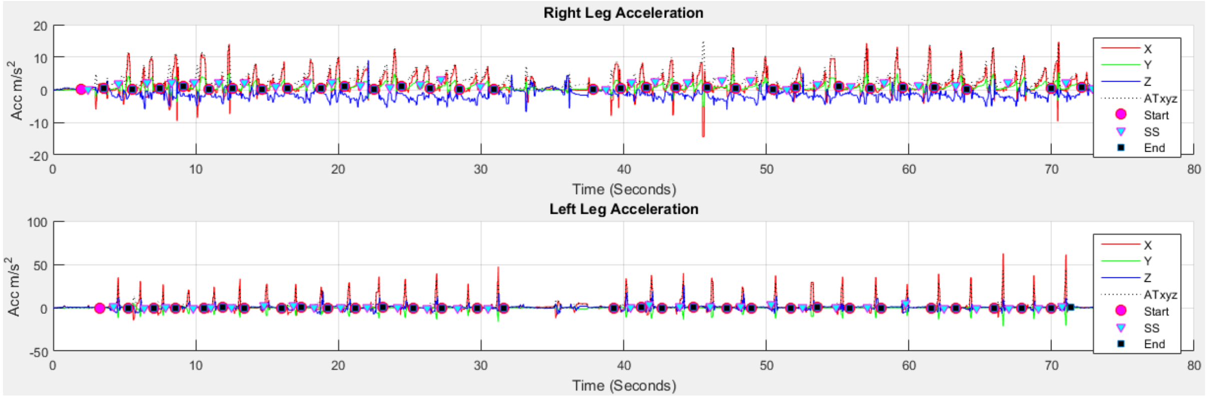

Figure 8.2: Result of stride, stance and swing event detection using proposed method

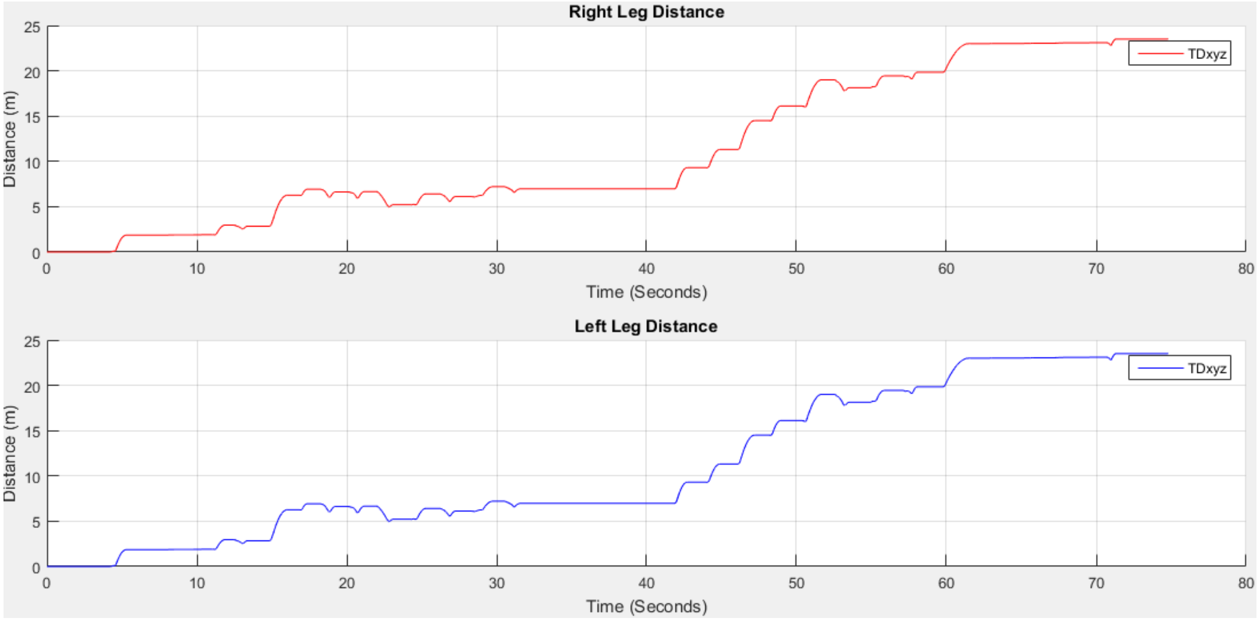


Figure 8.3: Result of distance estimation using proposed method

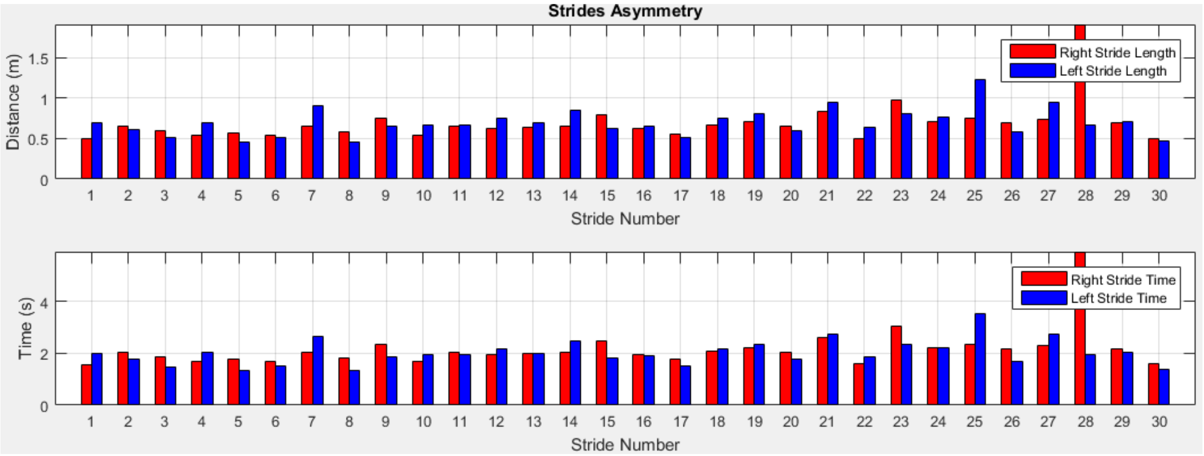


Figure 8.4: Stride asymmetry estimation of right and left legs

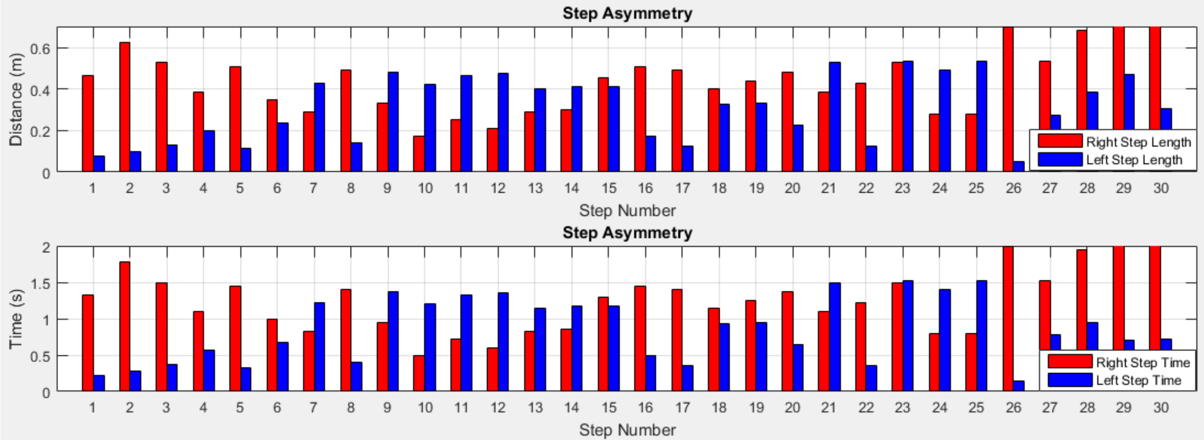


Figure 8.5: Step asymmetry estimation of right and left legs

9: Elderly Participant 9

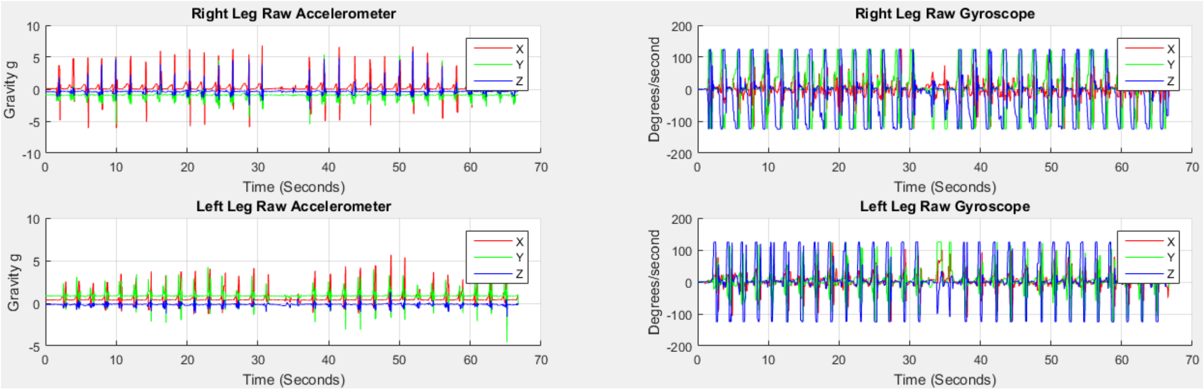

Figure 9.1: Accelerometer and gyroscope data from right and left legs

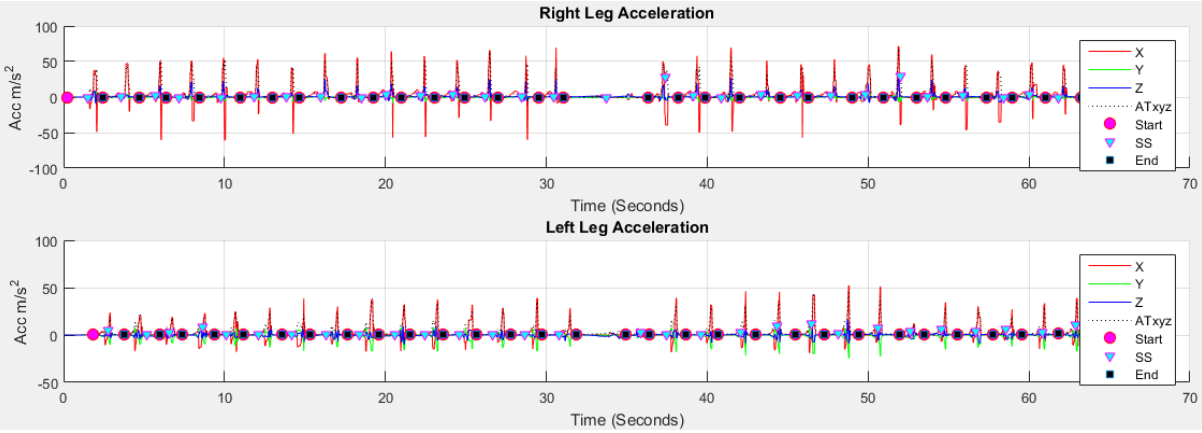

Figure 9.2: Result of stride, stance and swing event detection using proposed method

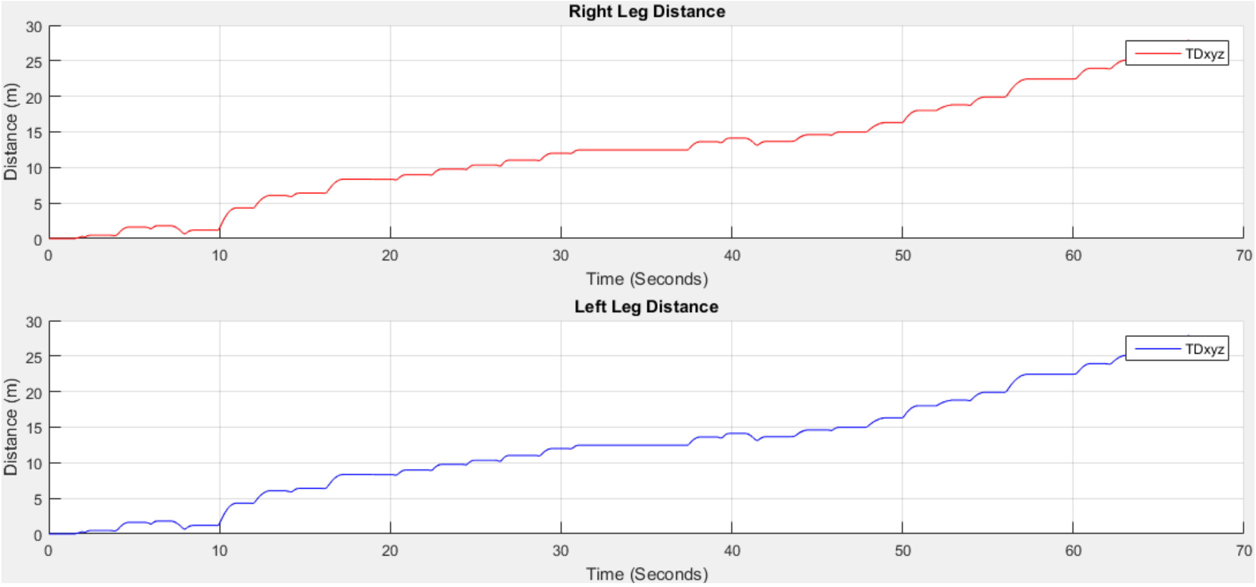

Figure 9.3: Result of distance estimation using proposed method

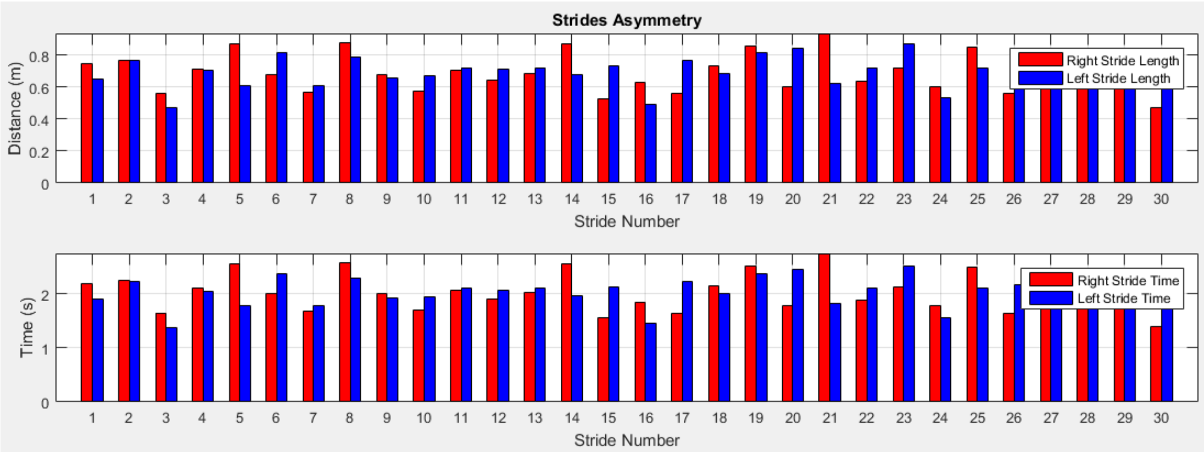

Figure 9.4: Stride asymmetry estimation of right and left legs

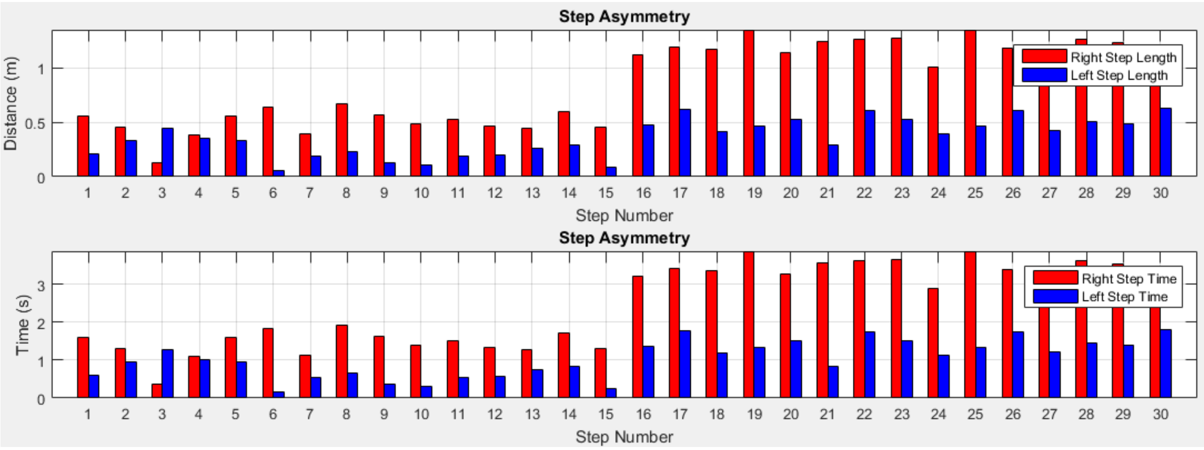

Figure 9.5: Step asymmetry estimation of right and left legs

10: Elderly Participant 10

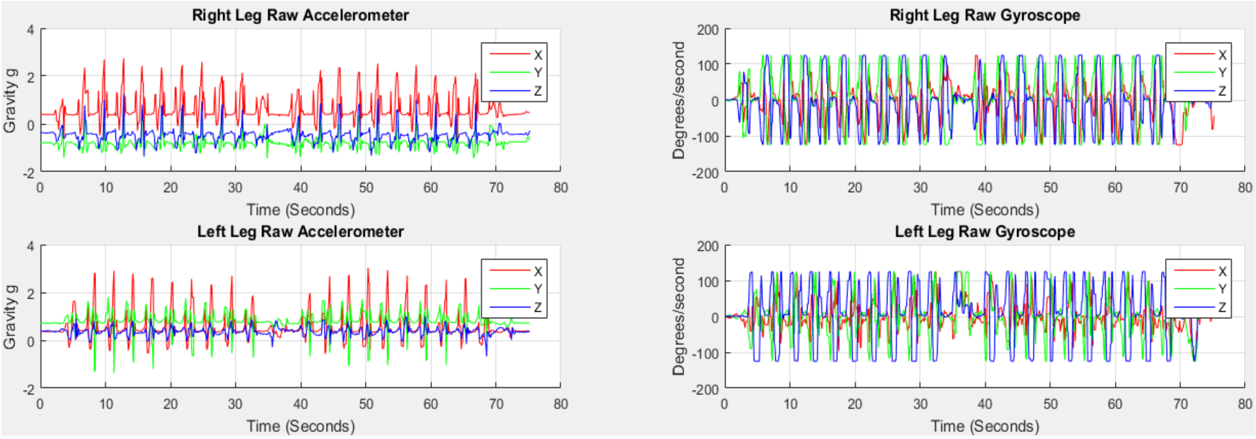

Figure 10.1: Accelerometer and gyroscope data from right and left legs

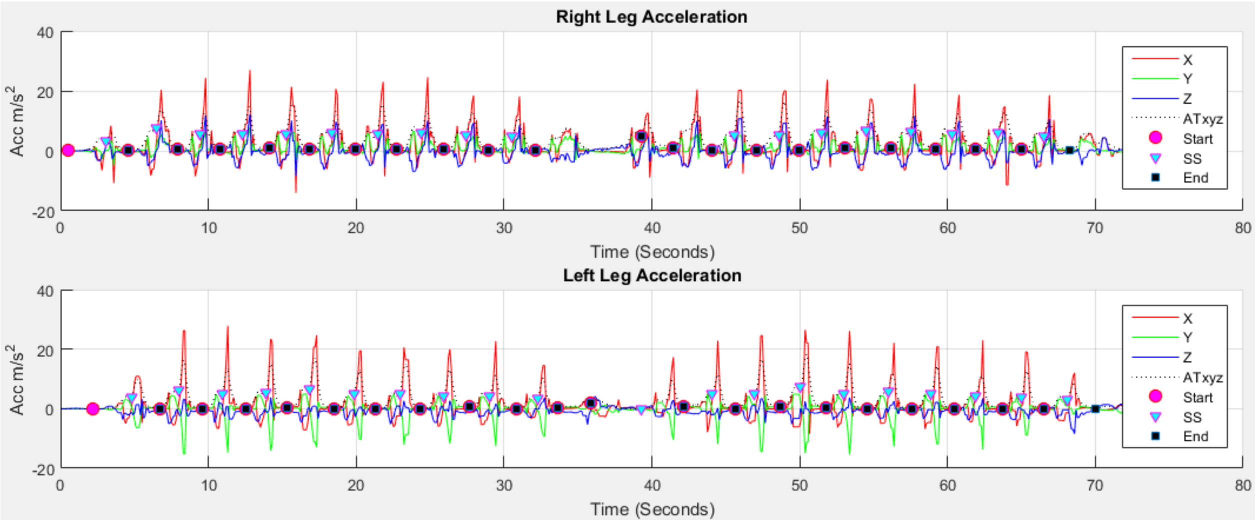

Figure 10.2: Result of stride, stance and swing event detection using proposed method

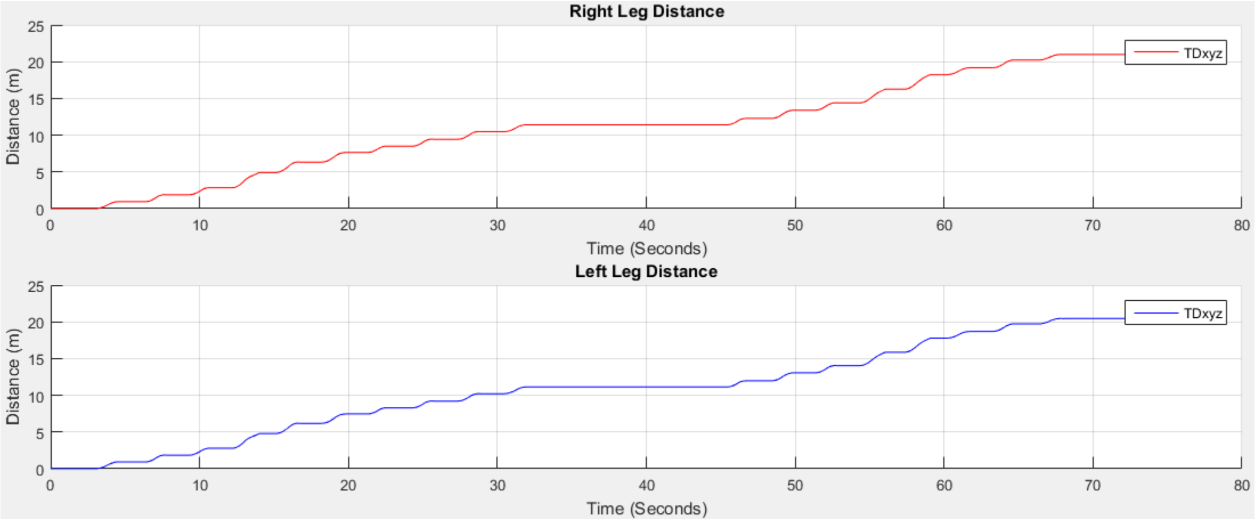

Figure 10.3: Result of distance estimation using proposed method

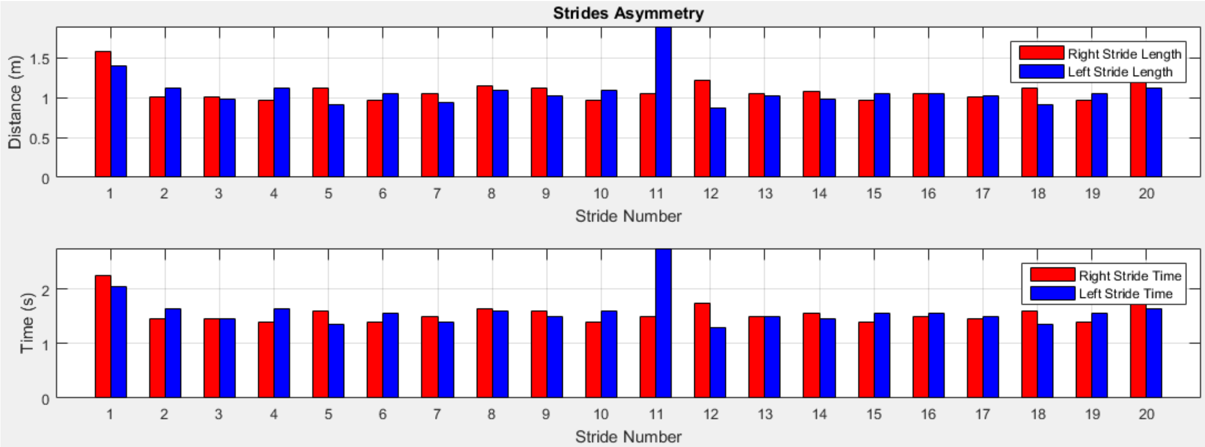

Figure 10.4: Stride asymmetry estimation of right and left legs

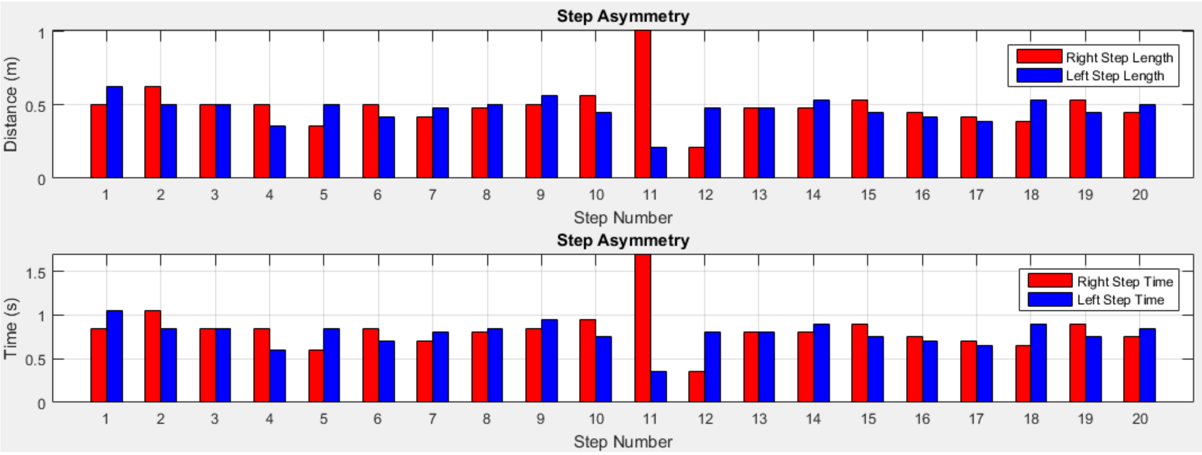


Figure 10.5: Step asymmetry estimation of right and left legs

| Young Participant 1 | Age | Height (m) | Weight (Kg) | Gender | | | | | | |
|---|---|---|---|---|---|---|---|---|---|---|
| | 23.0000 | 1.6500 | 60.0000 | Male | | | | | | |
| | | | | | | | | | | |
| **Total Time (s)** | 52.0500 | | | | | | | | | |
| | **Actual** | **Right Leg** | **Accuracy** | **Left Leg** | **Accuracy** | | | | | |
| **Total Distance (m)** | 38.6600 | 37.2979 | 96.4767 | 38.5012 | 99.5892 | | | | | |
| **Estimated Velocity (m/s)** | 0.7427 | 0.7166 | 96.4767 | 0.7397 | 99.5892 | | | | | |
| **Detected Stride Number** | 30.0000 | 30.0000 | 100.0000 | 30.0000 | 100.0000 | | | | | |
| **Detected Step Number** | 30.0000 | 30.0000 | 100.0000 | 30.0000 | 100.0000 | | | | | |
| | | | | | | | | | | |
| | **Right** | | | | | **Left** | | | | |
| **Gait Features** | **Mean** | **StaDev** | **Variance** | **MinRange** | **MaxRange** | **Mean** | **StaDev** | **Variance** | **MinRange** | **MaxRange** |
| **Stride Length (m)** | 1.2656 | 0.1588 | 0.0252 | 0.9540 | 1.6456 | 1.2665 | 0.2036 | 0.0415 | 0.9631 | 1.9032 |
| **Stride Time (s)** | 1.3523 | 0.1666 | 0.0277 | 1.0255 | 1.7508 | 1.4065 | 0.2221 | 0.0493 | 1.0755 | 2.1010 |
| **Stride Velocity (m/s)** | 0.9359 | 0.9359 | 0.9359 | | | 0.9005 | 0.9005 | 0.9005 | | |
| | | | | | | | | | | |
| **Cadence (step/min)** | 34.5821 | | | | | 34.5821 | | | | |
| **Step Speed (m/s)** | 0.9359 | 0.9535 | 0.9092 | | | 0.9005 | 0.9168 | 0.8404 | | |
| **Step length (m)** | 0.4688 | 0.1279 | 0.0164 | 0.1115 | 0.7617 | 0.5357 | 0.1314 | 0.0173 | 0.2787 | 0.7803 |
| **Step time(s)** | 0.6311 | 0.1722 | 0.0297 | 0.1501 | 1.0255 | 0.7212 | 0.1769 | 0.0313 | 0.3752 | 1.0505 |
| **Step Ratio (Step length/cadence)** | 0.01 | | | | | 0.01 | | | | |
| | | | | | | | | | | |
| **Stance Time (s)** | 0.6887 | 0.1510 | 0.0221 | | | 0.7337 | 0.1314 | 0.0163 | | |
| **Swing Length (m)** | 1.2510 | 0.5693 | 0.3241 | | | 1.2917 | 0.6358 | 0.4042 | | |
| **Swing Time (s)** | 0.6637 | 0.1115 | 0.0131 | | | 0.6728 | 0.1755 | 0.0308 | | |
| **Swing Velocity (m/s)** | 1.8850 | 1.8830 | 1.8830 | | | 1.9463 | 1.9158 | 1.9158 | | |

| Young Participant 2 | Age | Height (m) | Weight (Kg) | Gender | | | | | | |
|---|---|---|---|---|---|---|---|---|---|---|
| | 19.0000 | 1.1700 | 55.0000 | Female | | | | | | |
| | | | | | | | | | | |
| **Total Time (s)** | 51.2250 | | | | | | | | | |
| | **Actual** | **Right Leg** | **Accuracy** | **Left Leg** | **Accuracy** | | | | | |
| **Total Distance (m)** | 42.0600 | 41.8235 | 99.4378 | 43.4401 | 96.7187 | | | | | |
| **Estimated Velocity (m/s)** | 0.8211 | 0.8165 | 99.4378 | 0.8480 | 96.7187 | | | | | |
| **Detected Stride Number** | 30.0000 | 30.0000 | 100.0000 | 30.0000 | 100.0000 | | | | | |
| **Detected Step Number** | 30.0000 | 30.0000 | 100.0000 | 30.0000 | 100.0000 | | | | | |
| | | | | | | | | | | |
| | **Right** | | | | | **Left** | | | | |
| **Gait Features** | **Mean** | **StaDev** | **Variance** | **MinRange** | **MaxRange** | **Mean** | **StaDev** | **Variance** | **MinRange** | **MaxRange** |
| **Stride Length (m)** | 0.6894 | 0.1029 | 0.0106 | 0.5389 | 0.9701 | 0.6893 | 0.0975 | 0.0095 | 0.4854 | 1.0193 |
| **Stride Time (s)** | 1.4649 | 0.2148 | 0.0462 | 1.1506 | 2.0510 | 1.4457 | 0.2009 | 0.0404 | 1.0255 | 2.1260 |
| **Stride Velocity (m/s)** | 0.4706 | 0.4706 | 0.4706 | | | 0.4768 | 0.4768 | 0.4768 | | |
| | | | | | | | | | | |
| **Cadence (step/min)** | 35.13 | | | | | 35.13 | | | | |
| **Step Speed (m/s)** | 0.4706 | 0.4788 | 0.2293 | | | 0.4768 | 0.4852 | 0.2354 | | |
| **Step length (m)** | 0.5032 | 0.1258 | 0.0158 | 0.3286 | 0.9242 | 0.6996 | 0.1368 | 0.0187 | 0.4518 | 1.1501 |
| **Step time(s)** | 0.6128 | 0.1532 | 0.0235 | 0.4002 | 1.1255 | 0.8521 | 0.1666 | 0.0277 | 0.5503 | 1.4007 |
| **Step Ratio (Step length/cadence)** | 0.01 | | | | | 0.01 | | | | |
| | | | | | | | | | | |
| **Stance Time (s)** | 0.7270 | 0.2032 | 0.0413 | | | 0.7204 | 0.0890 | 0.0079 | | |
| **Swing Length (m)** | 1.4004 | 0.8569 | 0.7343 | | | 1.4550 | 1.0381 | 1.0776 | | |
| **Swing Time (s)** | 0.7379 | 0.1442 | 0.0208 | | | 0.7254 | 0.1441 | 0.0208 | | |
| **Swing Velocity (m/s)** | 1.8980 | 1.8980 | 1.8980 | | | 1.9719 | 2.0059 | 2.0059 | | |

| Young Participant 3 | Age | Height (m) | Weight (Kg) | Gender | | | | | | |
|---|---|---|---|---|---|---|---|---|---|---|
| | 23.0000 | 1.7200 | 63.0000 | Male | | | | | | |
| | | | | | | | | | | |
| **Total Time (s)** | 55.5250 | | | | | | | | | |
| | **Actual** | **Right Leg** | **Accuracy** | **Left Leg** | **Accuracy** | | | | | |
| **Total Distance (m)** | 33.3800 | 32.9581 | 98.7360 | 32.8014 | 98.2667 | | | | | |
| **Estimated Velocity (m/s)** | 0.6012 | 0.5936 | 98.7360 | 0.5908 | 98.2667 | | | | | |
| **Detected Stride Number** | 30.0000 | 30.0000 | 100.0000 | 30.0000 | 100.0000 | | | | | |
| **Detected Step Number** | 30.0000 | 30.0000 | 100.0000 | 30.0000 | 100.0000 | | | | | |
| | | | | | | | | | | |
| | **Right** | | | | | **Left** | | | | |
| **Gait Features** | **Mean** | **StaDev** | **Variance** | **MinRange** | **MaxRange** | **Mean** | **StaDev** | **Variance** | **MinRange** | **MaxRange** |
| **Stride Length (m)** | 1.0946 | 0.1369 | 0.0188 | 0.8612 | 1.4603 | 1.0944 | 0.1328 | 0.0176 | 0.7952 | 1.3064 |
| **Stride Time (s)** | 1.4873 | 0.1829 | 0.0335 | 1.1755 | 1.9759 | 1.4707 | 0.1754 | 0.0308 | 1.0755 | 1.7508 |
| **Stride Velocity (m/s)** | 0.7359 | 0.7359 | 0.7359 | | | 0.7441 | 0.7441 | 0.7441 | | |
| | | | | | | | | | | |
| **Cadence (step/min)** | 32.41 | | | | | 32.41 | | | | |
| **Step Speed (m/s)** | 0.7359 | 0.7485 | 0.5603 | | | 0.7441 | 0.7570 | 0.5731 | | |
| **Step length (m)** | 0.5067 | 0.1101 | 0.0121 | 0.2556 | 0.6766 | 0.3874 | 0.1145 | 0.0131 | 0.2105 | 0.7368 |
| **Step time(s)** | 0.8429 | 0.1831 | 0.0335 | 0.4252 | 1.1255 | 0.6445 | 0.1904 | 0.0363 | 0.3502 | 1.2256 |
| **Step Ratio (Step length/cadence)** | 0.01 | | | | | 0.01 | | | | |
| | | | | | | | | | | |
| **Stance Time (s)** | 0.7879 | 0.1227 | 0.0150 | | | 0.6961 | 0.1387 | 0.0192 | | |
| **Swing Length (m)** | 1.1076 | 0.5979 | 0.3574 | | | 1.0975 | 0.6184 | 0.3825 | | |
| **Swing Time (s)** | 0.6995 | 0.1300 | 0.0169 | | | 0.7745 | 0.1389 | 0.0193 | | |
| **Swing Velocity (m/s)** | 1.5834 | 1.5834 | 1.5834 | | | 1.5690 | 1.4170 | 1.4170 | | |

| Young Participant 4 | Age | Height (m) | Weight (Kg) | Gender | | | | | | |
|---|---|---|---|---|---|---|---|---|---|---|
| | 25.0000 | 1.5500 | 63.0000 | Male | | | | | | |
| | | | | | | | | | | |
| **Total Time (s)** | 59.9000 | | | | | | | | | |
| | **Actual** | **Right Leg** | **Accuracy** | **Left Leg** | **Accuracy** | | | | | |
| **Total Distance (m)** | 38.5800 | 38.7624 | 99.5271 | 38.0767 | 98.6954 | | | | | |
| **Estimated Velocity (m/s)** | 0.6441 | 0.6471 | 99.5271 | 0.6357 | 98.6954 | | | | | |
| **Detected Stride Number** | 30.0000 | 30.0000 | 100.0000 | 30.0000 | 100.0000 | | | | | |
| **Detected Step Number** | 30.0000 | 30.0000 | 100.0000 | 30.0000 | 100.0000 | | | | | |
| | | | | | | | | | | |
| | **Right** | | | | | **Left** | | | | |
| **Gait Features** | **Mean** | **StaDev** | **Variance** | **MinRange** | **MaxRange** | **Mean** | **StaDev** | **Variance** | **MinRange** | **MaxRange** |
| **Stride Length (m)** | 1.2661 | 0.1447 | 0.0209 | 1.0277 | 1.6032 | 1.2661 | 0.1546 | 0.0239 | 0.9671 | 1.5638 |
| **Stride Time (s)** | 1.5657 | 0.1761 | 0.0310 | 1.2755 | 1.9758 | 1.5640 | 0.1879 | 0.0353 | 1.2005 | 1.9258 |
| **Stride Velocity (m/s)** | 0.8087 | 0.8087 | 0.8087 | | | 0.8095 | 0.8095 | 0.8095 | | |
| | | | | | | | | | | |
| **Cadence (step/min)** | 30.05 | | | | | 30.05 | | | | |
| **Step Speed (m/s)** | 0.8087 | 0.8218 | 0.6754 | | | 0.8095 | 0.8227 | 0.6768 | | |
| **Step length (m)** | 0.4328 | 0.1033 | 0.0107 | 0.1450 | 0.5960 | 0.5756 | 0.1141 | 0.0130 | 0.3383 | 0.8376 |
| **Step time(s)** | 0.6719 | 0.1604 | 0.0257 | 0.2251 | 0.9254 | 0.8937 | 0.1771 | 0.0314 | 0.5252 | 1.3005 |
| **Step Ratio (Step length/cadence)** | 0.01 | | | | | 0.01 | | | | |
| | | | | | | | | | | |
| **Stance Time (s)** | 0.7153 | 0.1611 | 0.0260 | | | 0.7845 | 0.1296 | 0.0168 | | |
| **Swing Length (m)** | 1.2961 | 0.4891 | 0.2393 | | | 1.2750 | 0.7640 | 0.5837 | | |
| **Swing Time (s)** | 0.8504 | 0.1279 | 0.0163 | | | 0.7795 | 0.1601 | 0.0256 | | |
| **Swing Velocity (m/s)** | 1.5241 | 1.5241 | 1.5241 | | | 1.4993 | 1.6356 | 1.6356 | | |

| Young Participant 5 | Age | Height (m) | Weight (Kg) | Gender | | | | | | |
|---|---|---|---|---|---|---|---|---|---|---|
| | 26.0000 | 1.6500 | 61.0000 | Male | | | | | | |
| | | | | | | | | | | |
| **Total Time (s)** | 51.9000 | | | | | | | | | |
| | **Actual** | **Right Leg** | **Accuracy** | **Left Leg** | **Accuracy** | | | | | |
| **Total Distance (m)** | 45.3600 | 43.8421 | 96.6537 | 45.4254 | 99.8559 | | | | | |
| **Estimated Velocity (m/s)** | 0.8740 | 0.8447 | 96.6537 | 0.8752 | 99.8559 | | | | | |
| **Detected Stride Number** | 30.0000 | 30.0000 | 100.0000 | 30.0000 | 100.0000 | | | | | |
| **Detected Step Number** | 30.0000 | 30.0000 | 100.0000 | 30.0000 | 100.0000 | | | | | |
| | | | | | | | | | | |
| | **Right** | | | | | **Left** | | | | |
| **Gait Features** | **Mean** | **StaDev** | **Variance** | **MinRange** | **MaxRange** | **Mean** | **StaDev** | **Variance** | **MinRange** | **MaxRange** |
| **Stride Length (m)** | 1.4856 | 0.2062 | 0.0425 | 1.1190 | 2.2107 | 1.4855 | 0.1590 | 0.0253 | 1.2348 | 1.8660 |
| **Stride Time (s)** | 1.3865 | 0.1890 | 0.0357 | 1.0505 | 2.0510 | 1.3790 | 0.1449 | 0.0210 | 1.1506 | 1.7258 |
| **Stride Velocity (m/s)** | 1.0715 | 1.0715 | 1.0715 | | | 1.0772 | 1.0772 | 1.0772 | | |
| | | | | | | | | | | |
| **Cadence (step/min)** | 34.68 | | | | | 34.68 | | | | |
| **Step Speed (m/s)** | 1.0715 | 1.0912 | 1.1907 | | | 1.0772 | 1.0971 | 1.2037 | | |
| **Step length (m)** | 0.5727 | 0.1710 | 0.0292 | 0.0219 | 0.9400 | 0.6405 | 0.1883 | 0.0355 | 0.3279 | 1.3116 |
| **Step time(s)** | 0.6553 | 0.1957 | 0.0383 | 0.0250 | 1.0755 | 0.7329 | 0.2155 | 0.0464 | 0.3752 | 1.5007 |
| **Step Ratio (Step length/cadence)** | 0.01 | | | | | 0.01 | | | | |
| | | | | | | | | | | |
| **Stance Time (s)** | 0.7420 | 0.1313 | 0.0172 | | | 0.7879 | 0.1266 | 0.0160 | | |
| **Swing Length (m)** | 1.4712 | 1.0718 | 1.1487 | | | 1.5330 | 1.1008 | 1.2118 | | |
| **Swing Time (s)** | 0.6445 | 0.1366 | 0.0187 | | | 0.5911 | 0.0980 | 0.0096 | | |
| **Swing Velocity (m/s)** | 2.2827 | 2.2827 | 2.2827 | | | 2.3786 | 2.5933 | 2.5933 | | |

| Young Participant 6 | Age | Height (m) | Weight (Kg) | Gender | | | | | | |
|---|---|---|---|---|---|---|---|---|---|---|
| | 25.0000 | 1.7200 | 60.0000 | Male | | | | | | |
| | | | | | | | | | | |
| **Total Time (s)** | 56.5250 | | | | | | | | | |
| | **Actual** | **Right Leg** | **Accuracy** | **Left Leg** | **Accuracy** | | | | | |
| **Total Distance (m)** | 34.8600 | 34.9764 | 99.6662 | 35.4092 | 98.4245 | | | | | |
| **Estimated Velocity (m/s)** | 0.6167 | 0.6188 | 99.6662 | 0.6264 | 98.4245 | | | | | |
| **Detected Stride Number** | 30.0000 | 30.0000 | 100.0000 | 30.0000 | 100.0000 | | | | | |
| **Detected Step Number** | 30.0000 | 30.0000 | 100.0000 | 30.0000 | 100.0000 | | | | | |
| | | | | | | | | | | |
| | **Right** | | | | | **Left** | | | | |
| **Gait Features** | **Mean** | **StaDev** | **Variance** | **MinRange** | **MaxRange** | **Mean** | **StaDev** | **Variance** | **MinRange** | **MaxRange** |
| **Stride Length (m)** | 1.1437 | 0.1515 | 0.0230 | 0.9089 | 1.4580 | 1.1431 | 0.1315 | 0.0173 | 0.9169 | 1.4631 |
| **Stride Time (s)** | 1.5357 | 0.2002 | 0.0401 | 1.2255 | 1.9509 | 1.4907 | 0.1686 | 0.0284 | 1.2005 | 1.9008 |
| **Stride Velocity (m/s)** | 0.7447 | 0.7447 | 0.7447 | | | 0.7669 | 0.7669 | 0.7669 | | |
| | | | | | | | | | | |
| **Cadence (step/min)** | 38.14 | | | | | 38.14 | | | | |
| **Step Speed (m/s)** | 0.7447 | 0.7571 | 0.5732 | | | 0.7669 | 0.7800 | 0.6083 | | |
| **Step length (m)** | 0.4190 | 0.1484 | 0.0220 | 0.1542 | 0.9255 | 0.5280 | 0.1197 | 0.0143 | 0.1542 | 0.7095 |
| **Step time(s)** | 0.6795 | 0.2406 | 0.0579 | 0.2501 | 1.5007 | 0.8562 | 0.1940 | 0.0376 | 0.2501 | 1.1505 |
| **Step Ratio (Step length/cadence)** | 0.01 | | | | | 0.01 | | | | |
| | | | | | | | | | | |
| **Stance Time (s)** | 0.7328 | 0.1617 | 0.0262 | | | 0.7695 | 0.1000 | 0.0100 | | |
| **Swing Length (m)** | 1.1707 | 0.5774 | 0.3334 | | | 1.1844 | 0.8454 | 0.7148 | | |
| **Swing Time (s)** | 0.8029 | 0.1236 | 0.0153 | | | 0.7212 | 0.1451 | 0.0211 | | |
| **Swing Velocity (m/s)** | 1.4582 | 1.4582 | 1.4582 | | | 1.4752 | 1.6423 | 1.6423 | | |

| Young Participant 7 | Age | Height (m) | Weight (Kg) | Gender | | | | | | |
|---|---|---|---|---|---|---|---|---|---|---|
| | 29.0000 | 1.6500 | 62.0000 | Male | | | | | | |
| | | | | | | | | | | |
| **Total Time (s)** | 48.6750 | | | | | | | | | |
| | **Actual** | **Right Leg** | **Accuracy** | **Left Leg** | **Accuracy** | | | | | |
| **Total Distance (m)** | 33.9800 | 32.8346 | 96.6292 | 34.3799 | 98.8230 | | | | | |
| **Estimated Velocity (m/s)** | 0.6981 | 0.6746 | 96.6292 | 0.7063 | 98.8230 | | | | | |
| **Detected Stride Number** | 30.0000 | 30.0000 | 100.0000 | 30.0000 | 100.0000 | | | | | |
| **Detected Step Number** | 30.0000 | 30.0000 | 100.0000 | 30.0000 | 100.0000 | | | | | |
| | | | | | | | | | | |
| | **Right** | | | | | **Left** | | | | |
| **Gait Features** | **Mean** | **StaDev** | **Variance** | **MinRange** | **MaxRange** | **Mean** | **StaDev** | **Variance** | **MinRange** | **MaxRange** |
| **Stride Length (m)** | 1.1133 | 0.2098 | 0.0440 | 0.8028 | 1.6860 | 1.1126 | 0.2106 | 0.0443 | 0.7283 | 1.6230 |
| **Stride Time (s)** | 1.4124 | 0.2615 | 0.0684 | 1.0255 | 2.1261 | 1.3624 | 0.2531 | 0.0641 | 0.9005 | 1.9760 |
| **Stride Velocity (m/s)** | 0.7882 | 0.7882 | 0.7882 | | | 0.8166 | 0.8166 | 0.8166 | | |
| | | | | | | | | | | |
| **Cadence (step/min)** | 36.98 | | | | | 36.98 | | | | |
| **Step Speed (m/s)** | 0.7882 | 0.8024 | 0.6439 | | | 0.8166 | 0.8319 | 0.6921 | | |
| **Step length (m)** | 0.4784 | 0.1638 | 0.0268 | 0.1222 | 0.9429 | 0.6112 | 0.3713 | 0.1379 | 0.0349 | 1.5715 |
| **Step time(s)** | 0.6854 | 0.2347 | 0.0551 | 0.1751 | 1.3507 | 0.8754 | 0.5319 | 0.2829 | 0.0500 | 2.2512 |
| **Step Ratio (Step length/cadence)** | 0.01 | | | | | 0.01 | | | | |
| | | | | | | | | | | |
| **Stance Time (s)** | 0.6428 | 0.2228 | 0.0496 | | | 0.6595 | 0.2127 | 0.0452 | | |
| **Swing Length (m)** | 1.0983 | 0.7311 | 0.5344 | | | 1.1511 | 0.7527 | 0.5666 | | |
| **Swing Time (s)** | 0.7696 | 0.1448 | 0.0210 | | | 0.7029 | 0.1485 | 0.0221 | | |
| **Swing Velocity (m/s)** | 1.4271 | 1.4271 | 1.4271 | | | 1.4957 | 1.6377 | 1.6377 | | |

| Young Participant 8 | Age | Height (m) | Weight (Kg) | Gender | | | | | | |
|---|---|---|---|---|---|---|---|---|---|---|
| | 20.0000 | 1.5700 | 59.0000 | Male | | | | | | |
| | | | | | | | | | | |
| **Total Time (s)** | 47.0000 | | | | | | | | | |
| | **Actual** | **Right Leg** | **Accuracy** | **Left Leg** | **Accuracy** | | | | | |
| **Total Distance (m)** | 40.0400 | 40.8586 | 97.9555 | 39.5247 | 98.7130 | | | | | |
| **Estimated Velocity (m/s)** | 0.8519 | 0.8693 | 97.9555 | 0.8410 | 98.7130 | | | | | |
| **Detected Stride Number** | 30.0000 | 30.0000 | 100.0000 | 30.0000 | 100.0000 | | | | | |
| **Detected Step Number** | 30.0000 | 30.0000 | 100.0000 | 30.0000 | 100.0000 | | | | | |
| | | | | | | | | | | |
| | **Right** | | | | | **Left** | | | | |
| **Gait Features** | **Mean** | **StaDev** | **Variance** | **MinRange** | **MaxRange** | **Mean** | **StaDev** | **Variance** | **MinRange** | **MaxRange** |
| **Stride Length (m)** | 1.3106 | 0.2313 | 0.0535 | 0.9942 | 2.1126 | 1.3086 | 0.2375 | 0.0564 | 0.8622 | 1.7784 |
| **Stride Time (s)** | 1.3440 | 0.2328 | 0.0542 | 1.0255 | 2.1511 | 1.2398 | 0.2205 | 0.0486 | 0.8254 | 1.6759 |
| **Stride Velocity (m/s)** | 0.9751 | 0.9751 | 0.9751 | | | 1.0555 | 1.0555 | 1.0555 | | |
| | | | | | | | | | | |
| **Cadence (step/min)** | 38.29 | | | | | 38.29 | | | | |
| **Step Speed (m/s)** | 0.9751 | 0.9936 | 0.9873 | | | 1.0555 | 1.0772 | 1.1604 | | |
| **Step length (m)** | 0.6933 | 0.1765 | 0.0312 | 0.1065 | 1.0868 | 0.4518 | 0.2975 | 0.0885 | 0.0639 | 1.7260 |
| **Step time(s)** | 0.8138 | 0.2072 | 0.0429 | 0.1251 | 1.2757 | 0.5303 | 0.3492 | 0.1219 | 0.0750 | 2.0261 |
| **Step Ratio (Step length/cadence)** | 0.01 | | | | | 0.01 | | | | |
| | | | | | | | | | | |
| **Stance Time (s)** | 0.7221 | 0.2019 | 0.0408 | | | 0.5311 | 0.1529 | 0.0234 | | |
| **Swing Length (m)** | 1.3743 | 0.7833 | 0.6135 | | | 1.3201 | 0.8302 | 0.6893 | | |
| **Swing Time (s)** | 0.6220 | 0.1006 | 0.0101 | | | 0.7087 | 0.1698 | 0.0288 | | |
| **Swing Velocity (m/s)** | 2.2095 | 2.2095 | 2.2095 | | | 2.1224 | 1.8627 | 1.8627 | | |

| **Young Participant 9** | Age | Height (m) | Weight (Kg) | Gender | | | | | | |
|---|---|---|---|---|---|---|---|---|---|---|
| | 28.0000 | 1.7100 | 63.0000 | Male | | | | | | |
| | | | | | | | | | | |
| **Total Time (s)** | 47.6250 | | | | | | | | | |
| | **Actual** | **Right Leg** | **Accuracy** | **Left Leg** | **Accuracy** | | | | | |
| **Total Distance (m)** | 42.0600 | 41.9621 | 99.7673 | 41.7334 | 99.2236 | | | | | |
| **Estimated Velocity (m/s)** | 0.8831 | 0.8811 | 99.7673 | 0.8763 | 99.2236 | | | | | |
| **Detected Stride Number** | 30.0000 | 30.0000 | 100.0000 | 30.0000 | 100.0000 | | | | | |
| **Detected Step Number** | 30.0000 | 30.0000 | 100.0000 | 30.0000 | 100.0000 | | | | | |
| | | | | | | | | | | |
| | | | **Right** | | | | | | **Left** | |
| **Gait Features** | **Mean** | **StaDev** | **Variance** | **MinRange** | **MaxRange** | **Mean** | **StaDev** | **Variance** | **MinRange** | **MaxRange** |
| **Stride Length (m)** | 1.3761 | 0.1767 | 0.0312 | 1.1266 | 2.0118 | 1.3762 | 0.2322 | 0.0539 | 1.1476 | 2.3485 |
| **Stride Time (s)** | 1.3082 | 0.1648 | 0.0271 | 1.0756 | 1.9010 | 1.3149 | 0.2176 | 0.0474 | 1.1006 | 2.2262 |
| **Stride Velocity (m/s)** | 1.0519 | 1.0519 | 1.0519 | | | 1.0467 | 1.0467 | 1.0467 | | |
| | | | | | | | | | | |
| **Cadence (step/min)** | 37.79 | | | | | 37.79 | | | | |
| **Step Speed (m/s)** | 1.0519 | 1.0724 | 1.1500 | | | 1.0467 | 1.0670 | 1.1384 | | |
| **Step length (m)** | 0.5795 | 0.1636 | 0.0268 | 0.0442 | 0.9720 | 0.5758 | 0.2088 | 0.0436 | 0.2651 | 1.4801 |
| **Step time(s)** | 0.6562 | 0.1853 | 0.0343 | 0.0500 | 1.1006 | 0.6520 | 0.2364 | 0.0559 | 0.3002 | 1.6759 |
| **Step Ratio (Step length/cadence)** | 0.01 | | | | | 0.01 | | | | |
| | | | | | | | | | | |
| **Stance Time (s)** | 0.6862 | 0.1366 | 0.0187 | | | 0.6345 | 0.1044 | 0.0109 | | |
| **Swing Length (m)** | 1.4099 | 0.5759 | 0.3317 | | | 1.3937 | 2.3612 | 5.5754 | | |
| **Swing Time (s)** | 0.6220 | 0.1019 | 0.0104 | | | 0.6804 | 0.1955 | 0.0382 | | |
| **Swing Velocity (m/s)** | 2.2667 | 2.2667 | 2.2667 | | | 2.2407 | 2.0485 | 2.0485 | | |

| **Young Participant 10** | Age | Height (m) | Weight (Kg) | Gender | | | | | | |
|---|---|---|---|---|---|---|---|---|---|---|
| | 35.0000 | 1.7500 | 73.0000 | Male | | | | | | |
| | | | | | | | | | | |
| **Total Time (s)** | 48.0500 | | | | | | | | | |
| | **Actual** | **Right Leg** | **Accuracy** | **Left Leg** | **Accuracy** | | | | | |
| **Total Distance (m)** | 28.7600 | 26.5916 | 92.4603 | 28.7914 | 99.8909 | | | | | |
| **Estimated Velocity (m/s)** | 0.5985 | 0.5534 | 92.4603 | 0.5992 | 99.8909 | | | | | |
| **Detected Stride Number** | 30.0000 | 30.0000 | 100.0000 | 30.0000 | 100.0000 | | | | | |
| **Detected Step Number** | 30.0000 | 30.0000 | 100.0000 | 30.0000 | 100.0000 | | | | | |
| | | | | | | | | | | |
| | **Right** | | | | | **Left** | | | | |
| **Gait Features** | **Mean** | **StaDev** | **Variance** | **MinRange** | **MaxRange** | **Mean** | **StaDev** | **Variance** | **MinRange** | **MaxRange** |
| **Stride Length (m)** | 0.9404 | 0.1343 | 0.0180 | 0.7181 | 1.3416 | 0.9404 | 0.0938 | 0.0088 | 0.8487 | 1.2447 |
| **Stride Time (s)** | 1.2698 | 0.1778 | 0.0316 | 0.9755 | 1.8009 | 1.2723 | 0.1244 | 0.0155 | 1.1506 | 1.6759 |
| **Stride Velocity (m/s)** | 0.7406 | 0.7406 | 0.7406 | | | 0.7391 | 0.7391 | 0.7391 | | |
| | | | | | | | | | | |
| **Cadence (step/min)** | 37.46 | | | | | 37.46 | | | | |
| **Step Speed (m/s)** | 0.7406 | 0.7555 | 0.5707 | | | 0.7391 | 0.7540 | 0.5685 | | |
| **Step length (m)** | 0.3498 | 0.0818 | 0.0067 | 0.1647 | 0.5539 | 0.4102 | 0.0994 | 0.0099 | 0.2695 | 0.7037 |
| **Step time(s)** | 0.5845 | 0.1366 | 0.0187 | 0.2751 | 0.9255 | 0.6854 | 0.1661 | 0.0276 | 0.4502 | 1.1756 |
| **Step Ratio (Step length/cadence)** | 0.01 | | | | | 0.01 | | | | |
| | | | | | | | | | | |
| **Stance Time (s)** | 0.6462 | 0.1190 | 0.0142 | | | 0.6412 | 0.0909 | 0.0083 | | |
| **Swing Length (m)** | 0.8935 | 0.5170 | 0.2673 | | | 0.9670 | 0.4313 | 0.1860 | | |
| **Swing Time (s)** | 0.6237 | 0.1275 | 0.0163 | | | 0.6312 | 0.1292 | 0.0167 | | |
| **Swing Velocity (m/s)** | 1.4327 | 1.4327 | 1.4327 | | | 1.5506 | 1.5322 | 1.5322 | | |

| Elderly Participant 1 | Age | Height (m) | Weight (Kg) | Gender | | | | | | |
|---|---|---|---|---|---|---|---|---|---|---|
| | 67.0000 | 1.5700 | 68.0000 | Male | | | | | | |
| | | | | | | | | | | |
| **Total Time (s)** | 99.3000 | | | | | | | | | |
| | **Actual** | **Right Leg** | **Accuracy** | **Left Leg** | **Accuracy** | | | | | |
| **Total Distance (m)** | 21.0300 | 20.5910 | 97.9123 | 20.4708 | 97.3409 | | | | | |
| **Estimated Velocity (m/s)** | 0.2118 | 0.2074 | 97.9123 | 0.2062 | 97.3409 | | | | | |
| **Detected Stride Number** | 30.0000 | 30.0000 | 100.0000 | 30.0000 | 100.0000 | | | | | |
| **Detected Step Number** | 30.0000 | 30.0000 | 100.0000 | 30.0000 | 100.0000 | | | | | |
| | | | | | | | | | | |
| | **Right** | | | | | **Left** | | | | |
| **Gait Features** | **Mean** | **StaDev** | **Variance** | **MinRange** | **MaxRange** | **Mean** | **StaDev** | **Variance** | **MinRange** | **MaxRange** |
| **Stride Length (m)** | 0.6889 | 0.0702 | 0.0049 | 0.5630 | 0.8382 | 0.6891 | 0.0902 | 0.0081 | 0.4931 | 1.0478 |
| **Stride Time (s)** | 2.8047 | 0.2805 | 0.0787 | 2.3012 | 3.4017 | 2.8464 | 0.3660 | 0.1340 | 2.0510 | 4.3022 |
| **Stride Velocity (m/s)** | 0.2456 | 0.2456 | 0.2456 | | | 0.2421 | 0.2421 | 0.2421 | | |
| | | | | | | | | | | |
| **Cadence (step/min)** | 11.13 | | | | | 11.13 | | | | |
| **Step Speed (m/s)** | 0.2456 | 0.2501 | 0.0625 | | | 0.2421 | 0.2464 | 0.0607 | | |
| **Step length (m)** | 0.2719 | 0.0643 | 0.0041 | 0.1377 | 0.4556 | 0.3221 | 0.0541 | 0.0029 | 0.2331 | 0.4556 |
| **Step time(s)** | 1.2840 | 0.3037 | 0.0922 | 0.6503 | 2.1511 | 1.5208 | 0.2556 | 0.0653 | 1.1006 | 2.1511 |
| **Step Ratio (Step length/cadence)** | 0.02 | | | | | 0.02 | | | | |
| | | | | | | | | | | |
| **Stance Time (s)** | 1.3457 | 0.2315 | 0.0535 | | | 1.4274 | 0.3481 | 0.1216 | | |
| **Swing Length (m)** | 0.6896 | 0.3485 | 0.1215 | | | 0.6853 | 0.4392 | 0.1929 | | |
| **Swing Time (s)** | 1.4591 | 0.2341 | 0.0549 | | | 1.4194 | 0.2071 | 0.0418 | | |
| **Swing Velocity (m/s)** | 0.4726 | 0.4717 | 0.4717 | | | 0.4688 | 0.4831 | 0.4831 | | |

| Elderly Participant 2 | Age | Height (m) | Weight (Kg) | Gender | | | | | | |
|---|---|---|---|---|---|---|---|---|---|---|
| | 63.0000 | 1.7300 | 62.0000 | Female | | | | | | |
| | | | | | | | | | | |
| **Total Time (s)** | 73.4500 | | | | | | | | | |
| | **Actual** | **Right Leg** | **Accuracy** | **Left Leg** | **Accuracy** | | | | | |
| **Total Distance (m)** | 26.8200 | 26.4892 | 98.7666 | 25.2723 | 94.2292 | | | | | |
| **Estimated Velocity (m/s)** | 0.3651 | 0.3606 | 98.7666 | 0.3441 | 94.2292 | | | | | |
| **Detected Stride Number** | 30.0000 | 30.0000 | 100.0000 | 30.0000 | 100.0000 | | | | | |
| **Detected Step Number** | 30.0000 | 30.0000 | 100.0000 | 30.0000 | 100.0000 | | | | | |
| | | | | | | | | | | |
| | **Right** | | | | | **Left** | | | | |
| **Gait Features** | **Mean** | **StaDev** | **Variance** | **MinRange** | **MaxRange** | **Mean** | **StaDev** | **Variance** | **MinRange** | **MaxRange** |
| **Stride Length (m)** | 0.6929 | 0.1353 | 0.0183 | 0.4184 | 0.9373 | 0.6930 | 0.1357 | 0.0184 | 0.4527 | 0.9548 |
| **Stride Time (s)** | 2.0957 | 0.4044 | 0.1635 | 1.2754 | 2.8260 | 2.1307 | 0.4124 | 0.1701 | 1.4005 | 2.9260 |
| **Stride Velocity (m/s)** | 0.3306 | 0.3306 | 0.3306 | | | 0.3253 | 0.3253 | 0.3253 | | |
| | | | | | | | | | | |
| **Cadence (step/min)** | 24.50 | | | | | 24.50 | | | | |
| **Step Speed (m/s)** | 0.3306 | 0.3346 | 0.1120 | | | 0.3253 | 0.3291 | 0.1083 | | |
| **Step length (m)** | 0.1820 | 0.1408 | 0.0198 | -0.1461 | 0.5662 | 0.5832 | 0.1458 | 0.0212 | 0.2922 | 0.7762 |
| **Step time(s)** | 0.4985 | 0.3856 | 0.1487 | -0.4001 | 1.5505 | 1.5972 | 0.3992 | 0.1594 | 0.8003 | 2.1257 |
| **Step Ratio (Step length/cadence)** | 0.02 | | | | | 0.02 | | | | |
| | | | | | | | | | | |
| **Stance Time (s)** | 0.9853 | 0.2655 | 0.0705 | | | 1.0870 | 0.2469 | 0.0609 | | |
| **Swing Length (m)** | 0.8820 | 0.6585 | 0.4336 | | | 0.8433 | 0.6916 | 0.4783 | | |
| **Swing Time (s)** | 1.1104 | 0.2307 | 0.0532 | | | 1.0437 | 0.2568 | 0.0659 | | |
| **Swing Velocity (m/s)** | 0.7943 | 0.7943 | 0.7943 | | | 0.7595 | 0.8080 | 0.8080 | | |

| **Elderly Participant 3** | Age | Height (m) | Weight (Kg) | Gender | | | | | | |
|---|---|---|---|---|---|---|---|---|---|---|
| | 86.0000 | 1.4200 | 64.0000 | Male | | | | | | |
| | | | | | | | | | | |
| **Total Time (s)** | 96.5750 | | | | | | | | | |
| | **Actual** | **Right Leg** | **Accuracy** | **Left Leg** | **Accuracy** | | | | | |
| **Total Distance (m)** | 20.7000 | 20.8989 | 99.0393 | 20.6760 | 99.8842 | | | | | |
| **Estimated Velocity (m/s)** | 0.2143 | 0.2164 | 99.0393 | 0.2141 | 99.8842 | | | | | |
| **Detected Stride Number** | 26.0000 | 26.0000 | 100.0000 | 26.0000 | 100.0000 | | | | | |
| **Detected Step Number** | 26.0000 | 26.0000 | 100.0000 | 26.0000 | 100.0000 | | | | | |
| | | | | | | | | | | |
| | **Right** | | | | | **Left** | | | | |
| **Gait Features** | **Mean** | **StaDev** | **Variance** | **MinRange** | **MaxRange** | **Mean** | **StaDev** | **Variance** | **MinRange** | **MaxRange** |
| **Stride Length (m)** | 0.7901 | 0.1315 | 0.0173 | 0.6653 | 1.2490 | 0.7898 | 0.0780 | 0.0061 | 0.6586 | 0.9616 |
| **Stride Time (s)** | 3.1729 | 0.5238 | 0.2744 | 2.6757 | 5.0013 | 3.0239 | 0.2961 | 0.0877 | 2.5257 | 3.6760 |
| **Stride Velocity (m/s)** | 0.2490 | 0.2490 | 0.2490 | | | 0.2612 | 0.2612 | 0.2612 | | |
| | | | | | | | | | | |
| **Cadence (step/min)** | 18.63 | | | | | 18.63 | | | | |
| **Step Speed (m/s)** | 0.2490 | 0.2510 | 0.0630 | | | 0.2612 | 0.2634 | 0.0694 | | |
| **Step length (m)** | -0.1633 | 0.3575 | 0.1278 | -0.5682 | 0.4824 | 0.1952 | 0.3440 | 0.1183 | -0.2519 | 0.7236 |
| **Step time(s)** | -0.7617 | 1.6678 | 2.7816 | -2.6507 | 2.2506 | 0.9108 | 1.6050 | 2.5759 | -1.1753 | 3.3759 |
| **Step Ratio (Step length/cadence)** | 0.01 | | | | | 0.01 | | | | |
| | | | | | | | | | | |
| **Stance Time (s)** | 1.6427 | 0.4033 | 0.1626 | | | 1.5292 | 0.2439 | 0.0595 | | |
| **Swing Length (m)** | 0.8025 | 1.1141 | 1.2413 | | | 0.7953 | 0.5205 | 0.2709 | | |
| **Swing Time (s)** | 1.5302 | 0.3247 | 0.1054 | | | 1.4946 | 0.2224 | 0.0494 | | |
| **Swing Velocity (m/s)** | 0.5244 | 0.5244 | 0.5244 | | | 0.5198 | 0.5321 | 0.5321 | | |

| Elderly Participant 4 | Age | Height (m) | Weight (Kg) | Gender | | | | | | |
|---|---|---|---|---|---|---|---|---|---|---|
| | 73.0000 | 1.4400 | 65.0000 | Male | | | | | | |
| | | | | | | | | | | |
| **Total Time (s)** | 71.8250 | | | | | | | | | |
| | **Actual** | **Right Leg** | **Accuracy** | **Left Leg** | **Accuracy** | | | | | |
| **Total Distance (m)** | 24.6000 | 24.6303 | 99.8769 | 25.4147 | 96.6884 | | | | | |
| **Estimated Velocity (m/s)** | 0.3425 | 0.3429 | 99.8769 | 0.3538 | 96.6884 | | | | | |
| **Detected Stride Number** | 30.0000 | 30.0000 | 100.0000 | 30.0000 | 100.0000 | | | | | |
| **Detected Step Number** | 30.0000 | 30.0000 | 100.0000 | 30.0000 | 100.0000 | | | | | |
| | | | | | | | | | | |
| | **Right** | | | | | **Left** | | | | |
| **Gait Features** | **Mean** | **StaDev** | **Variance** | **MinRange** | **MaxRange** | **Mean** | **StaDev** | **Variance** | **MinRange** | **MaxRange** |
| **Stride Length (m)** | 0.6929 | 0.0989 | 0.0098 | 0.4836 | 0.9506 | 0.6928 | 0.1469 | 0.0216 | 0.4318 | 0.9990 |
| **Stride Time (s)** | 2.1032 | 0.2967 | 0.0881 | 1.4755 | 2.8760 | 2.0716 | 0.4340 | 0.1883 | 1.3005 | 2.9760 |
| **Stride Velocity (m/s)** | 0.3295 | 0.3295 | 0.3295 | | | 0.3344 | 0.3344 | 0.3344 | | |
| | | | | | | | | | | |
| **Cadence (step/min)** | 25.06 | | | | | 25.06 | | | | |
| **Step Speed (m/s)** | 0.3295 | 0.3334 | 0.1112 | | | 0.3344 | 0.3385 | 0.1146 | | |
| **Step length (m)** | 0.2484 | 0.1259 | 0.0158 | -0.0257 | 0.5311 | 0.4720 | 0.1252 | 0.0157 | 0.2313 | 0.7709 |
| **Step time(s)** | 0.7253 | 0.3675 | 0.1351 | -0.0750 | 1.5505 | 1.3780 | 0.3655 | 0.1336 | 0.6752 | 2.2508 |
| **Step Ratio (Step length/cadence)** | 0.01 | | | | | 0.01 | | | | |
| | | | | | | | | | | |
| **Stance Time (s)** | 1.1029 | 0.1900 | 0.0361 | | | 1.0204 | 0.2560 | 0.0655 | | |
| **Swing Length (m)** | 0.8217 | 0.3438 | 0.1182 | | | 0.8482 | 0.6649 | 0.4421 | | |
| **Swing Time (s)** | 1.0003 | 0.2136 | 0.0456 | | | 1.0512 | 0.2618 | 0.0685 | | |
| **Swing Velocity (m/s)** | 0.8214 | 0.8214 | 0.8214 | | | 0.8479 | 0.8069 | 0.8069 | | |

| Elderly Participant 5 | Age | Height (m) | Weight (Kg) | Gender | | | | | | |
|---|---|---|---|---|---|---|---|---|---|---|
| | 75.0000 | 1.3200 | 62.0000 | Male | | | | | | |
| | | | | | | | | | | |
| **Total Time (s)** | 105.6250 | | | | | | | | | |
| | **Actual** | **Right Leg** | **Accuracy** | **Left Leg** | **Accuracy** | | | | | |
| **Total Distance (m)** | 15.5000 | 20.1874 | 69.7587 | 19.0633 | 77.0108 | | | | | |
| **Estimated Velocity (m/s)** | 0.1467 | 0.1911 | 69.7587 | 0.1805 | 77.0108 | | | | | |
| **Detected Stride Number** | 26.0000 | 26.0000 | 100.0000 | 26.0000 | 100.0000 | | | | | |
| **Detected Step Number** | 26.0000 | 26.0000 | 100.0000 | 26.0000 | 100.0000 | | | | | |
| | | | | | | | | | | |
| | **Right** | | | | | **Left** | | | | |
| **Gait Features** | **Mean** | **StaDev** | **Variance** | **MinRange** | **MaxRange** | **Mean** | **StaDev** | **Variance** | **MinRange** | **MaxRange** |
| **Stride Length (m)** | 0.5917 | 0.1095 | 0.0120 | 0.4283 | 0.9947 | 0.5917 | 0.0937 | 0.0088 | 0.4640 | 0.8545 |
| **Stride Time (s)** | 3.2383 | 0.5948 | 0.3538 | 2.3506 | 5.4263 | 3.2460 | 0.5103 | 0.2604 | 2.5506 | 4.6761 |
| **Stride Velocity (m/s)** | 0.1827 | 0.1827 | 0.1827 | | | 0.1823 | 0.1823 | 0.1823 | | |
| | | | | | | | | | | |
| **Cadence (step/min)** | 17.04 | | | | | 17.04 | | | | |
| **Step Speed (m/s)** | 0.1827 | 0.1842 | 0.0339 | | | 0.1823 | 0.1837 | 0.0338 | | |
| **Step length (m)** | -0.0557 | 0.2005 | 0.0402 | -0.4257 | 0.2569 | 0.0546 | 0.2006 | 0.0402 | -0.2569 | 0.4257 |
| **Step time(s)** | -0.3799 | 1.3664 | 1.8669 | -2.9007 | 1.7504 | 0.3722 | 1.3671 | 1.8689 | -1.7504 | 2.9007 |
| **Step Ratio (Step length/cadence)** | 0.003 | | | | | 0.003 | | | | |
| | | | | | | | | | | |
| **Stance Time (s)** | 1.6196 | 0.3026 | 0.0916 | | | 1.6292 | 0.3636 | 0.1322 | | |
| **Swing Length (m)** | 0.7756 | 0.9104 | 0.8289 | | | 0.7327 | 0.8279 | 0.6855 | | |
| **Swing Time (s)** | 1.6187 | 0.4003 | 0.1602 | | | 1.6167 | 0.2991 | 0.0895 | | |
| **Swing Velocity (m/s)** | 0.4791 | 0.4791 | 0.4791 | | | 0.4527 | 0.4532 | 0.4532 | | |

| Elderly Participant 6 | Age | Height (m) | Weight (Kg) | Gender | | | | | | |
|---|---|---|---|---|---|---|---|---|---|---|
| | 62.0000 | 1.6800 | 59.0000 | Male | | | | | | |
| | | | | | | | | | | |
| **Total Time (s)** | 96.5750 | | | | | | | | | |
| | **Actual** | **Right Leg** | **Accuracy** | **Left Leg** | **Accuracy** | | | | | |
| **Total Distance (m)** | 20.7000 | 20.7377 | 99.8179 | 20.6760 | 99.8842 | | | | | |
| **Estimated Velocity (m/s)** | 0.2143 | 0.2147 | 99.8179 | 0.2141 | 99.8842 | | | | | |
| **Detected Stride Number** | 26.0000 | 26.0000 | 100.0000 | 26.0000 | 100.0000 | | | | | |
| **Detected Step Number** | 26.0000 | 26.0000 | 100.0000 | 26.0000 | 100.0000 | | | | | |
| | | | | | | | | | | |
| | **Right** | | | | | **Left** | | | | |
| **Gait Features** | **Mean** | **StaDev** | **Variance** | **MinRange** | **MaxRange** | **Mean** | **StaDev** | **Variance** | **MinRange** | **MaxRange** |
| **Stride Length (m)** | 0.7900 | 0.1314 | 0.0173 | 0.6737 | 1.2648 | 0.7898 | 0.0780 | 0.0061 | 0.6586 | 0.9616 |
| **Stride Time (s)** | 3.1335 | 0.5171 | 0.2674 | 2.6757 | 5.0013 | 3.0239 | 0.2961 | 0.0877 | 2.5257 | 3.6760 |
| **Stride Velocity (m/s)** | 0.2521 | 0.2521 | 0.2521 | | | 0.2612 | 0.2612 | 0.2612 | | |
| | | | | | | | | | | |
| **Cadence (step/min)** | 18.63 | | | | | 18.63 | | | | |
| **Step Speed (m/s)** | 0.2521 | 0.2542 | 0.0646 | | | 0.2612 | 0.2634 | 0.0694 | | |
| **Step length (m)** | -0.1633 | 0.3575 | 0.1278 | -0.5682 | 0.4824 | 0.1868 | 0.3330 | 0.1109 | -0.2519 | 0.5682 |
| **Step time(s)** | -0.7617 | 1.6678 | 2.7816 | -2.6507 | 2.2506 | 0.8714 | 1.5538 | 2.4142 | -1.1753 | 2.6507 |
| **Step Ratio (Step length/cadence)** | 0.01 | | | | | 0.01 | | | | |
| | | | | | | | | | | |
| **Stance Time (s)** | 1.6091 | 0.3821 | 0.1460 | | | 1.5292 | 0.2439 | 0.0595 | | |
| **Swing Length (m)** | 0.7961 | 1.0520 | 1.1068 | | | 0.7953 | 0.5205 | 0.2709 | | |
| **Swing Time (s)** | 1.5244 | 0.3275 | 0.1072 | | | 1.4946 | 0.2224 | 0.0494 | | |
| **Swing Velocity (m/s)** | 0.5223 | 0.5223 | 0.5223 | | | 0.5217 | 0.5321 | 0.5321 | | |

| **Elderly Participant 7** | Age | Height (m) | Weight (Kg) | Gender | | | | | | |
|---|---|---|---|---|---|---|---|---|---|---|
| | 71.0000 | 1.6500 | 64.0000 | Male | | | | | | |
| | | | | | | | | | | |
| **Total Time (s)** | 80.1000 | | | | | | | | | |
| | **Actual** | **Right Leg** | **Accuracy** | **Left Leg** | **Accuracy** | | | | | |
| **Total Distance (m)** | 23.7700 | 22.9554 | 96.5728 | 23.2947 | 98.0005 | | | | | |
| **Estimated Velocity (m/s)** | 0.2968 | 0.2866 | 96.5728 | 0.2908 | 98.0005 | | | | | |
| **Detected Stride Number** | 30.0000 | 30.0000 | 100.0000 | 30.0000 | 100.0000 | | | | | |
| **Detected Step Number** | 30.0000 | 30.0000 | 100.0000 | 30.0000 | 100.0000 | | | | | |
| | | | | | | | | | | |
| | **Right** | | | | | **Left** | | | | |
| **Gait Features** | **Mean** | **StaDev** | **Variance** | **MinRange** | **MaxRange** | **Mean** | **StaDev** | **Variance** | **MinRange** | **MaxRange** |
| **Stride Length (m)** | 0.6941 | 0.1794 | 0.0322 | 0.4334 | 0.9947 | 0.6939 | 0.1307 | 0.0171 | 0.4543 | 0.9453 |
| **Stride Time (s)** | 2.4683 | 0.6315 | 0.3988 | 1.5505 | 3.5261 | 2.3932 | 0.4462 | 0.1991 | 1.5755 | 3.2510 |
| **Stride Velocity (m/s)** | 0.2812 | 0.2812 | 0.2812 | | | 0.2899 | 0.2899 | 0.2899 | | |
| | | | | | | | | | | |
| **Cadence (step/min)** | 21.47 | | | | | 21.47 | | | | |
| **Step Speed (m/s)** | 0.2812 | 0.2841 | 0.0807 | | | 0.2899 | 0.2930 | 0.0859 | | |
| **Step length (m)** | 0.1680 | 0.1747 | 0.0305 | -0.2152 | 0.4750 | 0.5645 | 0.1912 | 0.0365 | 0.1484 | 1.0464 |
| **Step time(s)** | 0.5660 | 0.5888 | 0.3467 | -0.7252 | 1.6005 | 1.9023 | 0.6442 | 0.4150 | 0.5002 | 3.5261 |
| **Step Ratio (Step length/cadence)** | 0.02 | | | | | 0.02 | | | | |
| | | | | | | | | | | |
| **Stance Time (s)** | 1.2662 | 0.3561 | 0.1268 | | | 1.1787 | 0.2943 | 0.0866 | | |
| **Swing Length (m)** | 0.7653 | 0.9251 | 0.8558 | | | 0.7766 | 0.8934 | 0.7981 | | |
| **Swing Time (s)** | 1.2020 | 0.3521 | 0.1240 | | | 1.2145 | 0.2146 | 0.0460 | | |
| **Swing Velocity (m/s)** | 0.6366 | 0.6366 | 0.6366 | | | 0.6460 | 0.6394 | 0.6394 | | |

| **Elderly Participant 8** | Age | Height (m) | Weight (Kg) | Gender | | | | | | |
|---|---|---|---|---|---|---|---|---|---|---|
| | 67.0000 | 1.5800 | 60.0000 | Male | | | | | | |
| | | | | | | | | | | |
| **Total Time (s)** | 74.8000 | | | | | | | | | |
| | **Actual** | **Right Leg** | **Accuracy** | **Left Leg** | **Accuracy** | | | | | |
| **Total Distance (m)** | 26.2100 | 26.8913 | 97.4006 | 23.5050 | 89.6797 | | | | | |
| **Estimated Velocity (m/s)** | 0.3504 | 0.3595 | 97.4006 | 0.3142 | 89.6797 | | | | | |
| **Detected Stride Number** | 30.0000 | 30.0000 | 100.0000 | 30.0000 | 100.0000 | | | | | |
| **Detected Step Number** | 30.0000 | 30.0000 | 100.0000 | 30.0000 | 100.0000 | | | | | |
| | | | | | | | | | | |
| | **Right** | | | | | **Left** | | | | |
| **Gait Features** | **Mean** | **StaDev** | **Variance** | **MinRange** | **MaxRange** | **Mean** | **StaDev** | **Variance** | **MinRange** | **MaxRange** |
| **Stride Length (m)** | 0.6932 | 0.2528 | 0.0639 | 0.4953 | 1.9081 | 0.6926 | 0.1675 | 0.0280 | 0.4538 | 1.2217 |
| **Stride Time (s)** | 2.1599 | 0.7787 | 0.6064 | 1.5505 | 5.9020 | 2.0098 | 0.4799 | 0.2303 | 1.3254 | 3.5262 |
| **Stride Velocity (m/s)** | 0.3209 | 0.3209 | 0.3209 | | | 0.3446 | 0.3446 | 0.3446 | | |
| | | | | | | | | | | |
| **Cadence (step/min)** | 24.06 | | | | | 24.06 | | | | |
| **Step Speed (m/s)** | 0.3209 | 0.3247 | 0.1054 | | | 0.3446 | 0.3489 | 0.1218 | | |
| **Step length (m)** | 0.3444 | 0.3203 | 0.1026 | -0.7536 | 0.7010 | 0.3124 | 0.1583 | 0.0251 | 0.0526 | 0.5345 |
| **Step time(s)** | 0.9828 | 0.9140 | 0.8354 | -2.1507 | 2.0007 | 0.8604 | 0.4435 | 0.1967 | 0.1501 | 1.5255 |
| **Step Ratio (Step length/cadence)** | 0.01 | | | | | 0.01 | | | | |
| | | | | | | | | | | |
| **Stance Time (s)** | 1.0495 | 0.4316 | 0.1863 | | | 1.0278 | 0.2793 | 0.0780 | | |
| **Swing Length (m)** | 0.8962 | 1.3252 | 1.7561 | | | 0.7856 | 1.2665 | 1.6041 | | |
| **Swing Time (s)** | 1.1104 | 0.4352 | 0.1894 | | | 0.9820 | 0.2690 | 0.0724 | | |
| **Swing Velocity (m/s)** | 0.8071 | 0.8071 | 0.8071 | | | 0.7075 | 0.8000 | 0.8000 | | |

| Elderly Participant 9 | Age | Height (m) | Weight (Kg) | Gender | | | | | | |
|---|---|---|---|---|---|---|---|---|---|---|
| | 63.0000 | 1.2700 | 62.0000 | Female | | | | | | |
| | | | | | | | | | | |
| **Total Time (s)** | 66.7500 | | | | | | | | | |
| | **Actual** | **Right Leg** | **Accuracy** | **Left Leg** | **Accuracy** | | | | | |
| **Total Distance (m)** | 23.2600 | 27.9676 | 79.7611 | 22.9786 | 98.7900 | | | | | |
| **Estimated Velocity (m/s)** | 0.3485 | 0.4190 | 79.7611 | 0.3442 | 98.7900 | | | | | |
| **Detected Stride Number** | 30.0000 | 30.0000 | 100.0000 | 30.0000 | 100.0000 | | | | | |
| **Detected Step Number** | 30.0000 | 30.0000 | 100.0000 | 30.0000 | 100.0000 | | | | | |
| | | | | | | | | | | |
| | **Right** | | | | | **Left** | | | | |
| **Gait Features** | **Mean** | **StaDev** | **Variance** | **MinRange** | **MaxRange** | **Mean** | **StaDev** | **Variance** | **MinRange** | **MaxRange** |
| **Stride Length (m)** | 0.6927 | 0.1188 | 0.0141 | 0.4727 | 0.9368 | 0.6926 | 0.0968 | 0.0094 | 0.4697 | 0.8697 |
| **Stride Time (s)** | 2.0408 | 0.3457 | 0.1195 | 1.4005 | 2.7510 | 2.0166 | 0.2782 | 0.0774 | 1.3755 | 2.5259 |
| **Stride Velocity (m/s)** | 0.3394 | 0.3394 | 0.3394 | | | 0.3434 | 0.3434 | 0.3434 | | |
| | | | | | | | | | | |
| **Cadence (step/min)** | 26.99 | | | | | 26.99 | | | | |
| **Step Speed (m/s)** | 0.3394 | 0.3436 | 0.1181 | | | 0.3434 | 0.3478 | 0.1209 | | |
| **Step length (m)** | 0.8462 | 0.3802 | 0.1445 | 0.1307 | 1.3508 | 0.3622 | 0.1700 | 0.0289 | 0.0610 | 0.6275 |
| **Step time(s)** | 2.4284 | 1.0910 | 1.1903 | 0.3751 | 3.8765 | 1.0396 | 0.4878 | 0.2380 | 0.1751 | 1.8007 |
| **Step Ratio (Step length/cadence)** | 0.03 | | | | | 0.01 | | | | |
| | | | | | | | | | | |
| **Stance Time (s)** | 1.0621 | 0.2186 | 0.0478 | | | 0.9837 | 0.2181 | 0.0475 | | |
| **Swing Length (m)** | 0.9328 | 0.8875 | 0.7877 | | | 0.7679 | 1.2501 | 1.5628 | | |
| **Swing Time (s)** | 0.9787 | 0.2440 | 0.0595 | | | 1.0329 | 0.2003 | 0.0401 | | |
| **Swing Velocity (m/s)** | 0.9531 | 0.9531 | 0.9531 | | | 0.7846 | 0.7434 | 0.7434 | | |

| Elderly Participant 10 | Age | Height (m) | Weight (Kg) | Gender | | | | | | |
|---|---|---|---|---|---|---|---|---|---|---|
| | 67.0000 | 1.5700 | 68.0000 | Male | | | | | | |
| | | | | | | | | | | |
| **Total Time (s)** | 37.6000 | | | | | | | | | |
| | **Actual** | **Right Leg** | **Accuracy** | **Left Leg** | **Accuracy** | | | | | |
| **Total Distance (m)** | 22.3500 | 10.7724 | 48.1985 | 10.5724 | 47.3039 | | | | | |
| **Estimated Velocity (m/s)** | 0.5944 | 0.2865 | 48.1985 | 0.2812 | 47.3039 | | | | | |
| **Detected Stride Number** | 20.0000 | 20.0000 | 100.0000 | 20.0000 | 100.0000 | | | | | |
| **Detected Step Number** | 20.0000 | 20.0000 | 100.0000 | 20.0000 | 100.0000 | | | | | |
| | | | | | | | | | | |
| | **Right** | | | | | **Left** | | | | |
| **Gait Features** | **Mean** | **StaDev** | **Variance** | **MinRange** | **MaxRange** | **Mean** | **StaDev** | **Variance** | **MinRange** | **MaxRange** |
| **Stride Length (m)** | 1.0824 | 0.1372 | 0.0188 | 0.7758 | 1.4777 | 1.0842 | 0.2780 | 0.0773 | 0.9227 | 2.1188 |
| **Stride Time (s)** | 1.5170 | 0.1860 | 0.0346 | 1.1015 | 2.0527 | 1.5996 | 0.4074 | 0.1659 | 1.4019 | 3.1542 |
| **Stride Velocity (m/s)** | 0.7135 | 0.7135 | 0.7135 | | | 0.6778 | 0.6617 | 0.6617 | | |
| | | | | | | | | | | |
| **Cadence (step/min)** | 31.91 | | | | | 31.91 | | | | |
| **Step Speed (m/s)** | 0.7135 | 0.7379 | 0.5444 | | | 0.6778 | 0.6826 | 0.4659 | | |
| **Step length (m)** | 0.4955 | 0.1494 | 0.0223 | 0.2083 | 1.0119 | 0.4896 | 0.0870 | 0.0076 | -0.6250 | -0.2083 |
| **Step time(s)** | 0.8336 | 0.2513 | 0.0631 | 0.3505 | 1.7023 | 0.8236 | 0.1463 | 0.0214 | -1.0514 | -0.3505 |
| **Step Ratio (Step length/cadence)** | 0.01 | | | | | 0.01 | | | | |
| | | | | | | | | | | |
| **Stance Time (s)** | 0.8186 | 0.2151 | 0.0462 | | | 0.7911 | 0.2637 | 0.0696 | | |
| **Swing Length (m)** | 0.5294 | 0.2644 | 0.0699 | | | 0.5929 | 0.5208 | 0.2712 | | |
| **Swing Time (s)** | 0.6984 | 0.2642 | 0.0698 | | | 0.8486 | 0.1604 | 0.0257 | | |
| **Swing Velocity (m/s)** | 0.7579 | 0.7579 | 0.7579 | | | 0.8489 | 0.6986 | 0.6986 | | |
